# Supplementary material for: The genome of Acorus deciphers insights into early monocot evolution
Source: Nat Commun. 2023 Jun 20;14:3662. doi: 10.1038/s41467-023-38836-4 (PMC10281966; doi:10.1038/s41467-023-38836-4)
Supplement: Supplementary file 1 — Supplementary Information [file 41467_2023_38836_MOESM1_ESM.pdf]

Supplementary Note 1. The genome sequencing, assembly, and annotation of *Acorus gramineus* pseudochromosomes genome (version 1.0).

The genome size of *Acorus gramineus* was predicted to be 400.2 Mb by k-mer analysis[1] (Supplementary Fig. 1 and Supplementary Table 1), which was congruent with the estimation of ~391 Mb using flow cytometry[2, 3]. Here, we report a high-quality, chromosome-level genome assembly using ~47.6 Gb of Nanopore long reads, ~261.2 Gb of MGI-SEQ short-reads and 138G Hi-C data (692,804,666 read pairs) (Supplementary Data 1). The final assembly had a total length of 375.3 Mb (N50: 11.9 Mb) spread across 135 scaffolds. Hi-C assembly anchored them into 69 super-scaffolds[4], with a N50 reached 36.1 Mb (Supplementary Table 2). 98.6% super-scaffolds were placed in one of the 12 pseudochromosomes, with a total length of 370.2 Mb. The number of groups corresponded to the number of chromosomes determined experimentally in somatic cells (2n = 24). The assembled genome was evaluated after Hi-C assembly using BUSCO[5], resulting in 94.8% (Supplementary Table 3) of the core eukaryote genes recovered for the majority of the genome assemblies.

Using a combination of reference plant protein homology support and transcriptome sequencing generated from multiple tissues (flowers, leaves and roots) and *ab initio* gene prediction, 19,778 protein-coding gene models were annotated using the MAKER pipeline[6]. The resulting protein models were then compared with protein sequences in five protein databases, SwissProt[7], KEGG[8], COG, TrEMBL[7], InterPro[9], with 99.52% of genes being assigned putative functional annotations. The assembled draft genome of *A. gramineus* contained 50.06% repetitive sequences. Long terminal repeat (LTR) retrotransposons were the most prevalent type of transposable elements (TE) among these repeats, representing nearly 37.95% of the genome, including 27.52% LTR/*Gypsy* and 7.18% LTR/*Copia* retro-elements.

Supplementary Method 1. Genome assembly and assessment of the assembly quality.

The raw long reads obtained from ONT were *de novo* assembled using the NextDenovo assembler (v2.2, https://github.com/Nextomics/NextDenovo) with the parameters: read_ cutoff = 1k, seed_ cutoff = 25211, and by using NextPolish (v1.3.0, https://github.com/Nextomics/NextPolish)[10], six rounds (two rounds with ONT long reads and four rounds with short reads of polishing were applied to the initial draft assembled contigs. Due to the presence of heterozygosity, it is possible that the contigs representing the two haplotypes of a given region of the genome might be assembled as two independently primary contigs, rather than as a single contig and an associated haplotig. Here, purge_dups (v1.2.3) was used to select which contigs to retain for the haploid assembly by taking into account mapped read coverage using short read and Minimap2 alignments[11]. Following that, contigs were blasted using blast (v2.2.29)[12], against the NCBI non-redundant bacterial database to rule out any possible bacterial contamination. Any contigs for which at least 80% of the total length was aligned and that showed an identity larger than 90% were discarded.

The assembly evaluations for the *A. gramineus* genomes are provided as follows: First, mapping of the conserved core eukaryotic genes from the BUSCO data set (embryophyta_odb10, BUSCO v5.3.2)[5], of the core eukaryote genes recovered for the majority of the genome assemblies; Second, BWA (v.2.21)[13] was used to map the reads to the draft assemblies to evaluate the DNA reads mapping rate (>96.32 %) (Supplementary Data 1). Finally, we mapped the RNA reads to the draft assemblies to evaluate the RNA reads mapping rate using Hisat2[14] (Supplementary Data 1). Taken together, these results indicated good genome assembly qualities for this newly sequenced species.

Supplementary Method 2. Chromosome assignment using Hi-C.

Hi-C paired-end reads were trimmed to remove low-quality bases and adapter sequences using Trimmomatic (v0.40)[15], and all the filtered reads were aligned to contig using Juicer (https://github.com/aidenlab/juicer, v3)[16] to calculate the contact frequency. Then, 3D-DNA (v180922)[17] with two iterative rounds for misjoin correction (-r 2) and other parameters set to the default values. The oriented scaffolds were used to build the interaction matrices with Juicer, and to make manual corrections with Juicebox assembly tools (v1.11.08)[18].

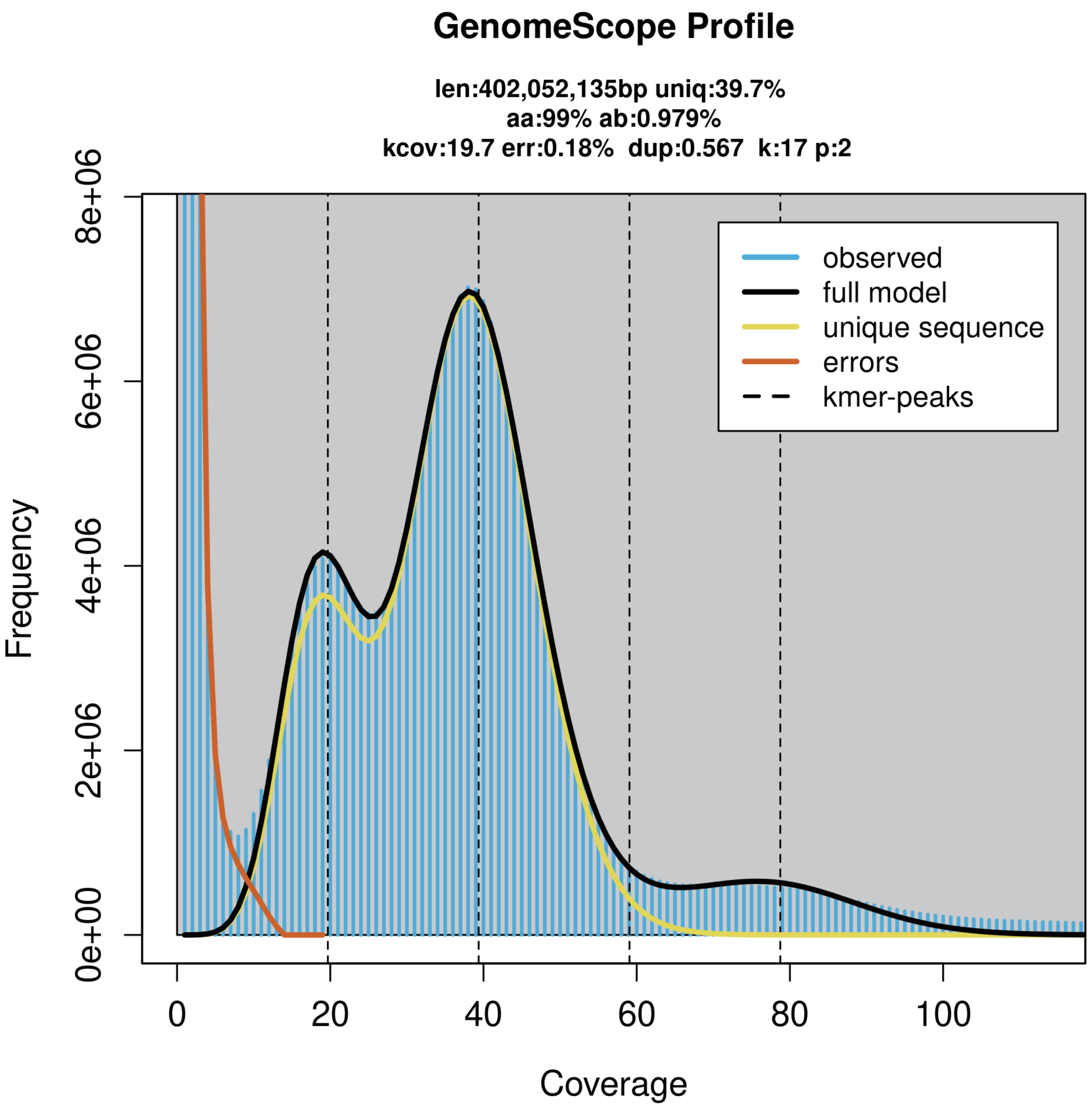

Supplementary Figure 1. K-mer analysis of *Acorus gramineus* with GenomeScope2.0 (k=17). The x-axis refers to the k-mer coverage, the y-axis refers to the frequency of the k-mer for a given coverage.

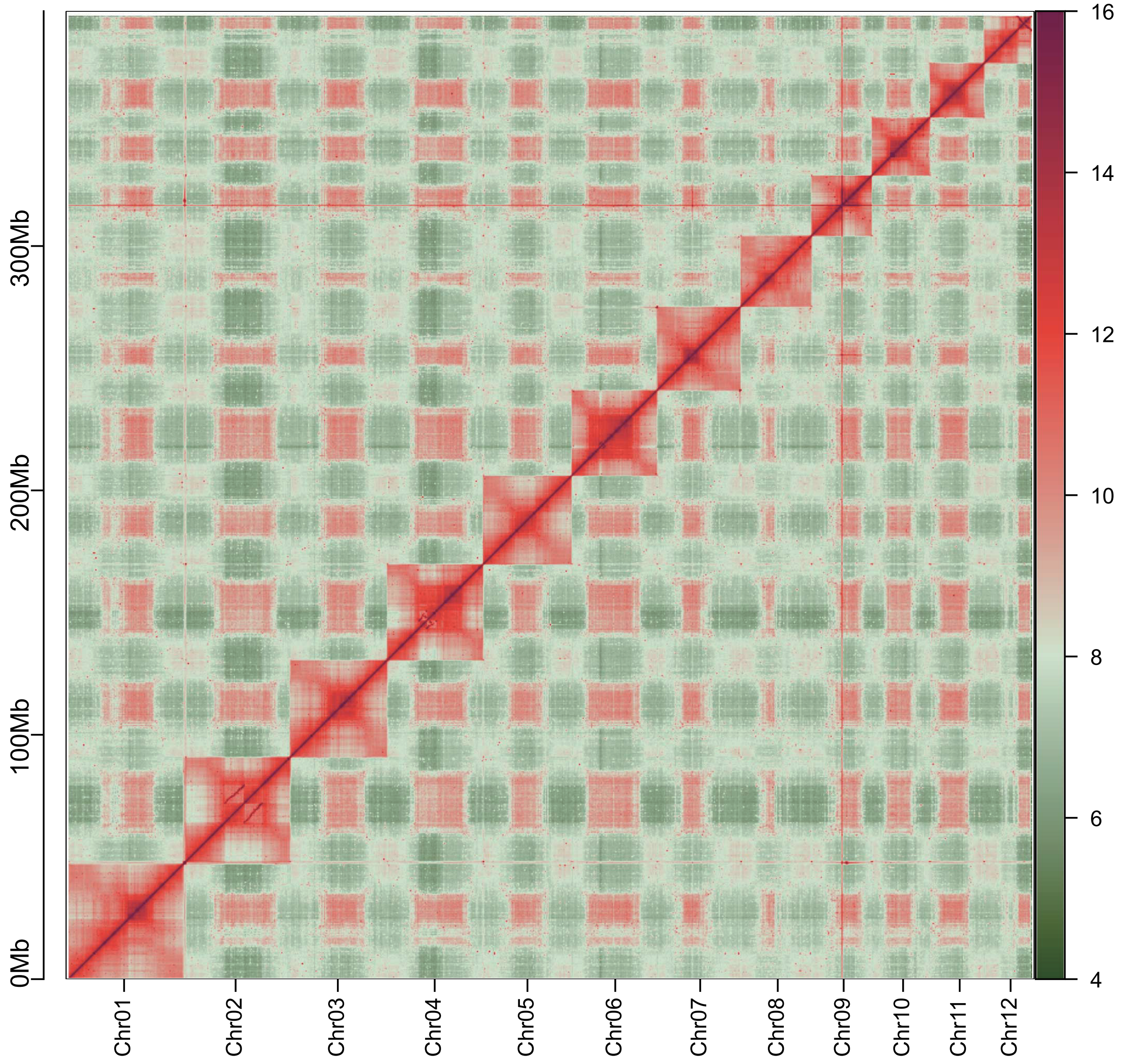

Supplementary Figure 2. Hi-C plot of the psudochromosomes level assembly *Acorus gramineus* genome. The x-axis refers to the pseudo-chromosomes number, the y-axis refers to the genome size, the number on the right refers to the range of colour.

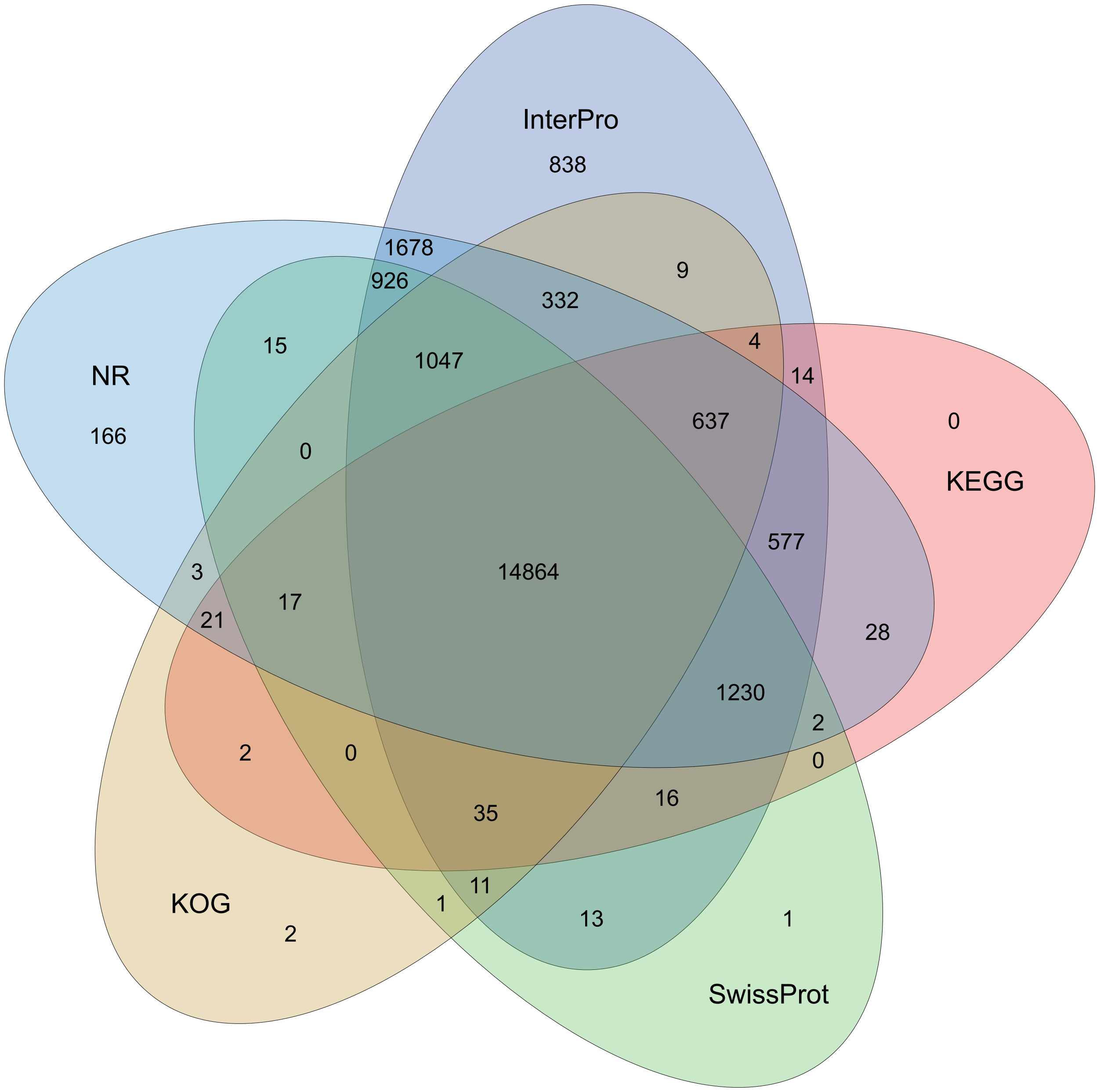

Supplementary Figure 3. Venn diagram of the number of genes with functional annotation using multiple public databases.

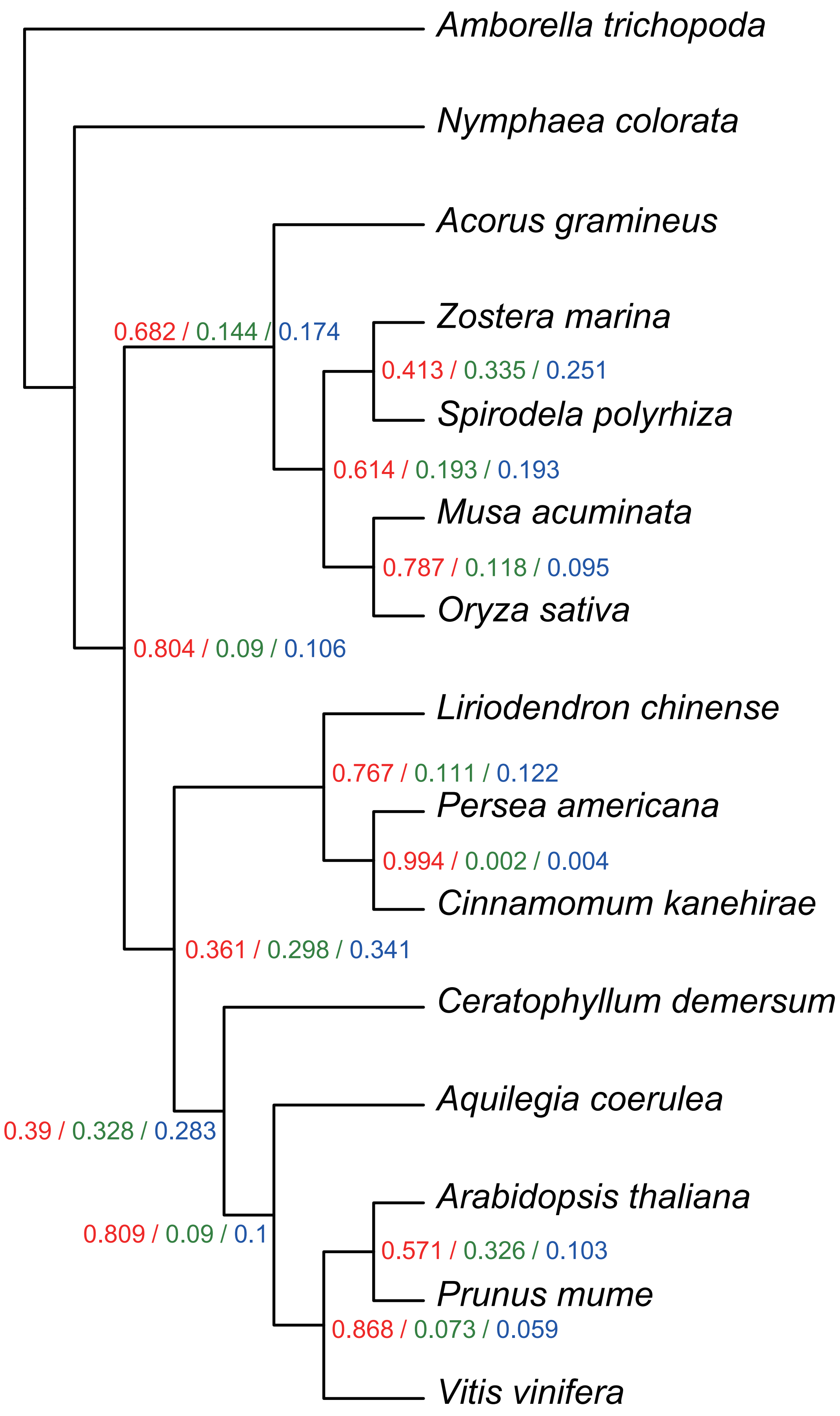

Supplementary Figure 4. The phylogenetic tree constructed by ASTRAL based on the dataset of 633 single-copy ortholog sets. the numbers in turn shows quartet score of the main topology(RL|SO,current tree) and two others alternative topologies (RS|LO and RO|LS). Source data underlying Supplementary Figure 4 are provided as a Source Data file.

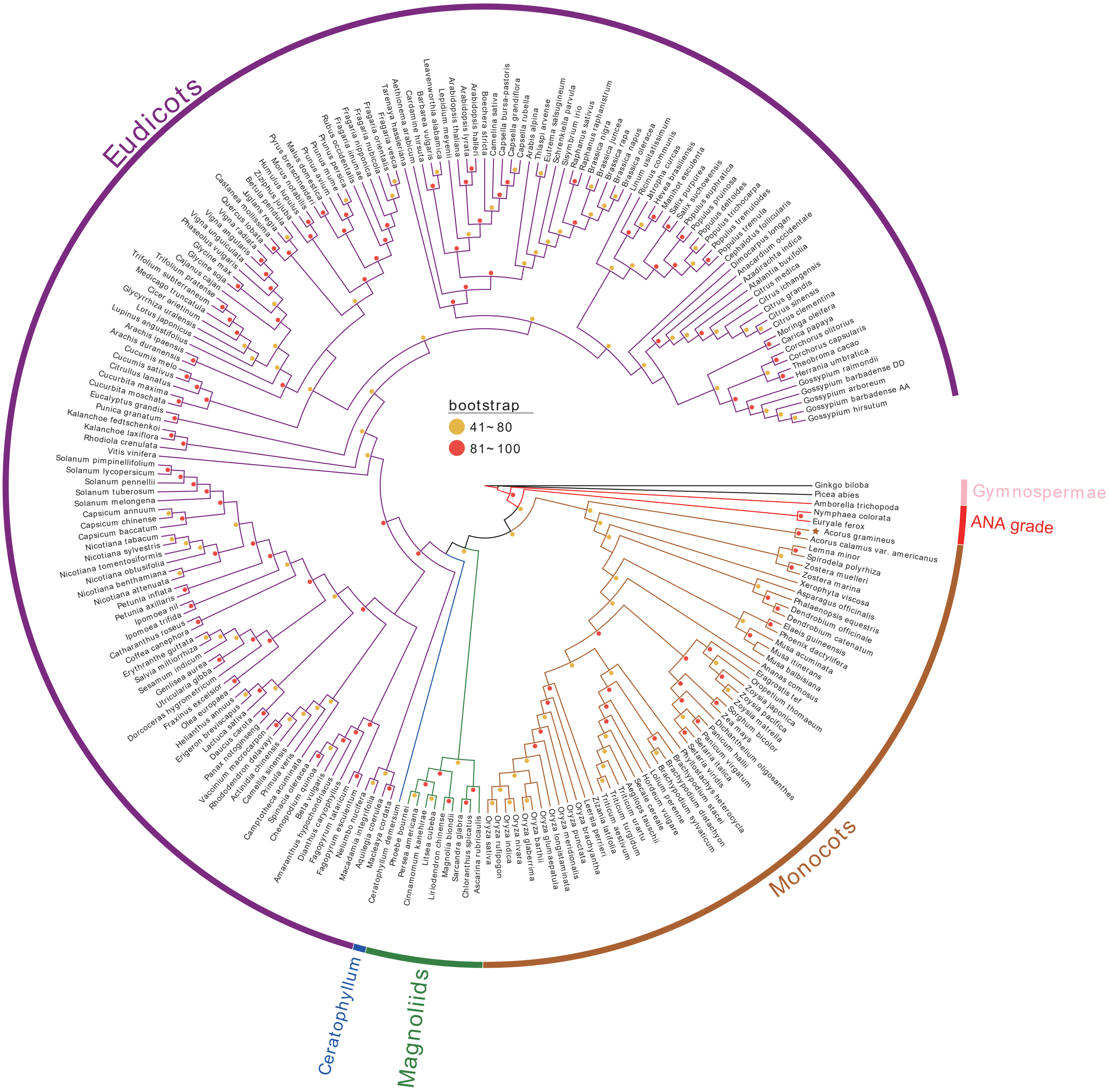

Supplementary Figure 5. The phylogenetic tree constructed by IQTREE based on a concatenated dataset of 612 low-copy orthologous genes from 223 species. Source data underlying Supplementary Figure 5 are provided as a Source Data file.

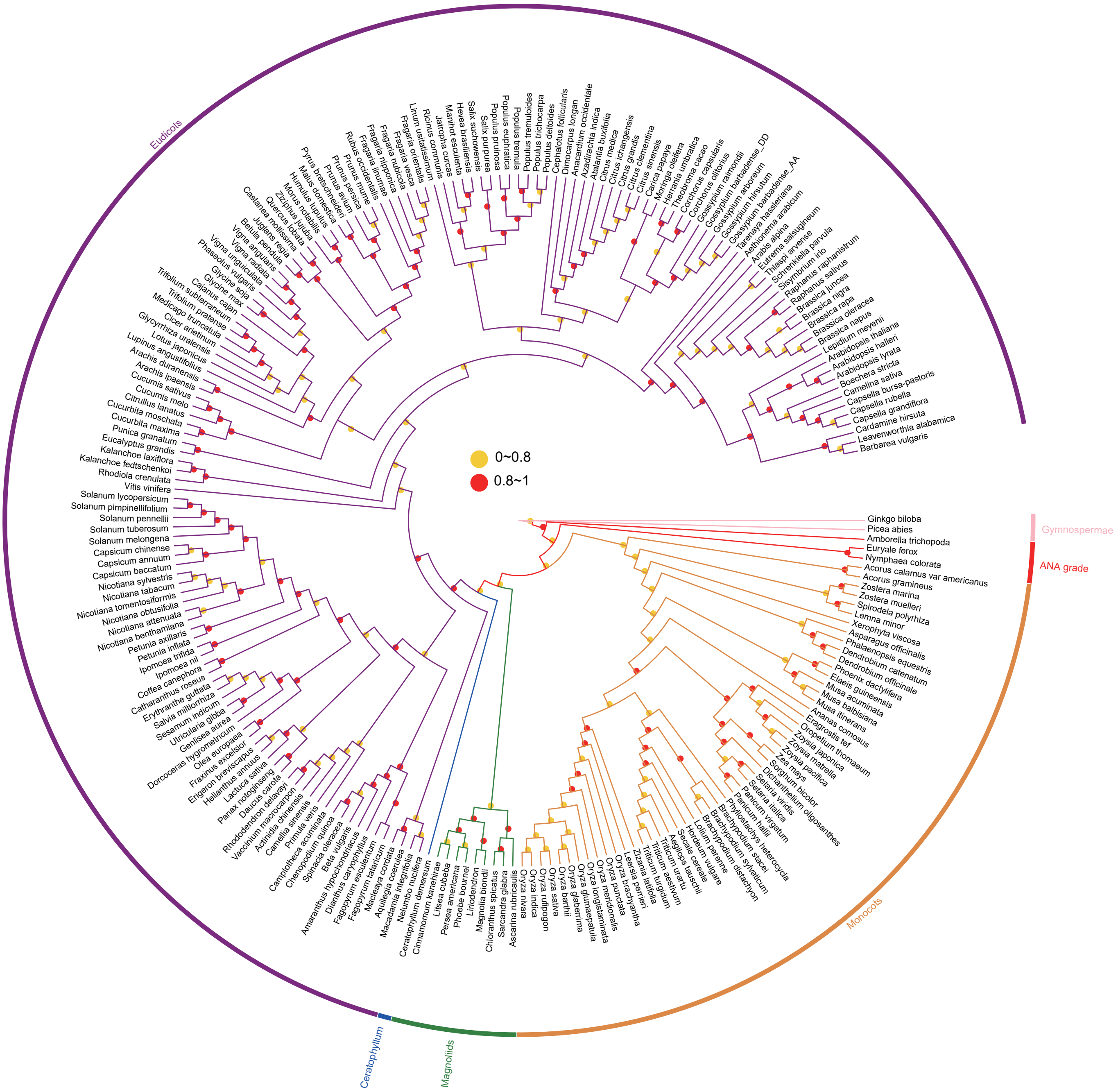

Supplementary Figure 6. The phylogenetic tree constructed by ASTRAL based on the dataset of 612 low-copy orthologous genes from 223 species. the numbers shows quartet score of the main topology(RL|SO,current tree). Source data underlying Supplementary Figure 6 are provided as a Source Data file.

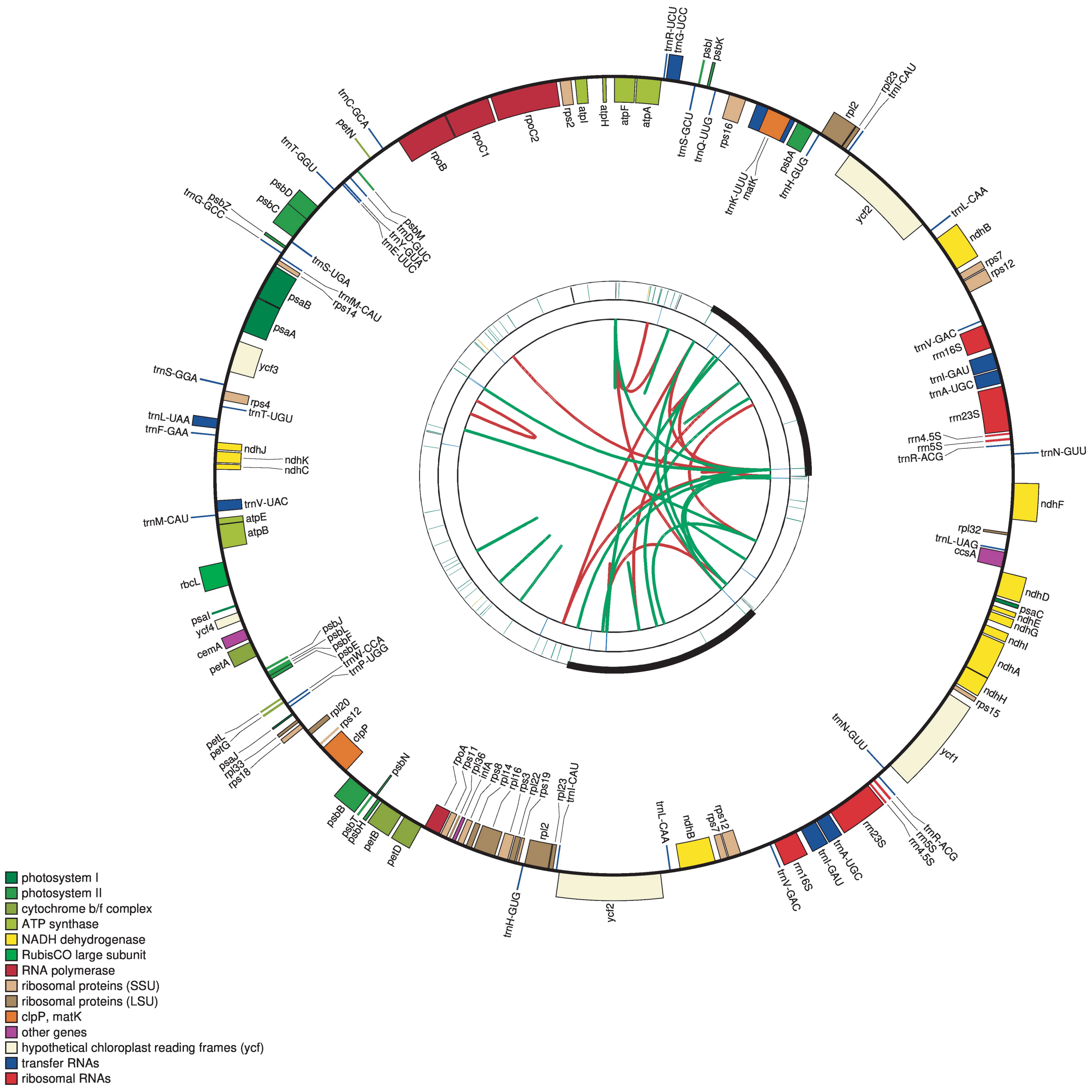

Supplementary Figure 7. Gene map of the *Acorus gramineus* chloroplast genome. From the center going outward, the first circle shows the forward and reverse repeats connected with red and green arcs respectively. The next circle shows the tandem repeats marked with short bars. The third circle shows the microsatellite sequences identified using MISA. The fourth circle is drawn using draw gene map and shows the gene structure on the plastome. The genes were colored based on their functional categories.

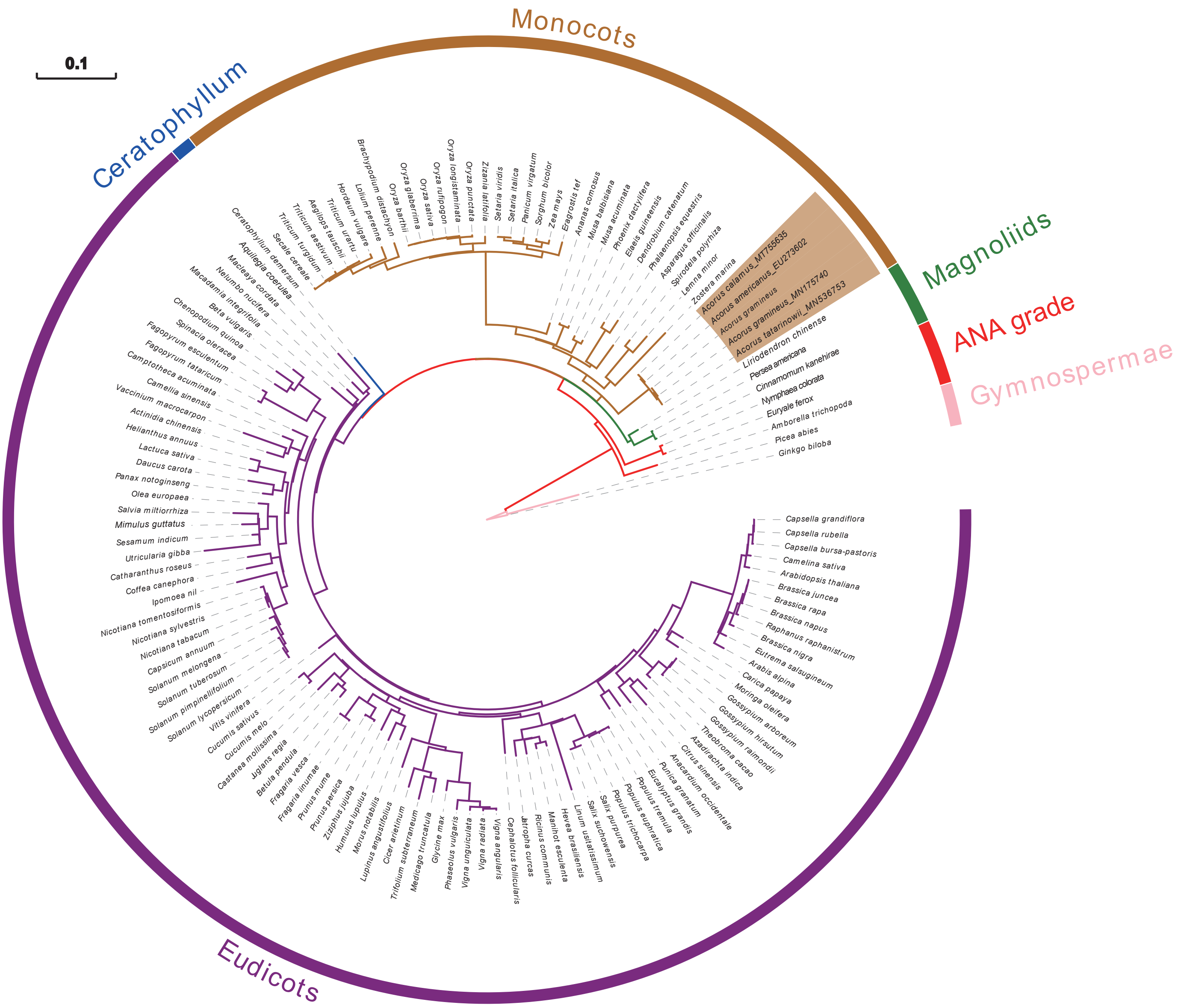

Supplementary Figure 8. Phylogenetic tree based on the 80 chloroplast gene sequences of 135 plant species. Source data underlying Supplementary Figure 8 are provided as a Source Data file.

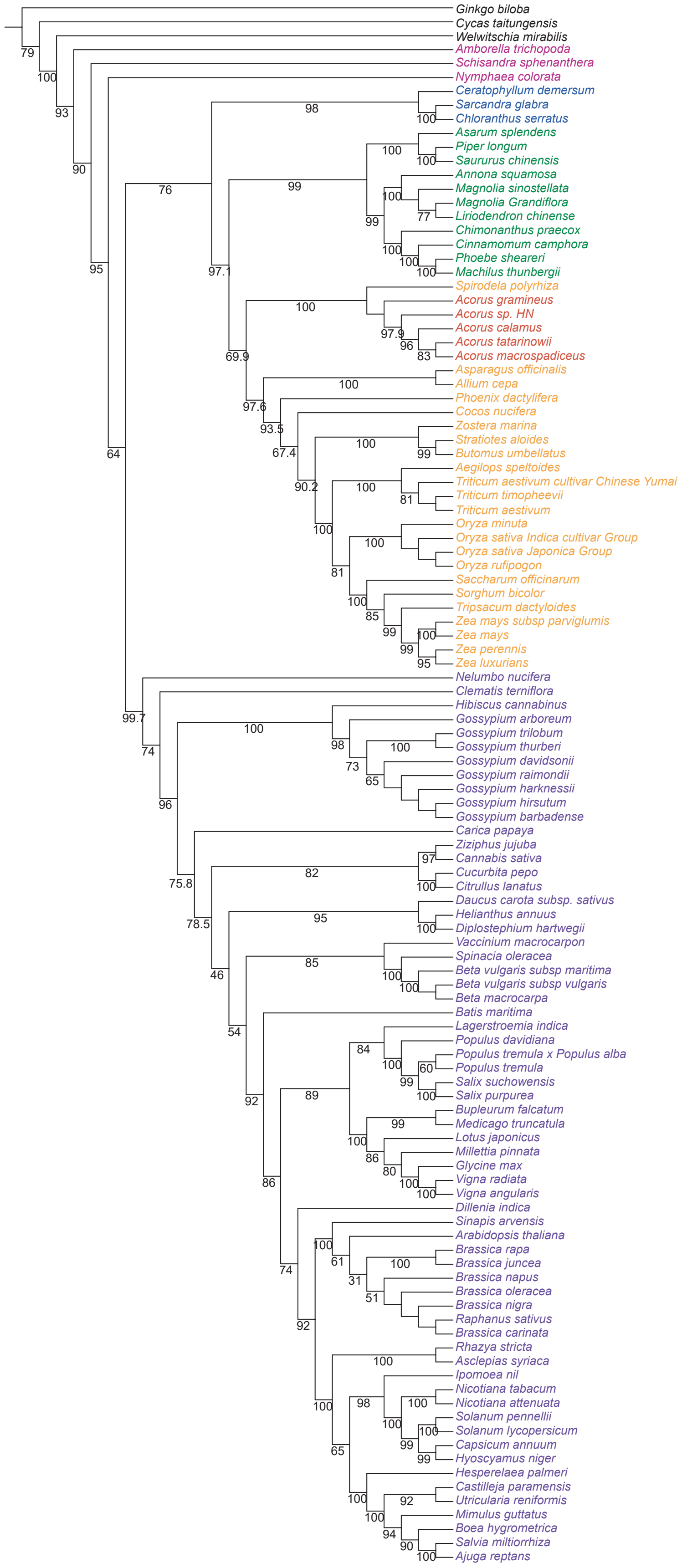

Supplementary Figure 9. The tree of mitochondrial gene *atp1* in *Acorus gramineus* and land plants. ANA groups are marked with pink, Magnoliids are marked with green, Mononcots are marked with yellow, Eudicots are marked with purple, Chloranthales and Ceratophyllales are marked with blue, and *Acorus* are marked with red. Source data underlying Supplementary Figure 9 are provided as a Source Data file.

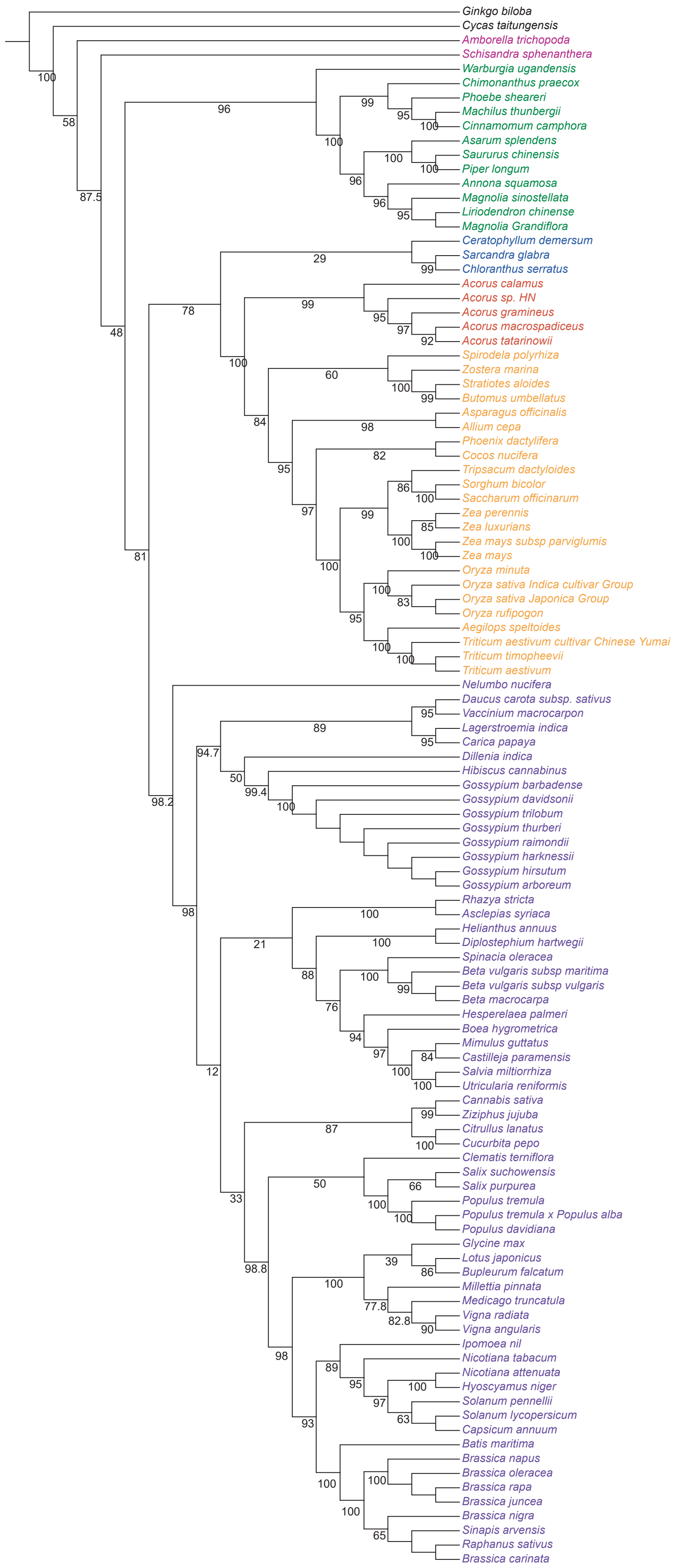

Supplementary Figure 10. The tree of mitochondrial gene *atp4* in *Acorus gramineus* and land plants. ANA groups are marked with pink, Magnoliids are marked with green, Mononcots are marked with yellow, Eudicots are marked with purple, Chloranthales and Ceratophyllales are marked with blue, and *Acorus* are marked with red. Source data underlying Supplementary Figure 10 are provided as a Source Data file.

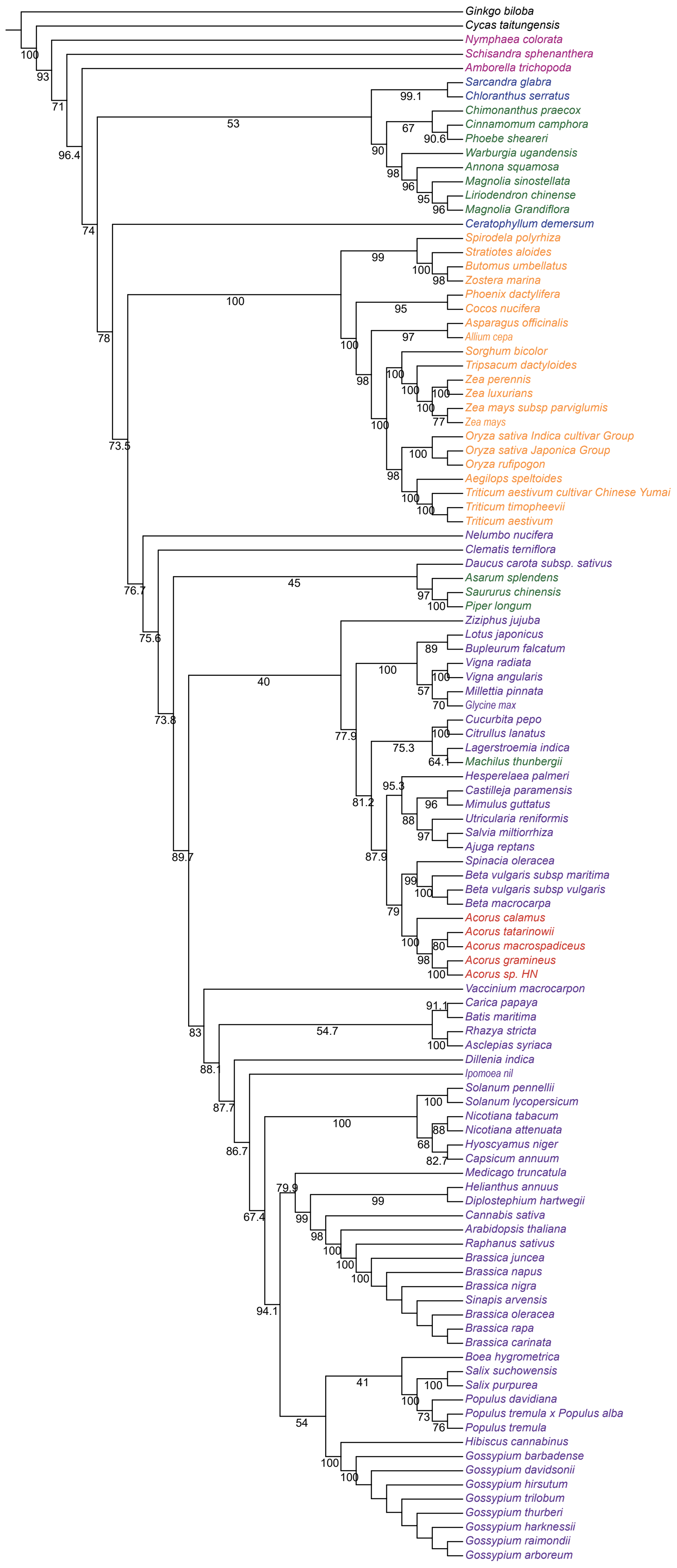

Supplementary Figure 11. The tree of mitochondrial gene *atp6* in *Acorus gramineus* and land plants. ANA groups are marked with pink, Magnoliids are marked with green, Mononcots are marked with yellow, Eudicots are marked with purple, Chloranthales and Ceratophyllales are marked with blue, and *Acorus* are marked with red. Source data underlying Supplementary Figure 11 are provided as a Source Data file.

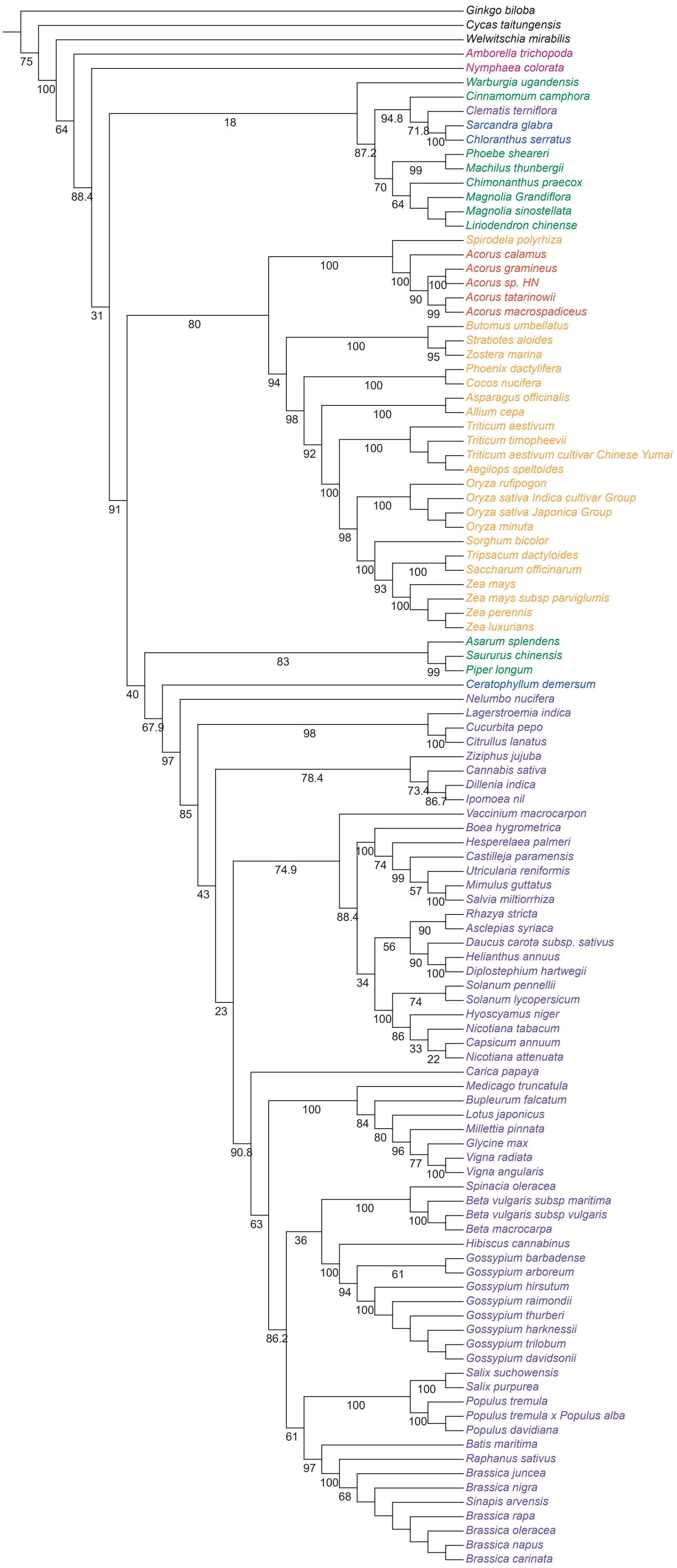

Supplementary Figure 12. The tree of mitochondrial gene *atp8* in *Acorus gramineus* and land plants. ANA groups are marked with pink, Magnoliids are marked with green, Mononcots are marked with yellow, Eudicots are marked with purple, Chloranthales and Ceratophyllales are marked with blue, and Acorus are marked with red. Source data underlying Supplementary Figure 12 are provided as a Source Data file.

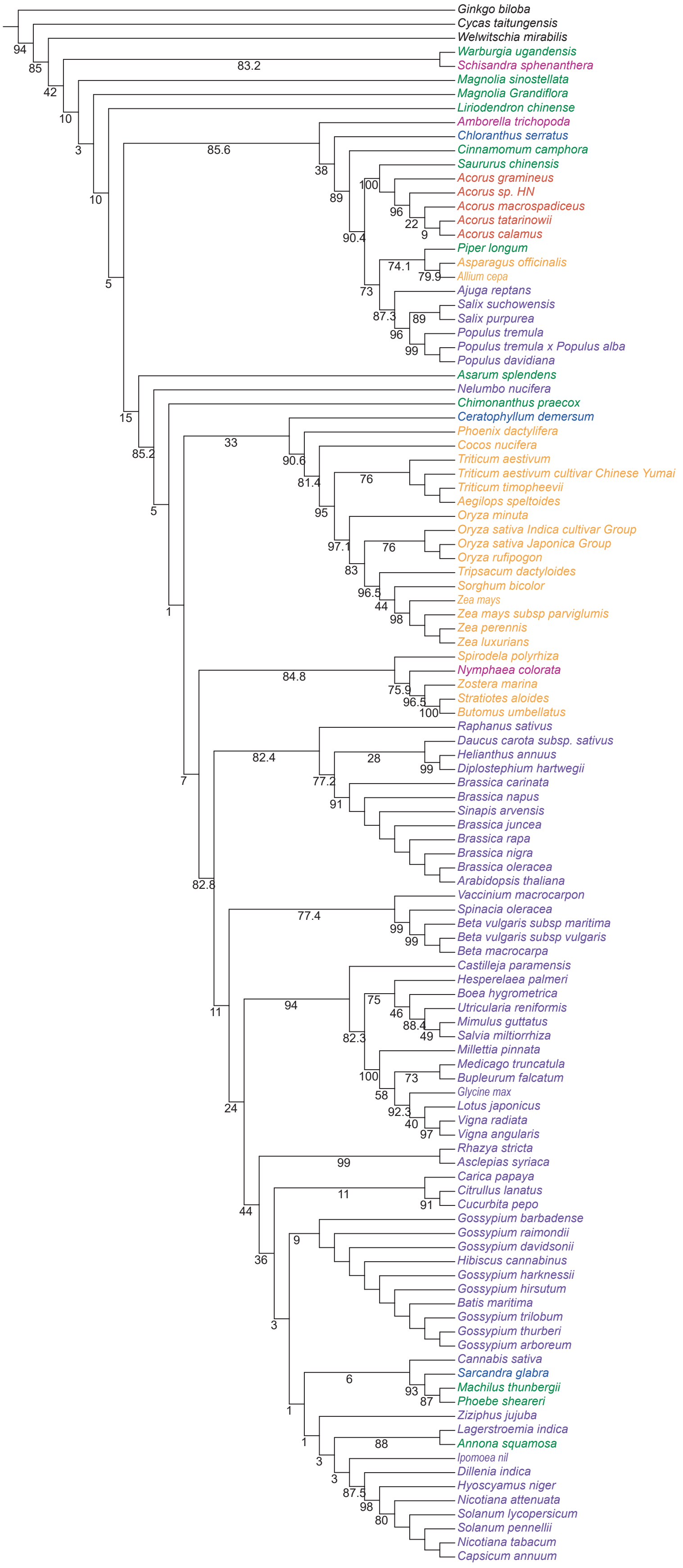

Supplementary Figure 13. The tree of mitochondrial gene *atp9* in *Acorus gramineus* and land plants. ANA groups are marked with pink, Magnoliids are marked with green, Mononcots are marked with yellow, Eudicots are marked with purple, Chloranthales and Ceratophyllales are marked with blue, and *Acorus* are marked with red. Source data underlying Supplementary Figure 13 are provided as a Source Data file.

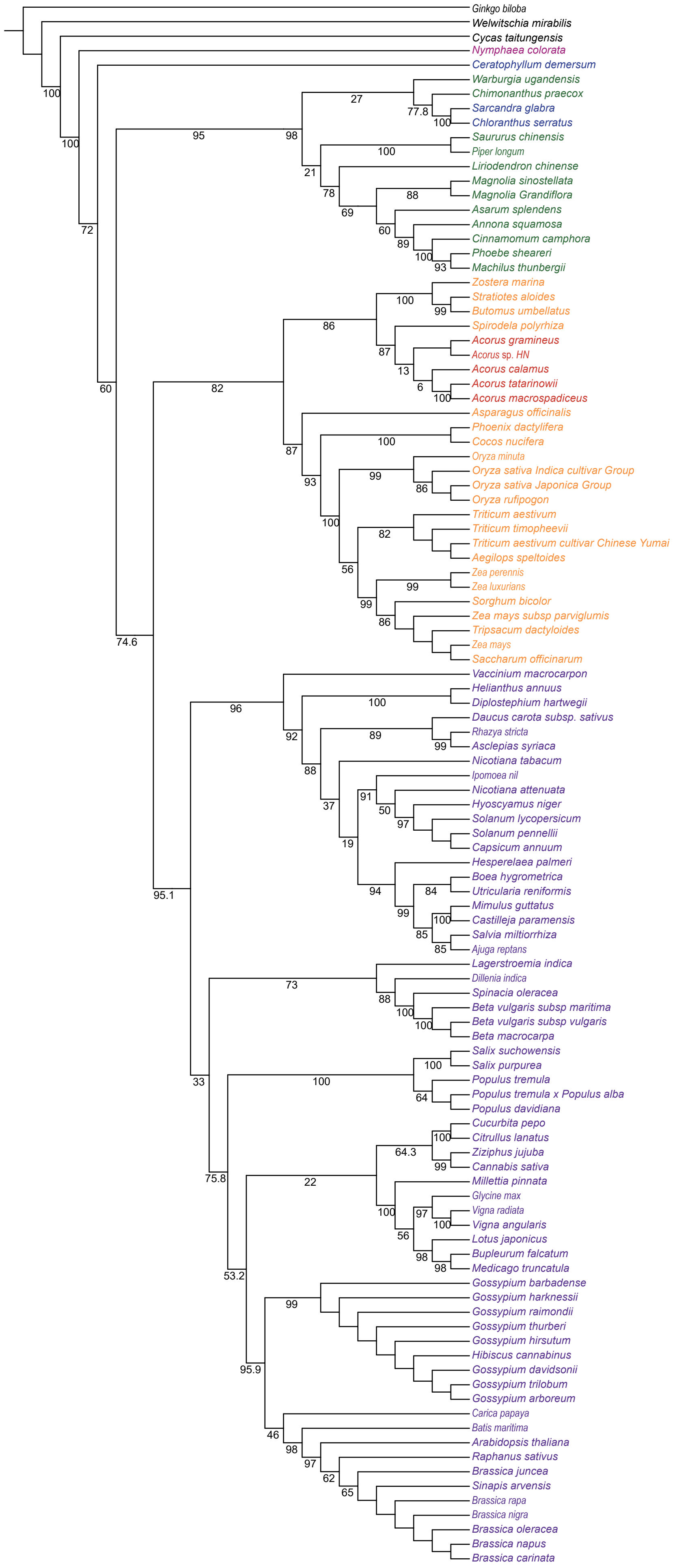

Supplementary Figure 14. The tree of mitochondrial gene *ccmB* in *Acorus gramineus* and land plants. ANA groups are marked with pink, Magnoliids are marked with green, Mononcots are marked with yellow, Eudicots are marked with purple, Chloranthales and Ceratophyllales are marked with blue, and *Acorus* are marked with red. Source data underlying Supplementary Figure 14 are provided as a Source Data file.

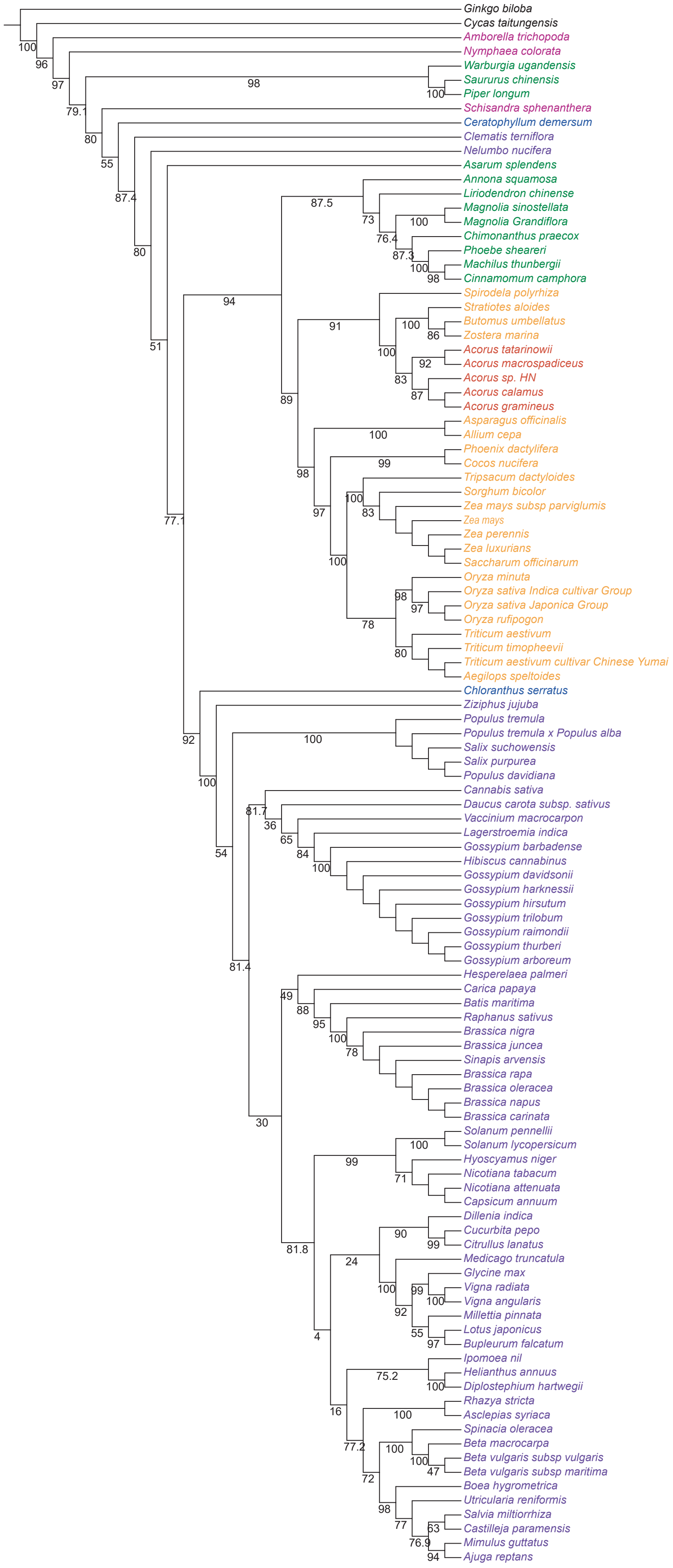

Supplementary Figure 15. The tree of mitochondrial gene *ccmC* in *Acorus gramineus* and land plants. ANA groups are marked with pink, Magnoliids are marked with green, Mononcots are marked with yellow, Eudicots are marked with purple, Chloranthales and Ceratophyllales are marked with blue, and *Acorus* are marked with red. Source data underlying Supplementary Figure 15 are provided as a Source Data file.

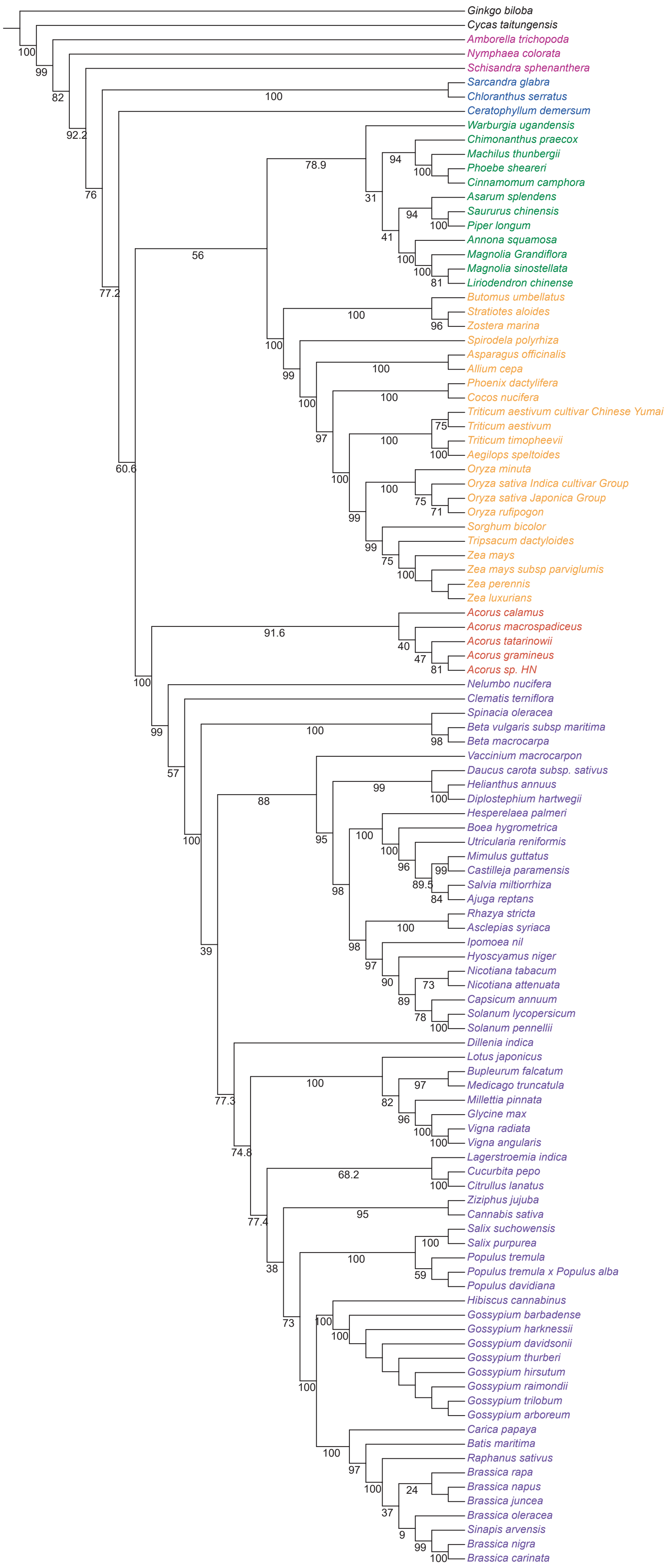

Supplementary Figure 16. The tree of mitochondrial gene *ccmFC* in *Acorus gramineus* and land plants. ANA groups are marked with pink, Magnoliids are marked with green, Mononcots are marked with yellow, Eudicots are marked with purple, Chloranthales and Ceratophyllales are marked with blue, and *Acorus* are marked with red. Source data underlying Supplementary Figure 16 are provided as a Source Data file.

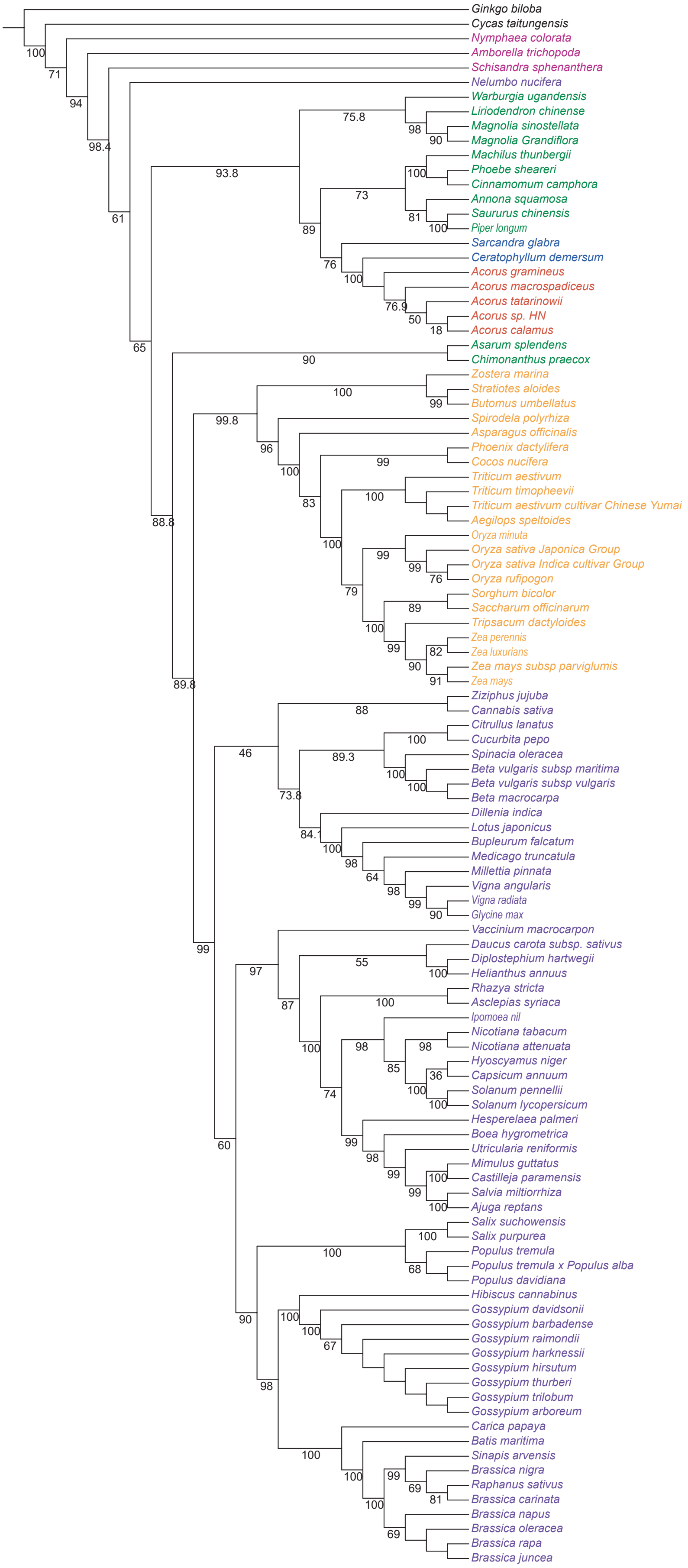

Supplementary Figure 17. The tree of mitochondrial gene *ccmFN* in *Acorus gramineus* and land plants. ANA groups are marked with pink, Magnoliids are marked with green, Mononcots are marked with yellow, Eudicots are marked with purppe, Chloranthales and Ceratophyllales are marked with blue, and *Acorus* are marked with red. Source data underlying Supplementary Figure 17 are provided as a Source Data file.

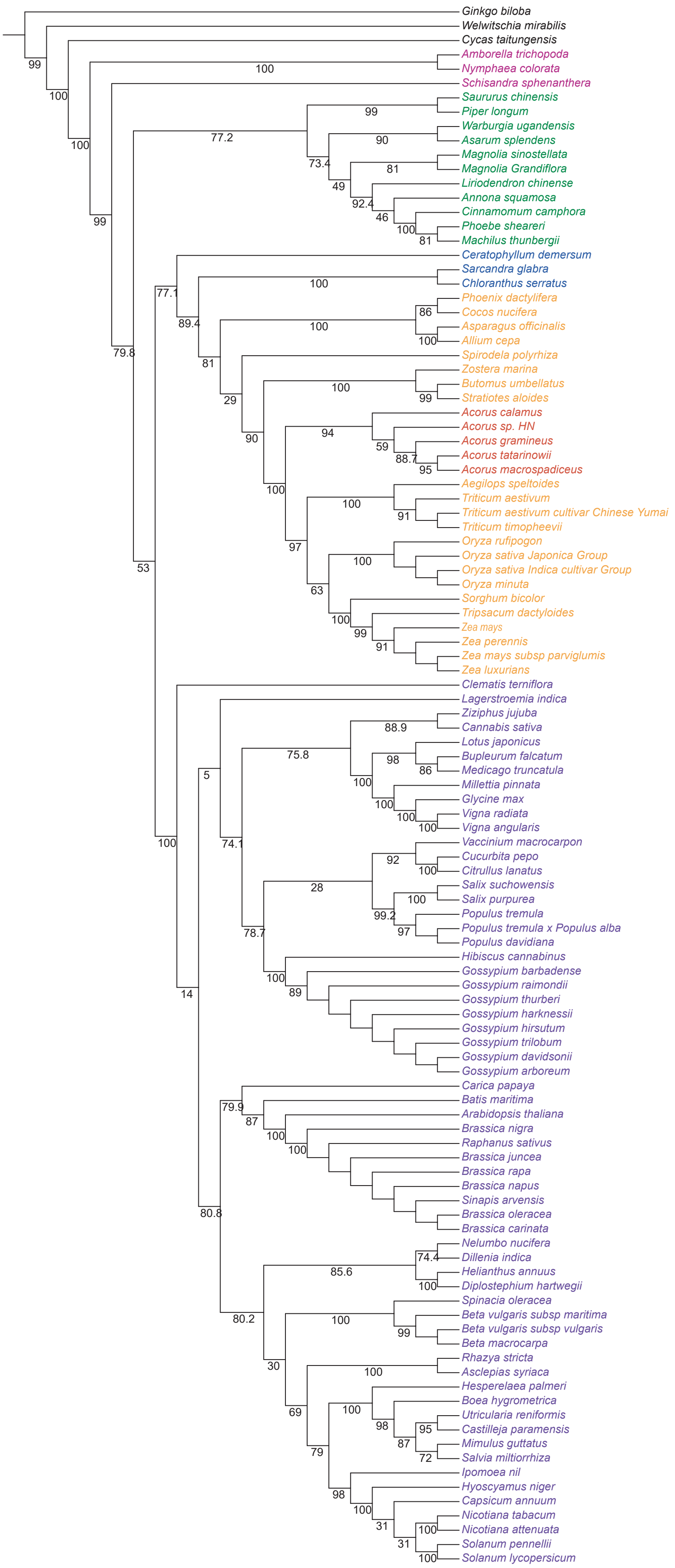

Supplementary Figure 18. The tree of mitochondrial gene *cob* in *Acorus gramineus* and land plants. ANA groups are marked with pink, Magnoliids are marked with green, Mononcots are marked with yellow, Eudicots are marked with purple, Chloranthales and Ceratophyllales are marked with blue, and *Acorus* are marked with red. Source data underlying Supplementary Figure 18 are provided as a Source Data file.

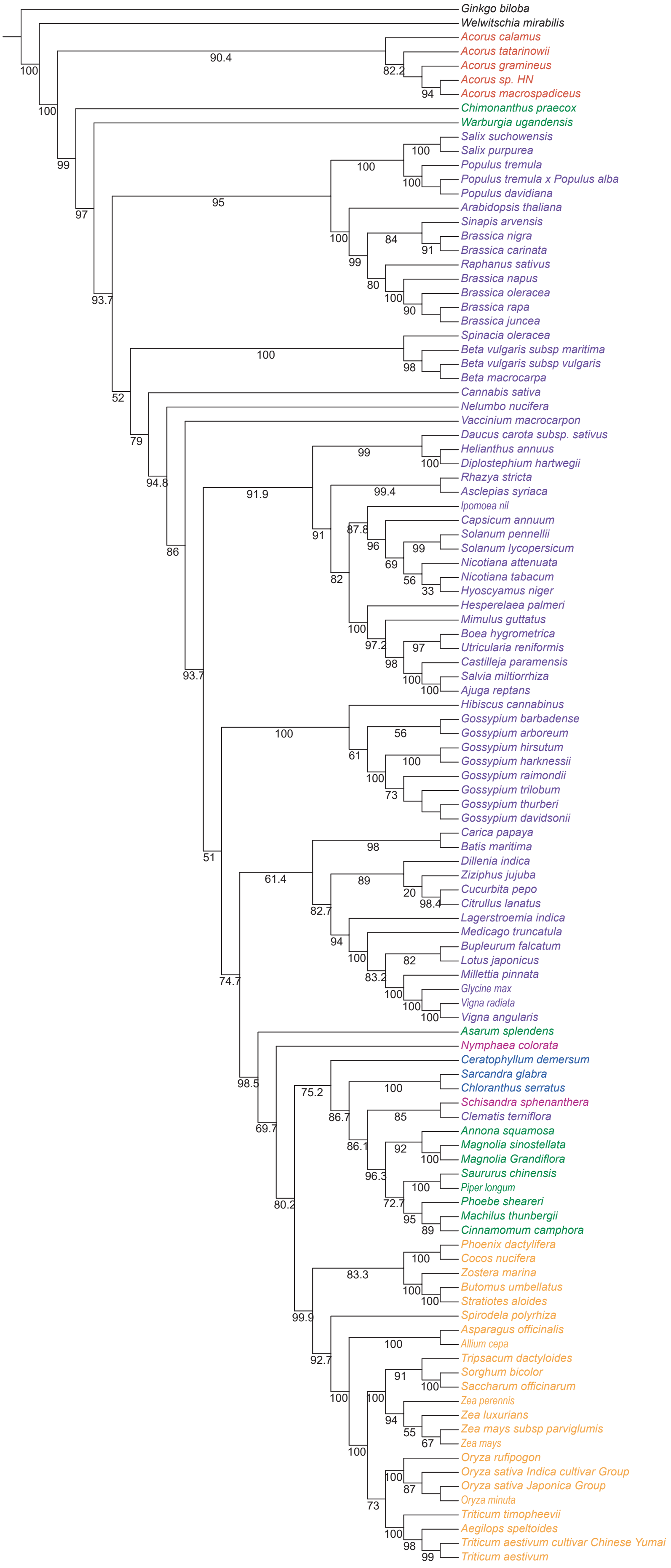

Supplementary Figure 19. The tree of mitochondrial gene *cox1* in *Acorus gramineus* and land plants. ANA groups are marked with pink, Magnoliids are marked with green, Mononcots are marked with yellow, Eudicots are marked with purple, Chloranthales and Ceratophyllales are marked with blue, and *Acorus* are marked with red. Source data underlying Supplementary Figure 19 are provided as a Source Data file.

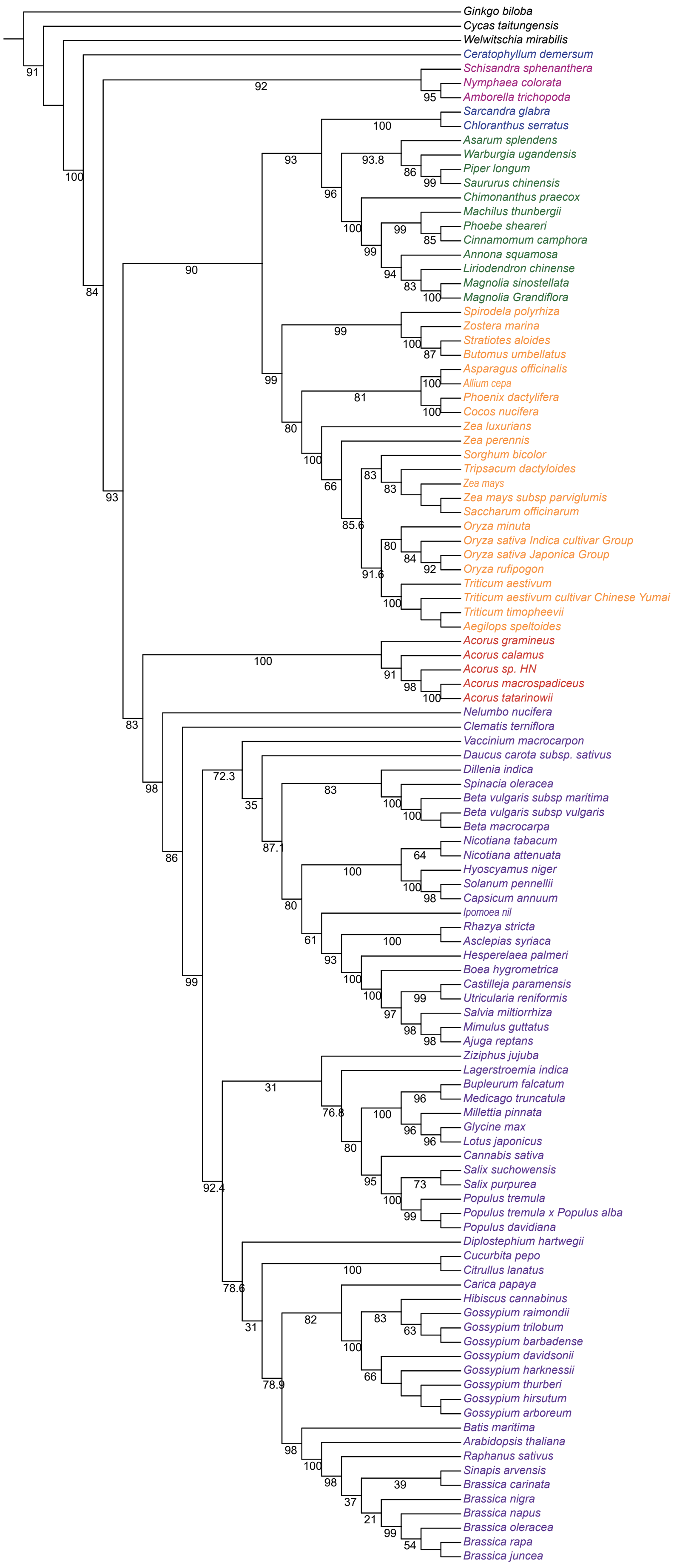

Supplementary Figure 20. The tree of mitochondrial gene *cox2* in *Acorus gramineus* and land plants. ANA groups are marked with pink, Magnoliids are marked with green, Mononcots are marked with yellow, Eudicots are marked with purple, Chloranthales and Ceratophyllales are marked with blue, and *Acorus* are marked with red. Source data underlying Supplementary Figure 20 are provided as a Source Data file.

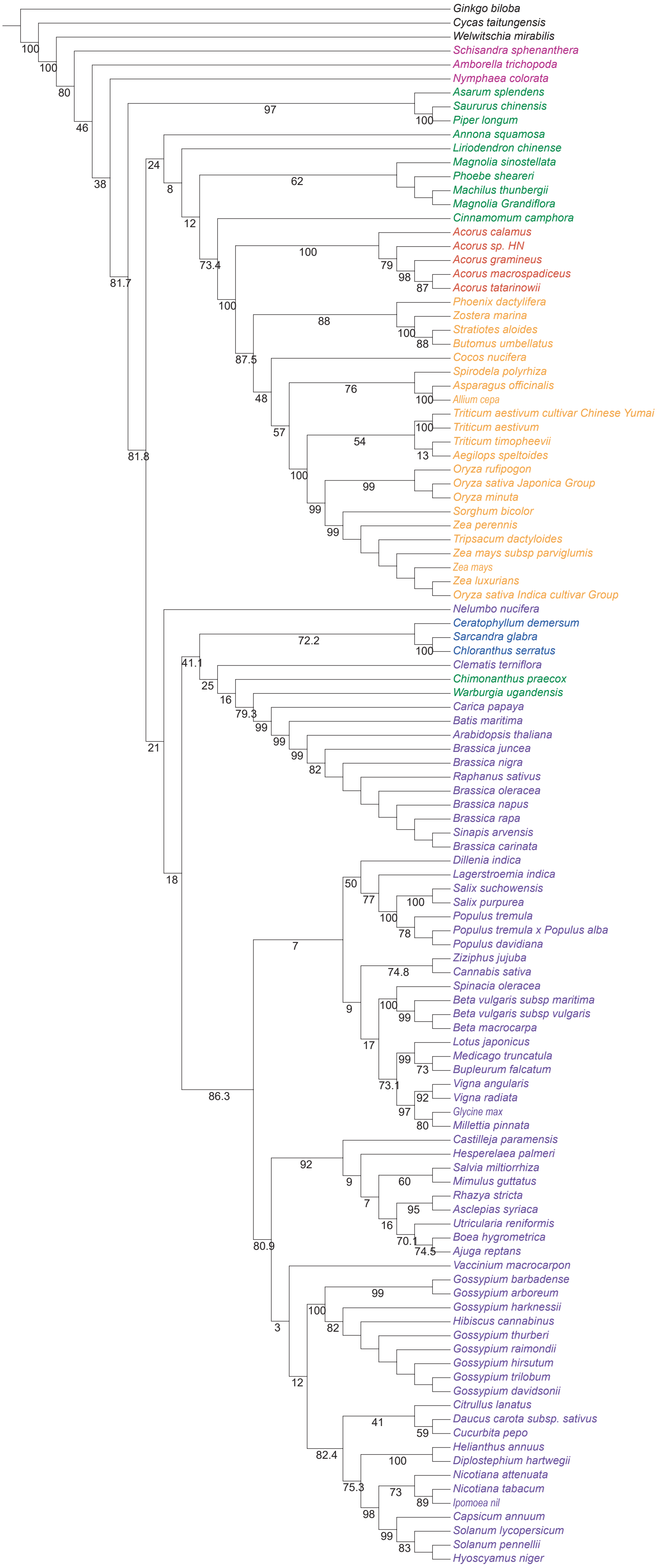

Supplementary Figure 21. The tree of mitochondrial gene *cox3* in *Acorus gramineus* and land plants. ANA groups are marked with pink, Magnoliids are marked with green, Mononcots are marked with yellow, Eudicots are marked with purple, Chloranthales and Ceratophyllales are marked with blue, and *Acorus* are marked with red. Source data underlying Supplementary Figure 21 are provided as a Source Data file.

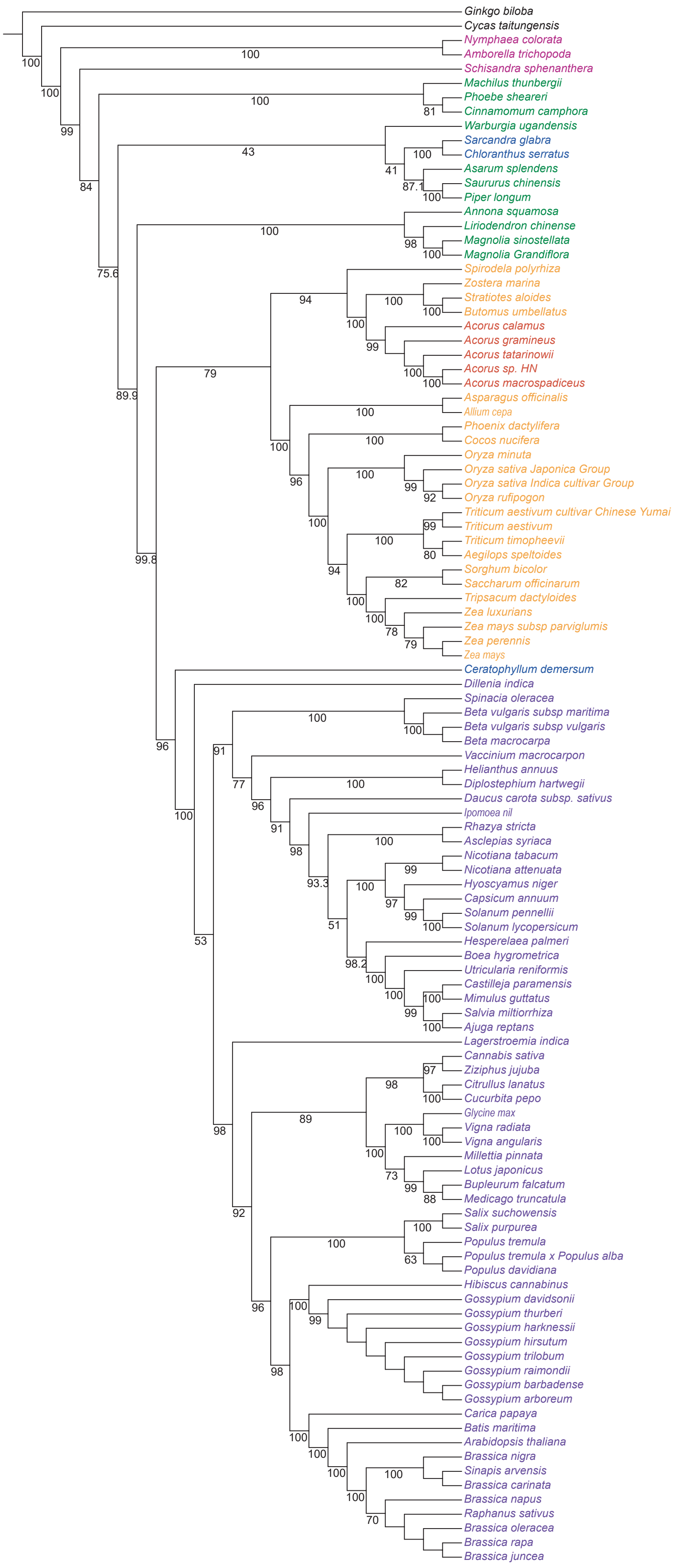

Supplementary Figure 22. The tree of mitochondrial gene *matR* in *Acorus gramineus* and land plants. ANA groups are marked with pink, Magnoliids are marked with green, Mononcots are marked with yellow, Eudicots are marked with purple, Chloranthales and Ceratophyllales are marked with blue, and *Acorus* are marked with red. Source data underlying Supplementary Figure 22 are provided as a Source Data file.

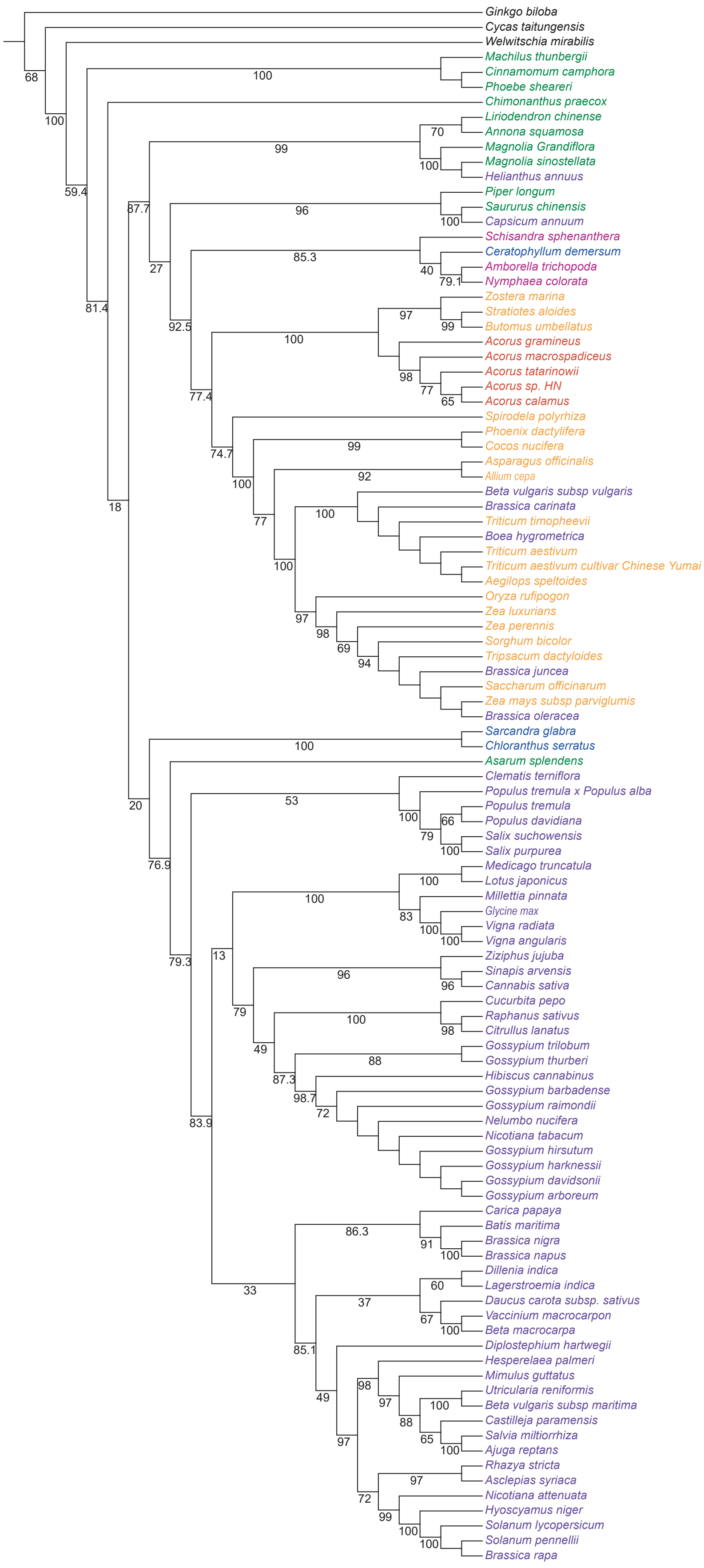

Supplementary Figure 23. The tree of mitochondrial gene *mttB* in *Acorus gramineus* and land plants. ANA groups are marked with pink, Magnoliids are marked with green, Mononcots are marked with yellow, Eudicots are marked with purple, Chloranthales and Ceratophyllales are marked with blue, and *Acorus* are marked with red. Source data underlying Supplementary Figure 23 are provided as a Source Data file.

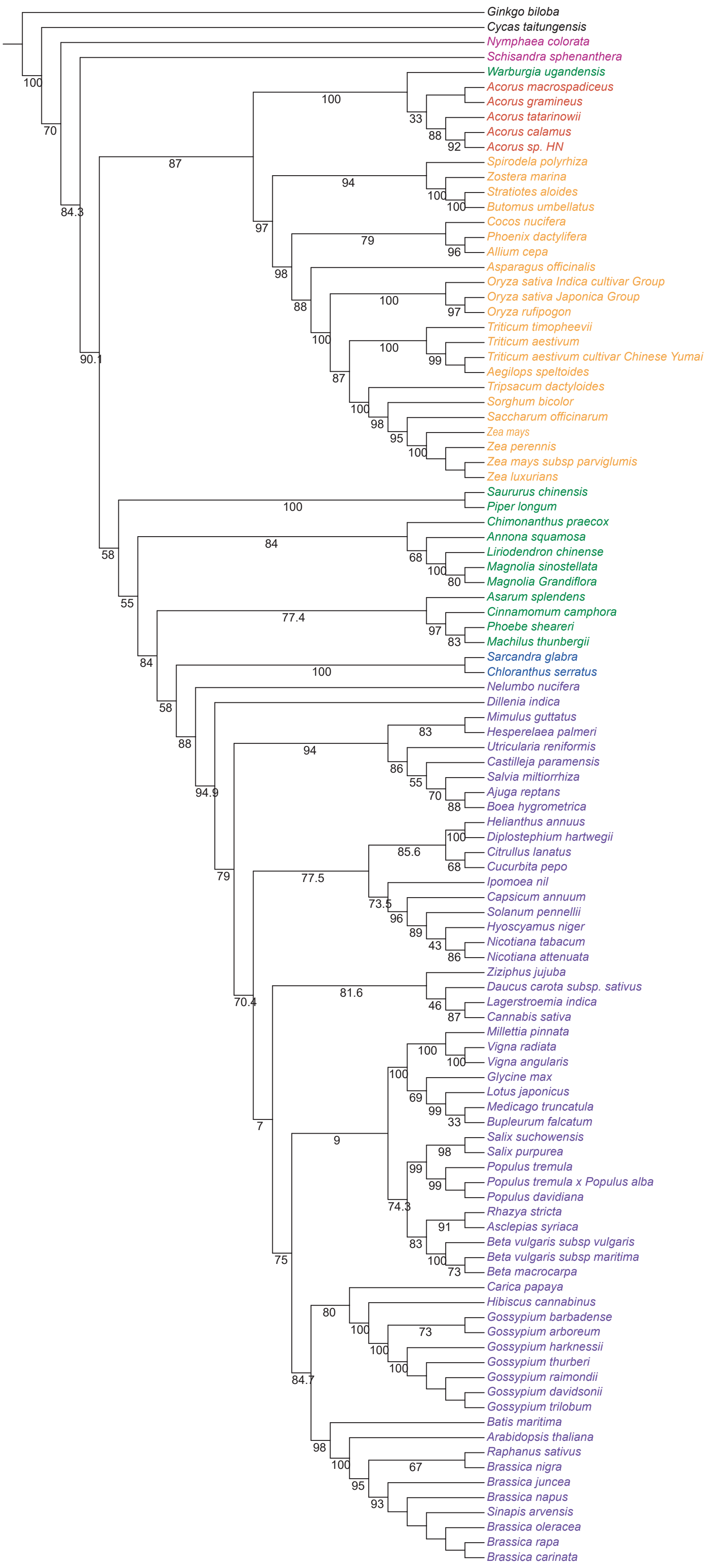

Supplementary Figure 24. The tree of mitochondrial gene *nad1* in *Acorus gramineus* and land plants. ANA groups are marked with pink, Magnoliids are marked with green, Mononcots are marked with yellow, Eudicots are marked with purple, Chloranthales and Ceratophyllales are marked with blue, and *Acorus* are marked with red. Source data underlying Supplementary Figure 24 are provided as a Source Data file.

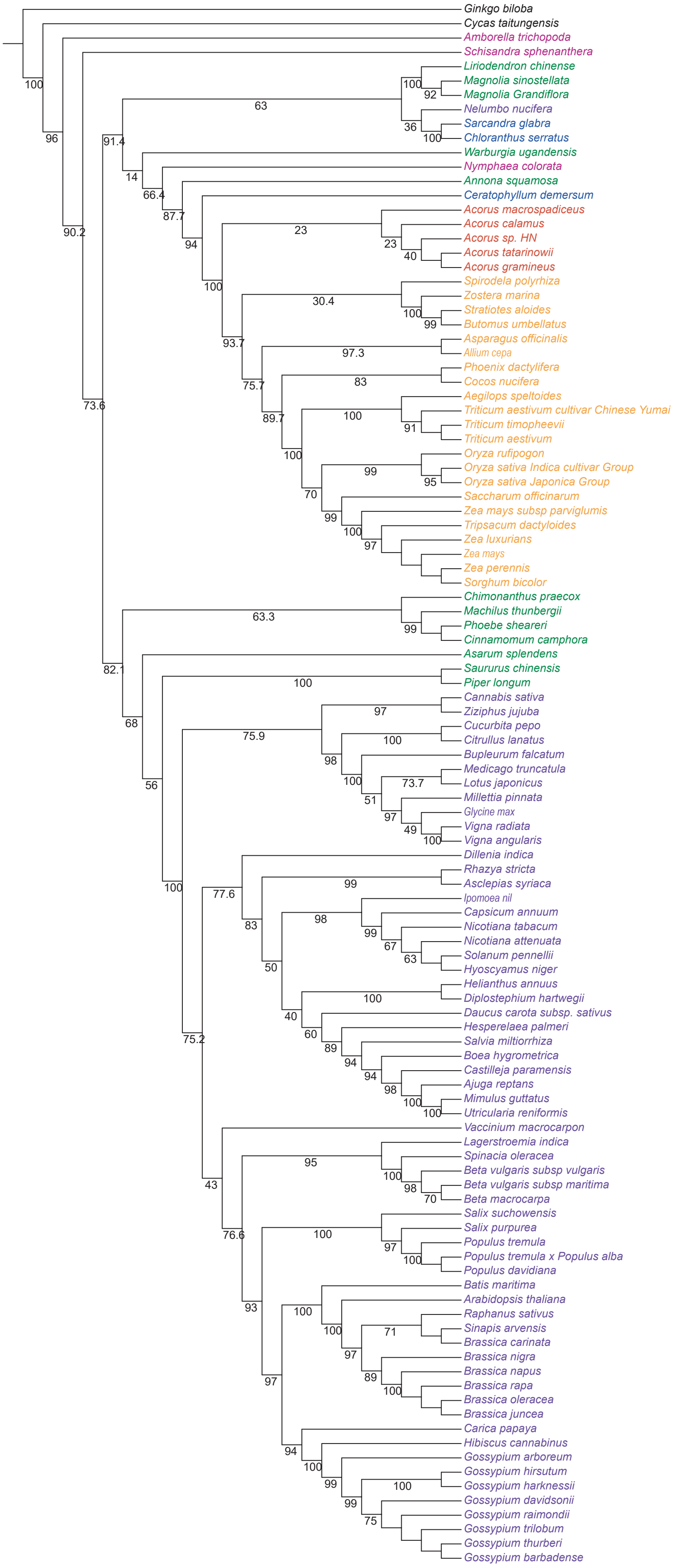

Supplementary Figure 25. The tree of mitochondrial gene *nad2* in *Acorus gramineus* and land plants. ANA groups are marked with pink, Magnoliids are marked with green, Mononcots are marked with yellow, Eudicots are marked with purple, Chloranthales and Ceratophyllales are marked with blue, and *Acorus* are marked with red. Source data underlying Supplementary Figure 25 are provided as a Source Data file.

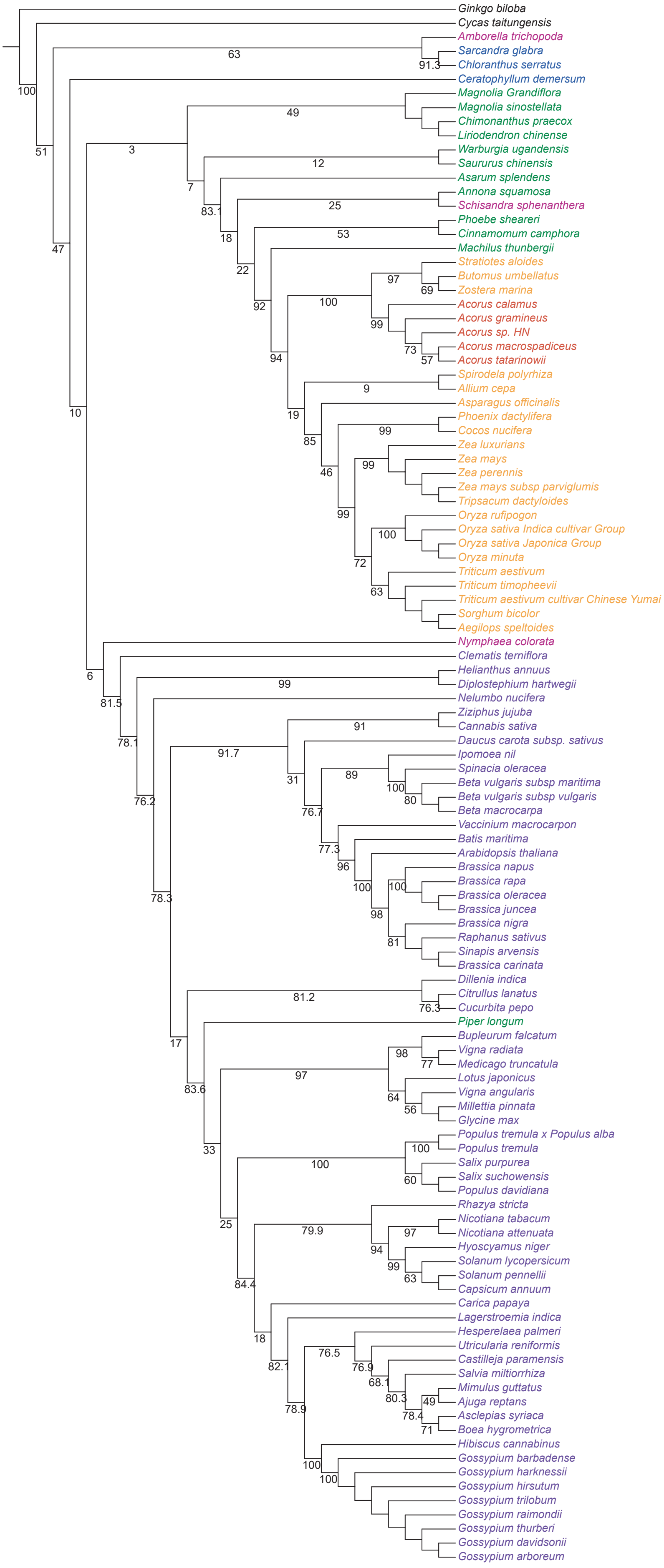

Supplementary Figure 26. The tree of mitochondrial gene *nad3* in *Acorus gramineus* and land plants. ANA groups are marked with pink, Magnoliids are marked with green, Mononcots are marked with yellow, Eudicots are marked with purple, Chloranthales and Ceratophyllales are marked with blue, and *Acorus* are marked with red. Source data underlying Supplementary Figure 26 are provided as a Source Data file.

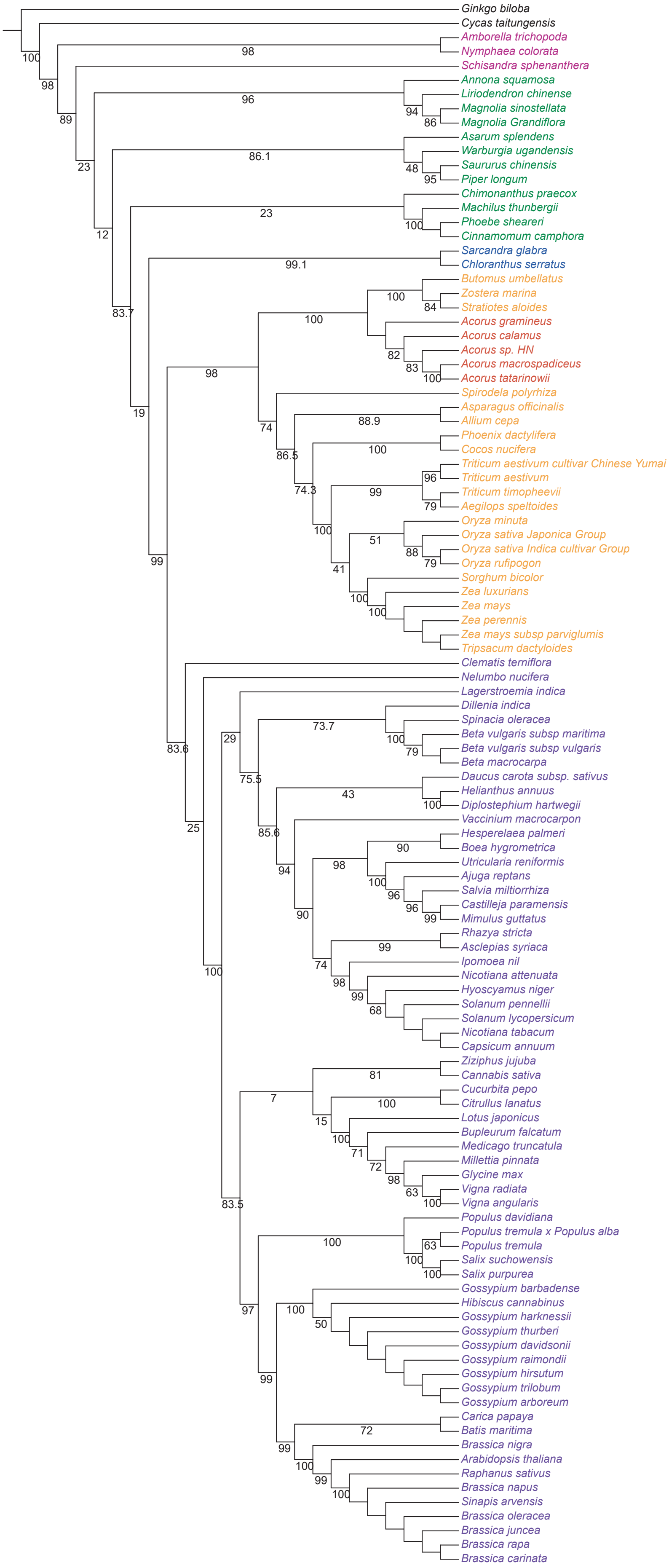

Supplementary Figure 27. The tree of mitochondrial gene *nad4* in *Acorus gramineus* and land plants. ANA groups are marked with pink, Magnoliids are marked with green, Mononcots are marked with yellow, Eudicots are marked with purple, Chloranthales and Ceratophyllales are marked with blue, and *Acorus* are marked with red. Source data underlying Supplementary Figure 27 are provided as a Source Data file.

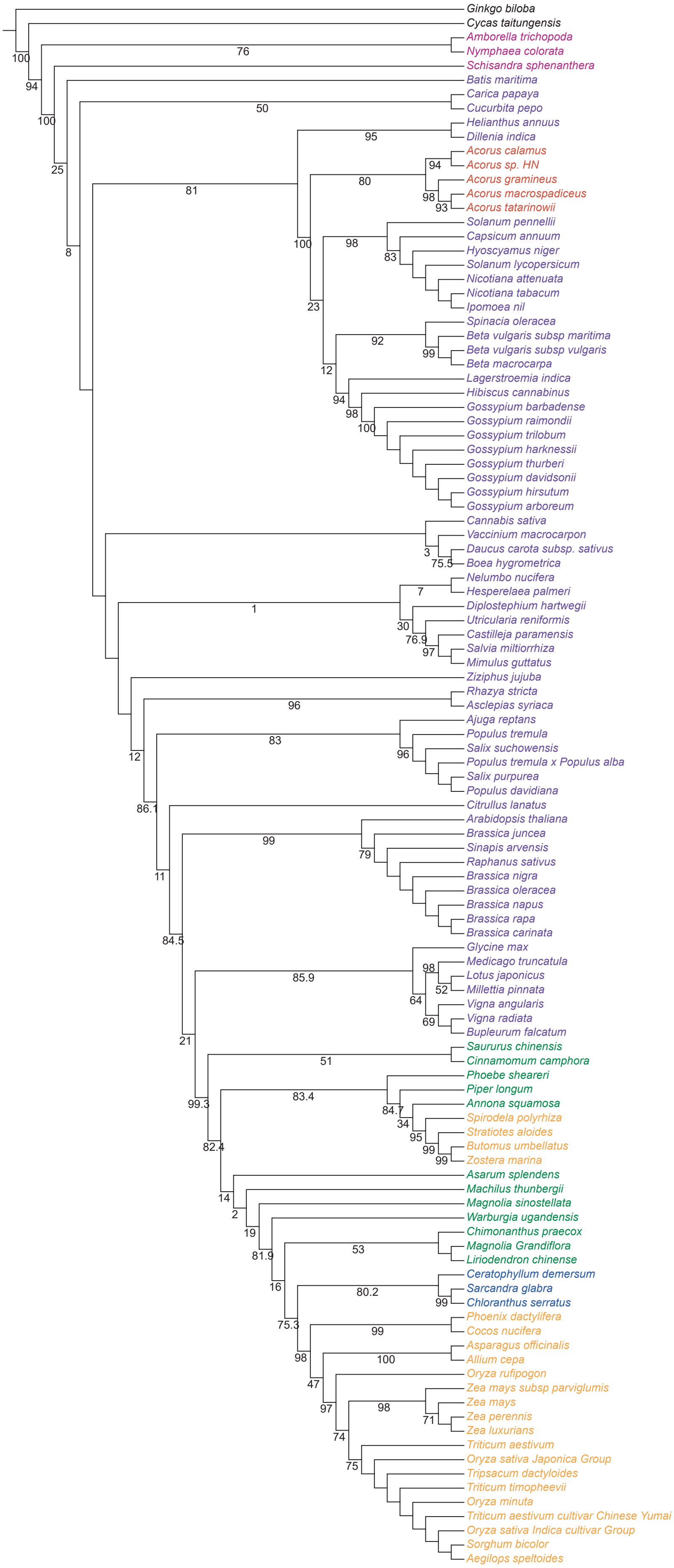

Supplementary Figure 28. The tree of mitochondrial gene *nad4L* in *Acorus gramineus* and land plants. ANA groups are marked with pink, Magnoliids are marked with green, Mononcots are marked with yellow, Eudicots are marked with purple, Chloranthales and Ceratophyllales are marked with blue, and *Acorus* are marked with red. Source data underlying Supplementary Figure 28 are provided as a Source Data file.

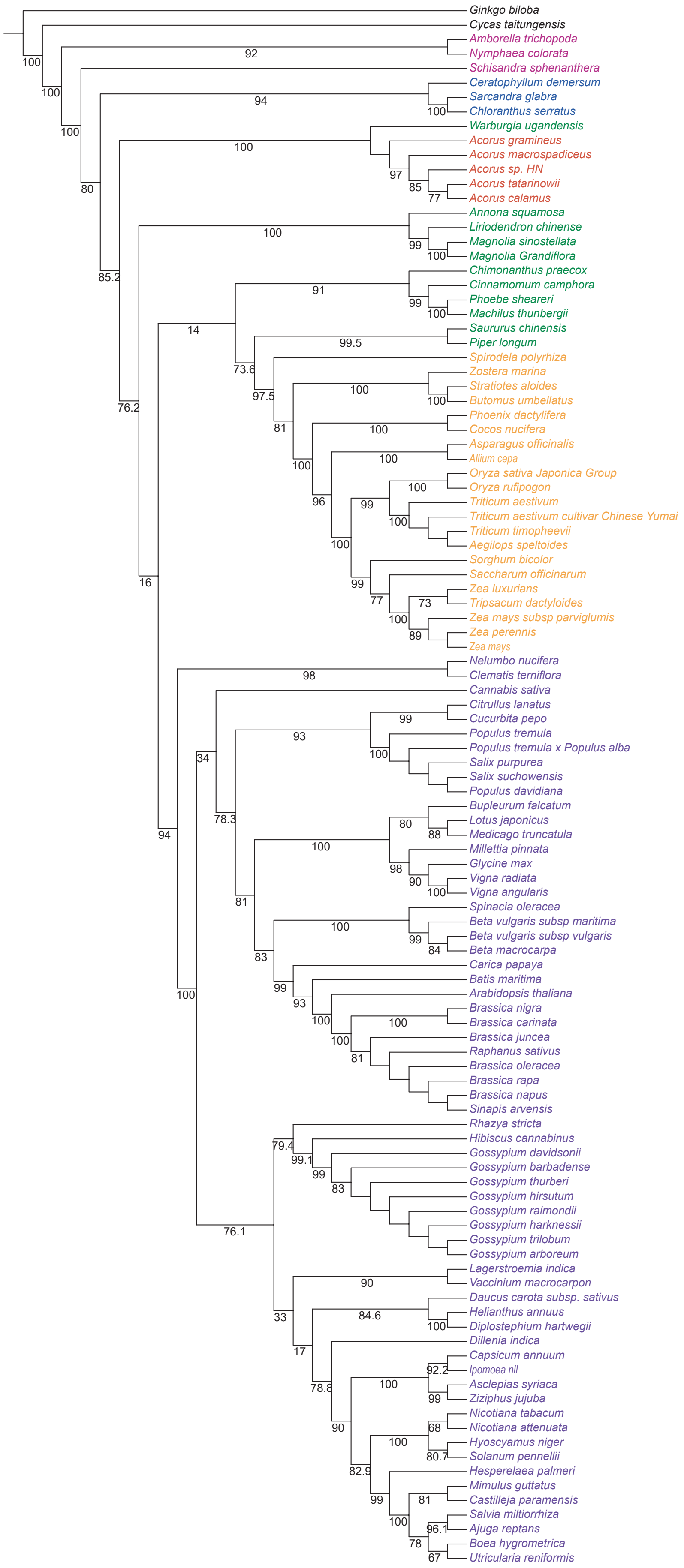

Supplementary Figure 29. The tree of mitochondrial gene *nad5* in *Acorus gramineus* and land plants. ANA groups are marked with pink, Magnoliids are marked with green, Mononcots are marked with yellow, Eudicots are marked with purple, Chloranthales and Ceratophyllales are marked with blue, and *Acorus* are marked with red. Source data underlying Supplementary Figure 29 are provided as a Source Data file.

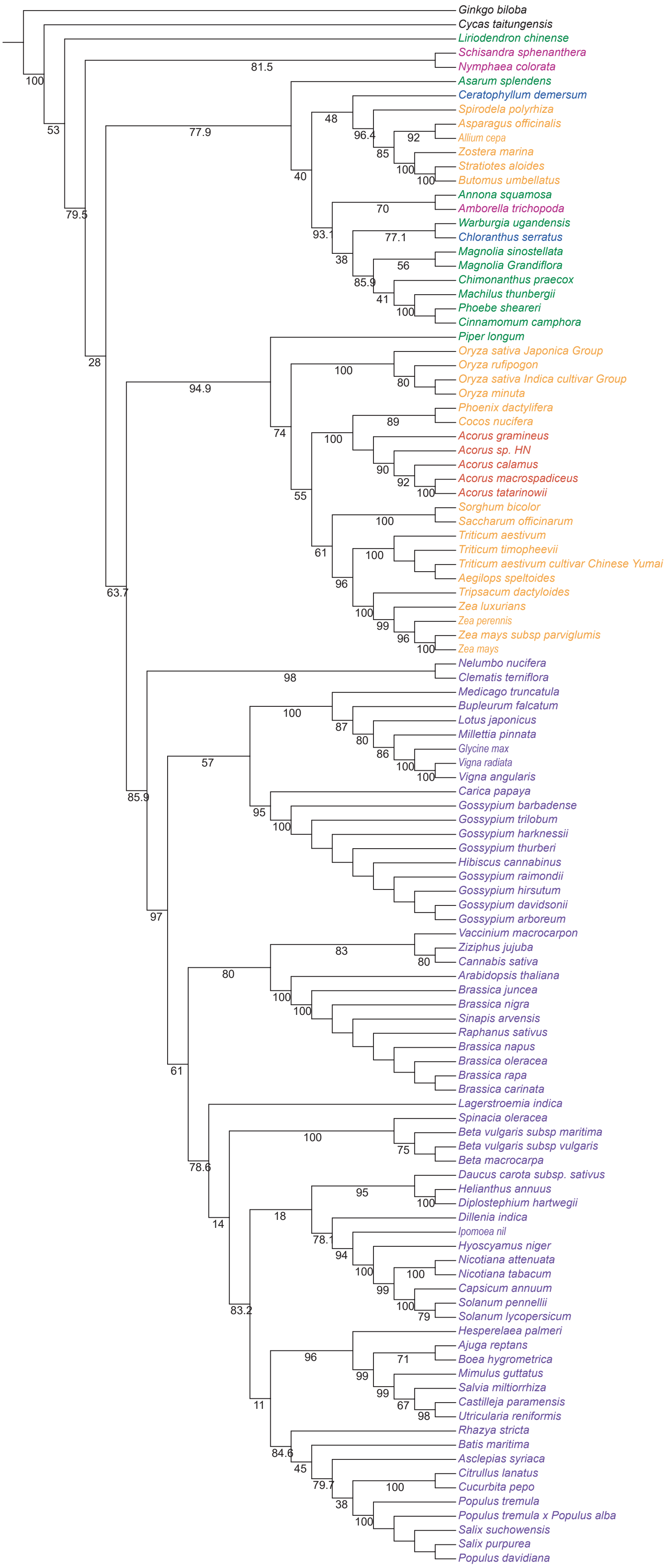

Supplementary Figure 30. The tree of mitochondrial gene *nad6* in *Acorus gramineus* and land plants. ANA groups are marked with pink, Magnoliids are marked with green, Mononcots are marked with yellow, Eudicots are marked with purple, Chloranthales and Ceratophyllales are marked with blue, and *Acorus* are marked with red. Source data underlying Supplementary Figure 30 are provided as a Source Data file.

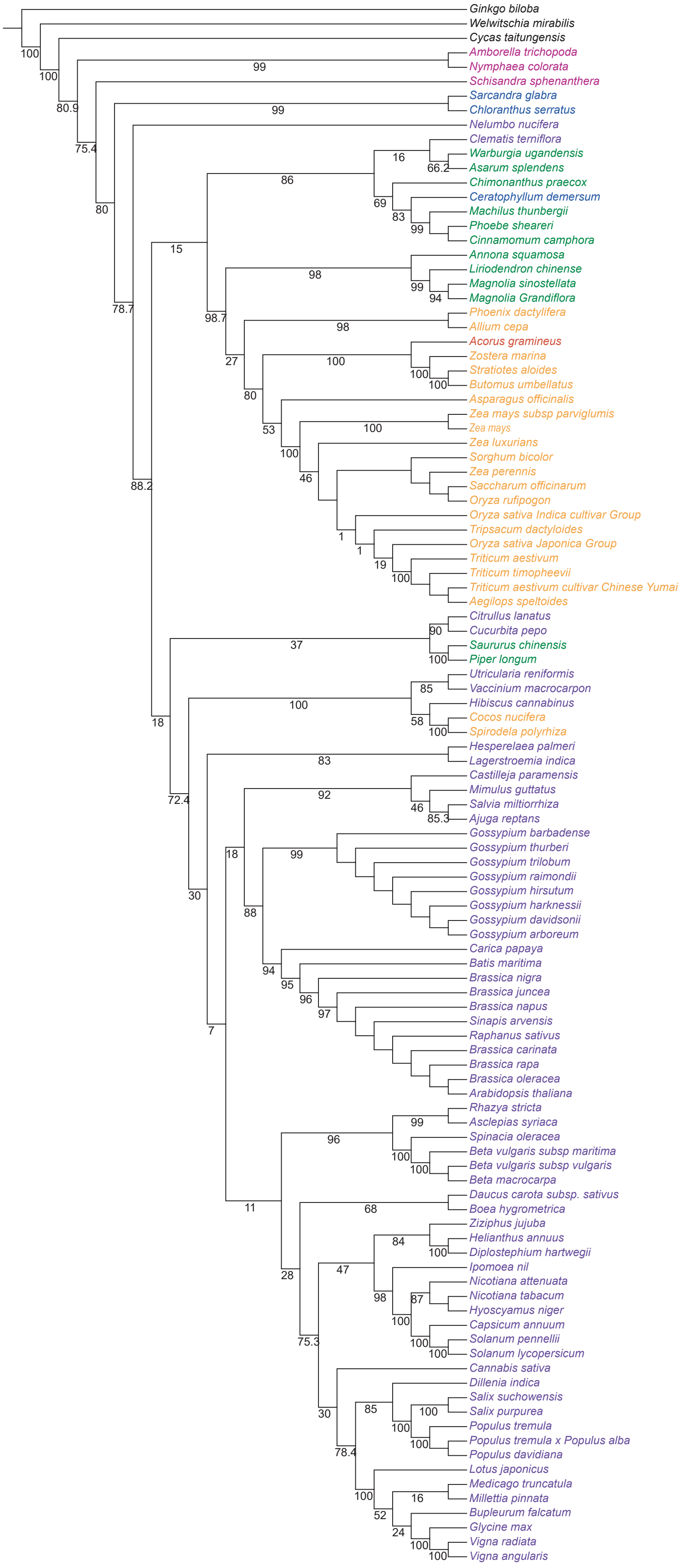

Supplementary Figure 31. The tree of mitochondrial gene *nad7* in *Acorus gramineus* and land plants. ANA groups are marked with pink, Magnoliids are marked with green, Mononcots are marked with yellow, Eudicots are marked with purple, Chloranthales and Ceratophyllales are marked with blue, and *Acorus* are marked with red. Source data underlying Supplementary Figure 31 are provided as a Source Data file.

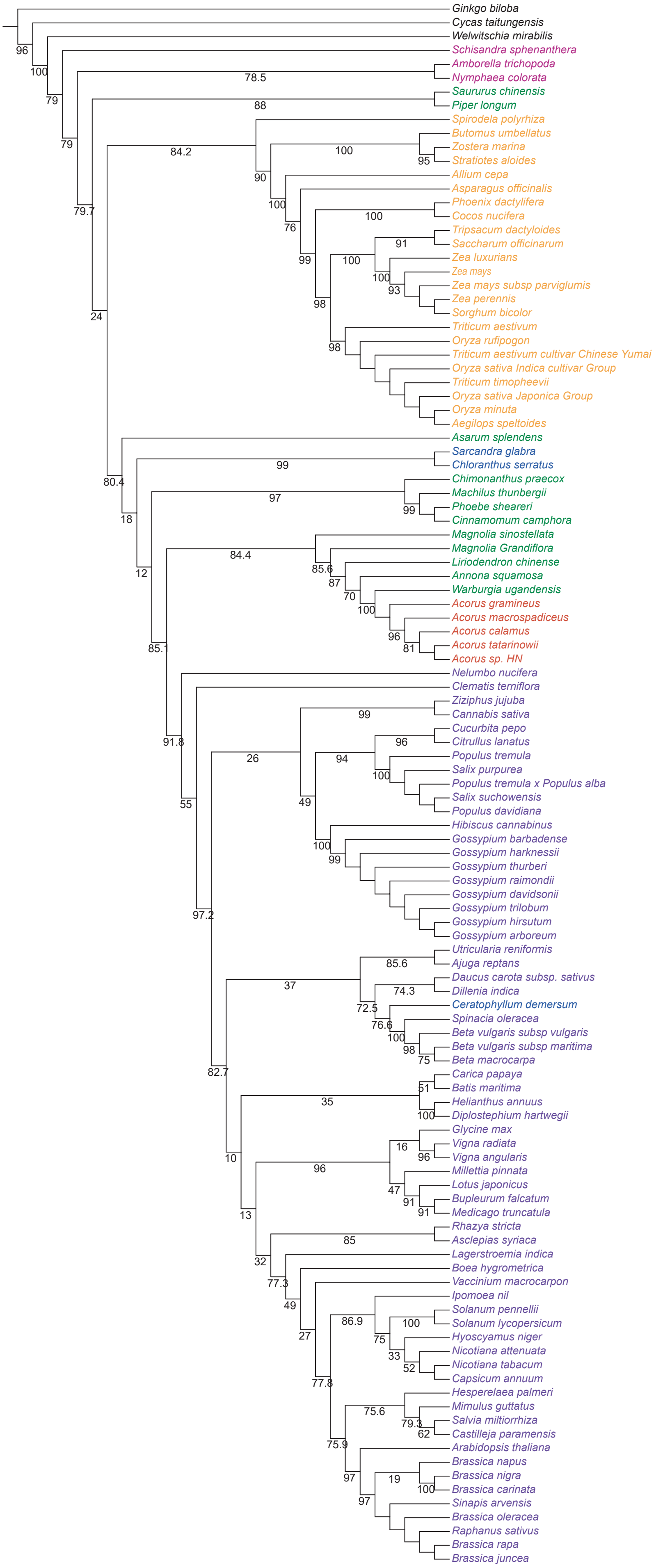

Supplementary Figure 32. The tree of mitochondrial gene *nad9* in *Acorus gramineus* and land plants. ANA groups are marked with pink, Magnoliids are marked with green, Mononcots are marked with yellow, Eudicots are marked with purple, Chloranthales and Ceratophyllales are marked with blue, and *Acorus* are marked with red. Source data underlying Supplementary Figure 32 are provided as a Source Data file.

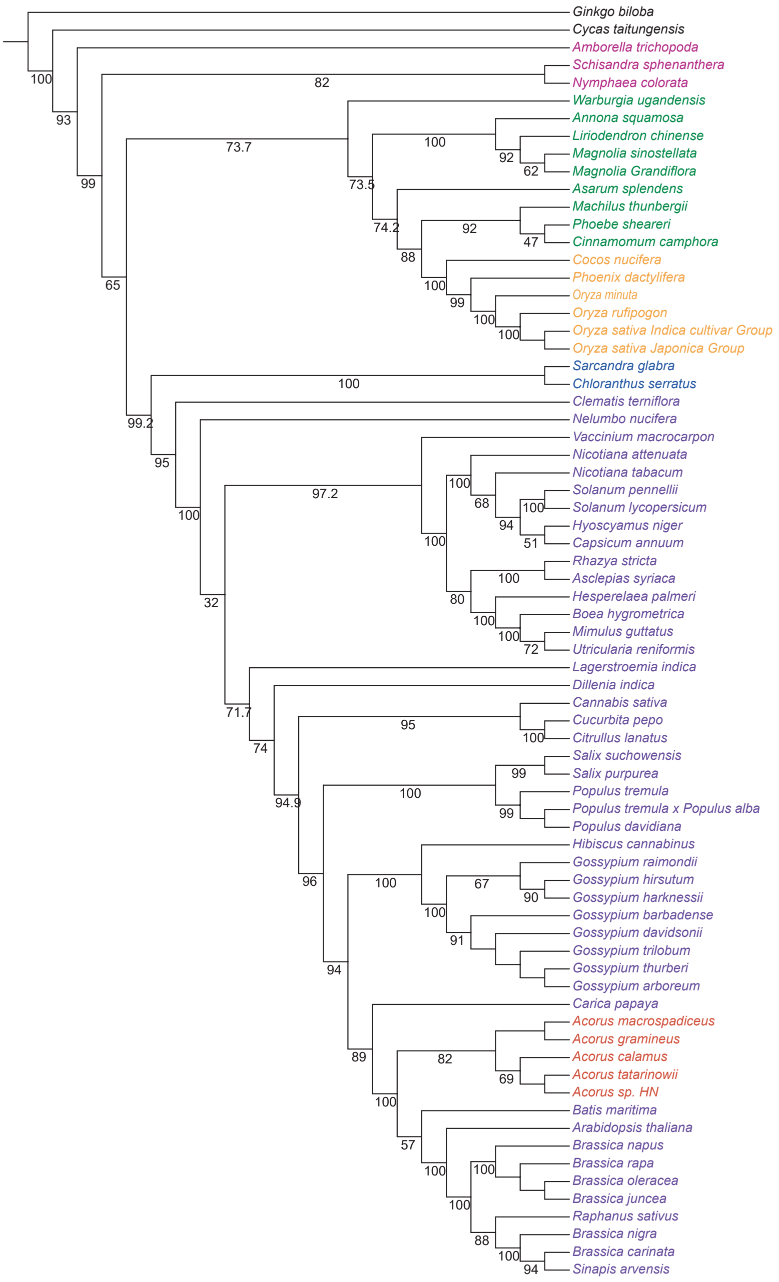

Supplementary Figure 33. The tree of mitochondrial gene *rpl2* in *Acorus gramineus* and land plants. ANA groups are marked with pink, Magnoliids are marked with green, Mononcots are marked with yellow, Eudicots are marked with purple, Chloranthales and Ceratophyllales are marked with blue, and *Acorus* are marked with red. Source data underlying Supplementary Figure 33 are provided as a Source Data file.

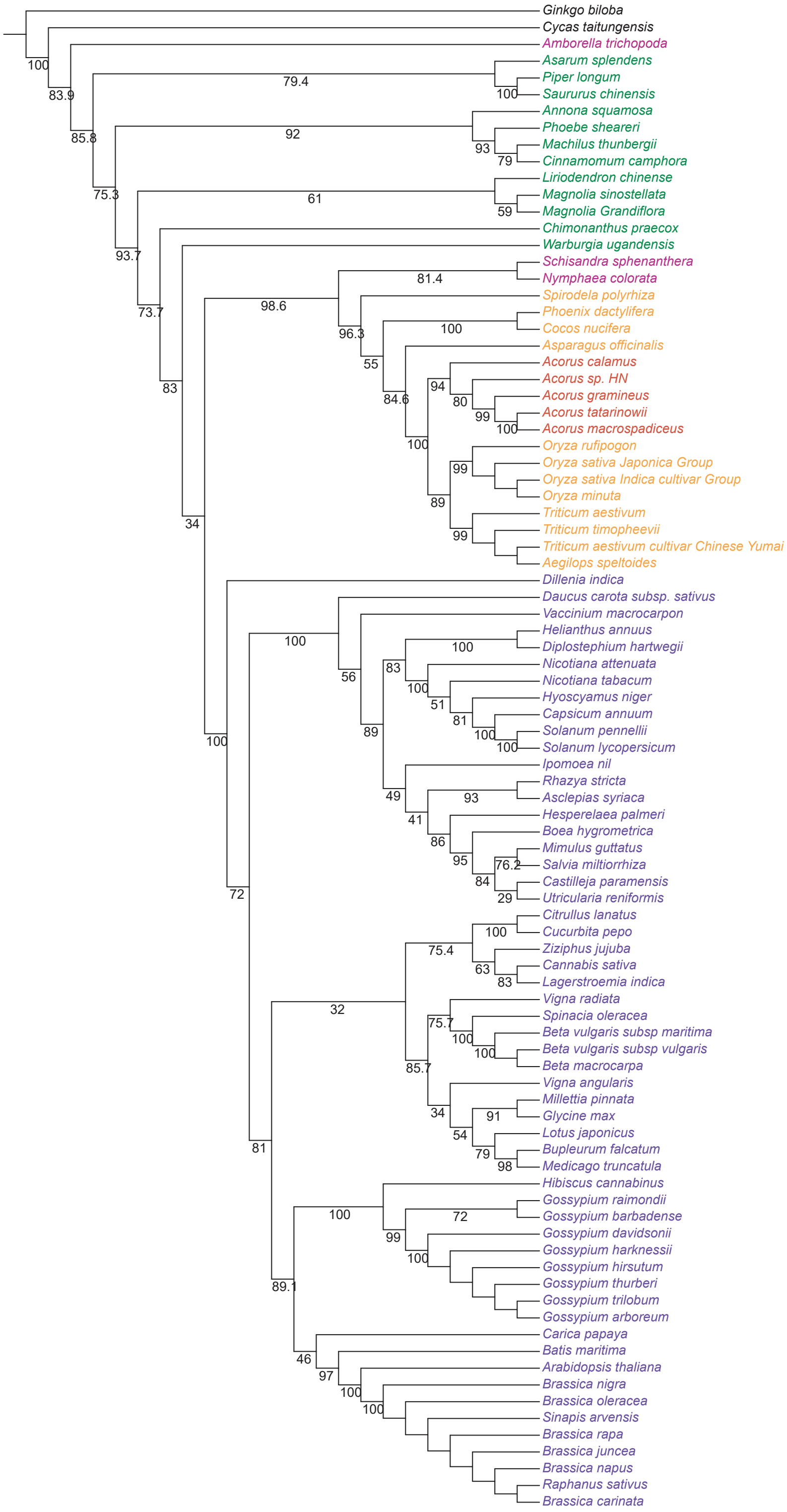

Supplementary Figure 34. The tree of mitochondrial gene *rpl5* in *Acorus gramineus* and land plants. ANA groups are marked with pink, Magnoliids are marked with green, Mononcots are marked with yellow, Eudicots are marked with purple, Chloranthales and Ceratophyllales are marked with blue, and *Acorus* are marked with red. Source data underlying Supplementary Figure 34 are provided as a Source Data file.

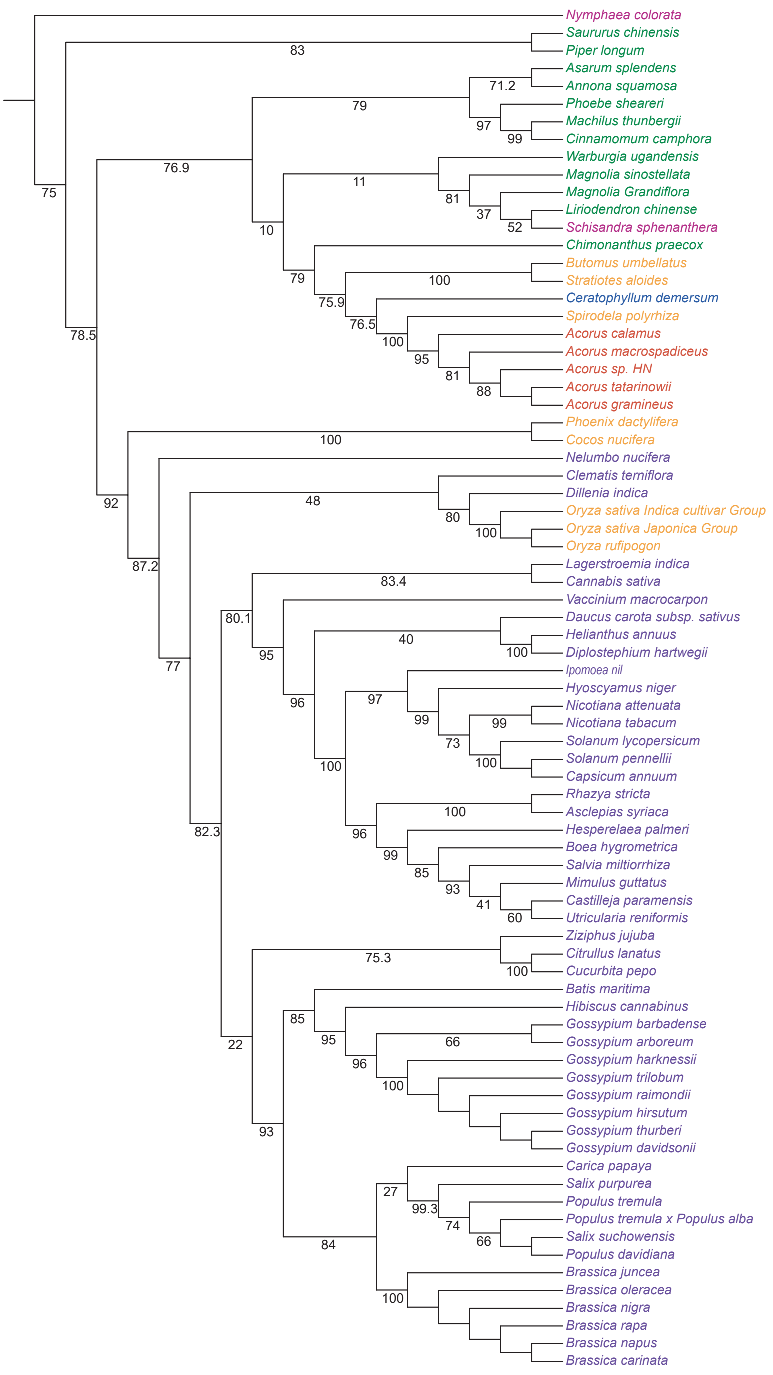

Supplementary Figure 35. The tree of mitochondrial gene *rpl10* in *Acorus gramineus* and land plants. ANA groups are marked with pink, Magnoliids are marked with green, Mononcots are marked with yellow, Eudicots are marked with purple, Chloranthales and Ceratophyllales are marked with blue, and *Acorus* are marked with red. Source data underlying Supplementary Figure 35 are provided as a Source Data file.

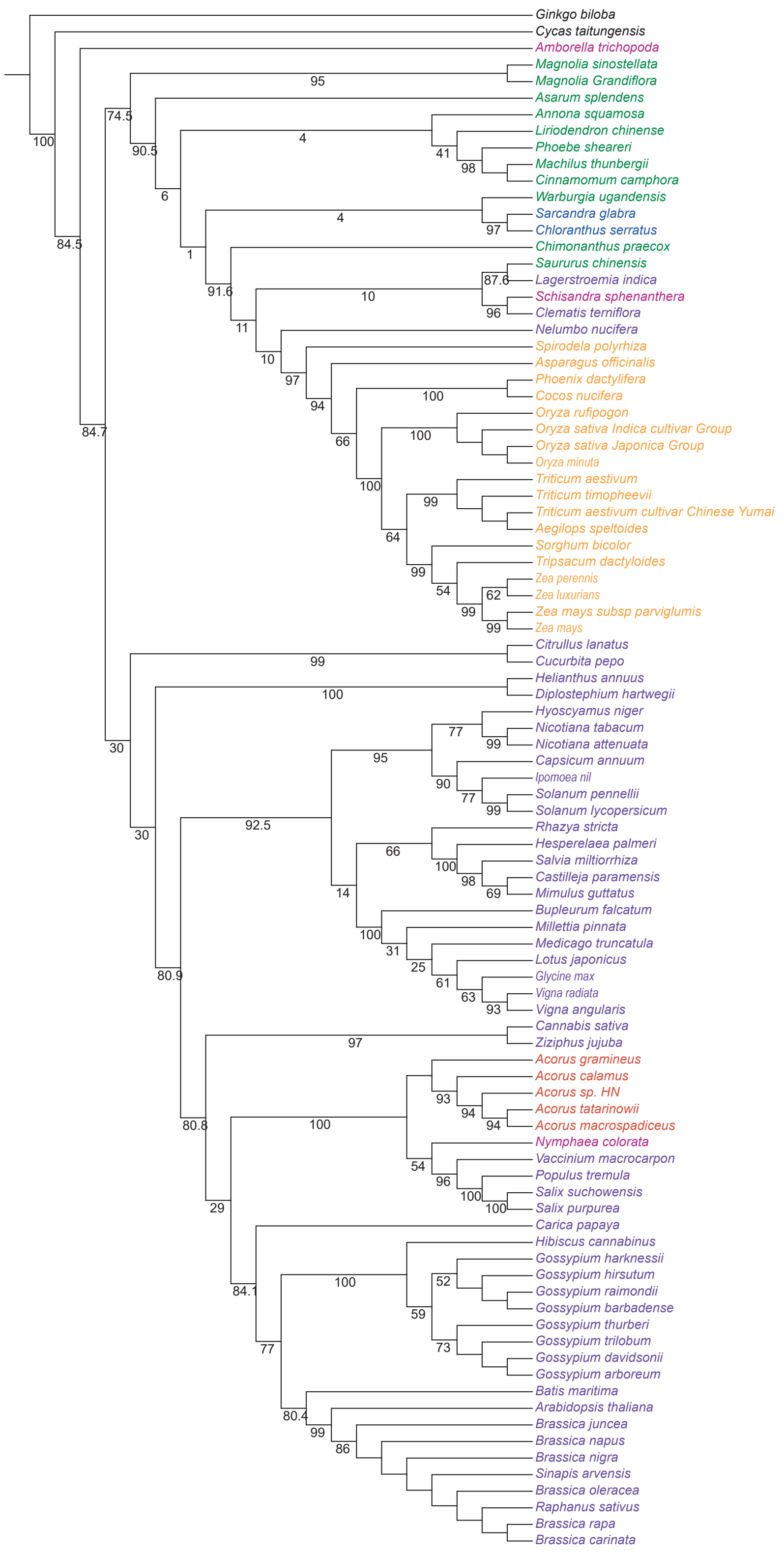

Supplementary Figure 36. The tree of mitochondrial gene *rpl16* in *Acorus gramineus* and land plants. ANA groups are marked with pink, Magnoliids are marked with green, Mononcots are marked with yellow, Eudicots are marked with purple, Chloranthales and Ceratophyllales are marked with blue, and *Acorus* are marked with red. Source data underlying Supplementary Figure 36 are provided as a Source Data file.

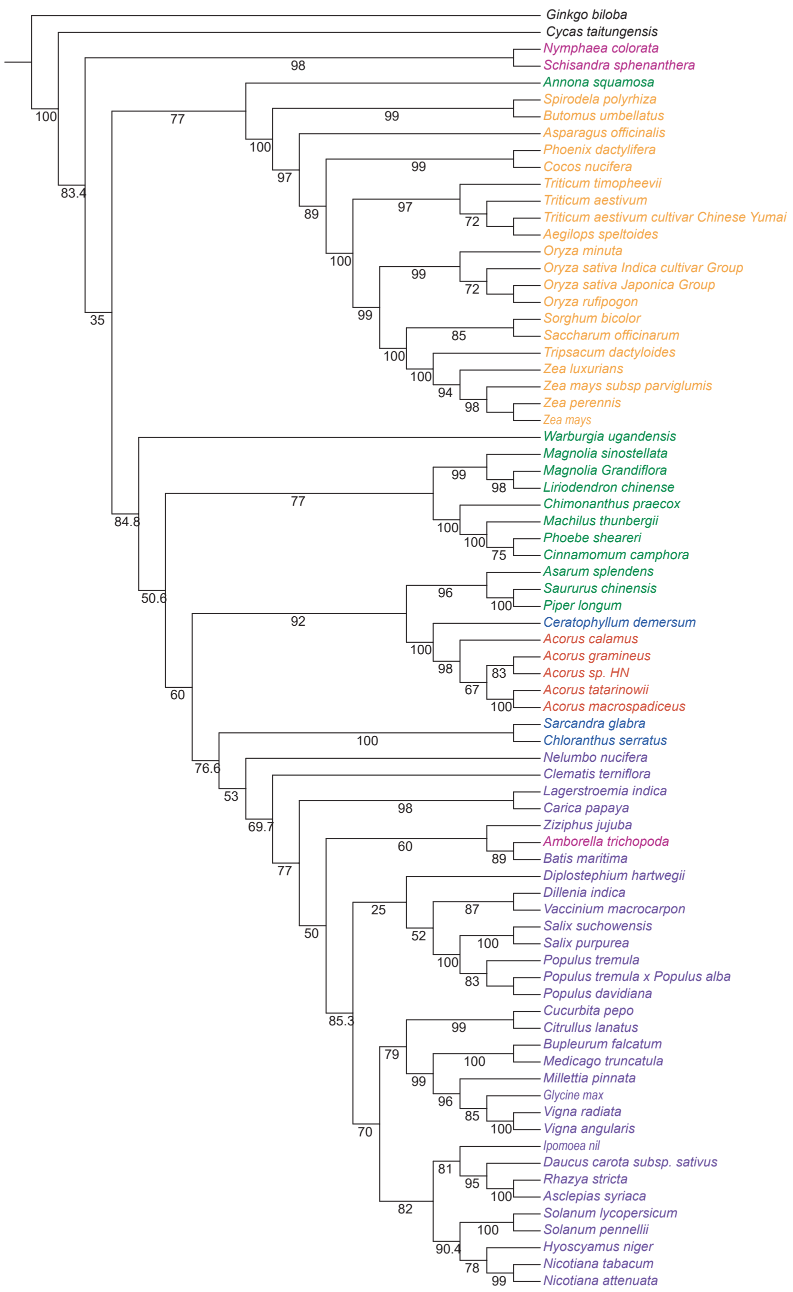

Supplementary Figure 37. The tree of mitochondrial gene *rps1* in *Acorus gramineus* and land plants. ANA groups are marked with pink, Magnoliids are marked with green, Mononcots are marked with yellow, Eudicots are marked with purple, Chloranthales and Ceratophyllales are marked with blue, and *Acorus* are marked with red. Source data underlying Supplementary Figure 37 are provided as a Source Data file.

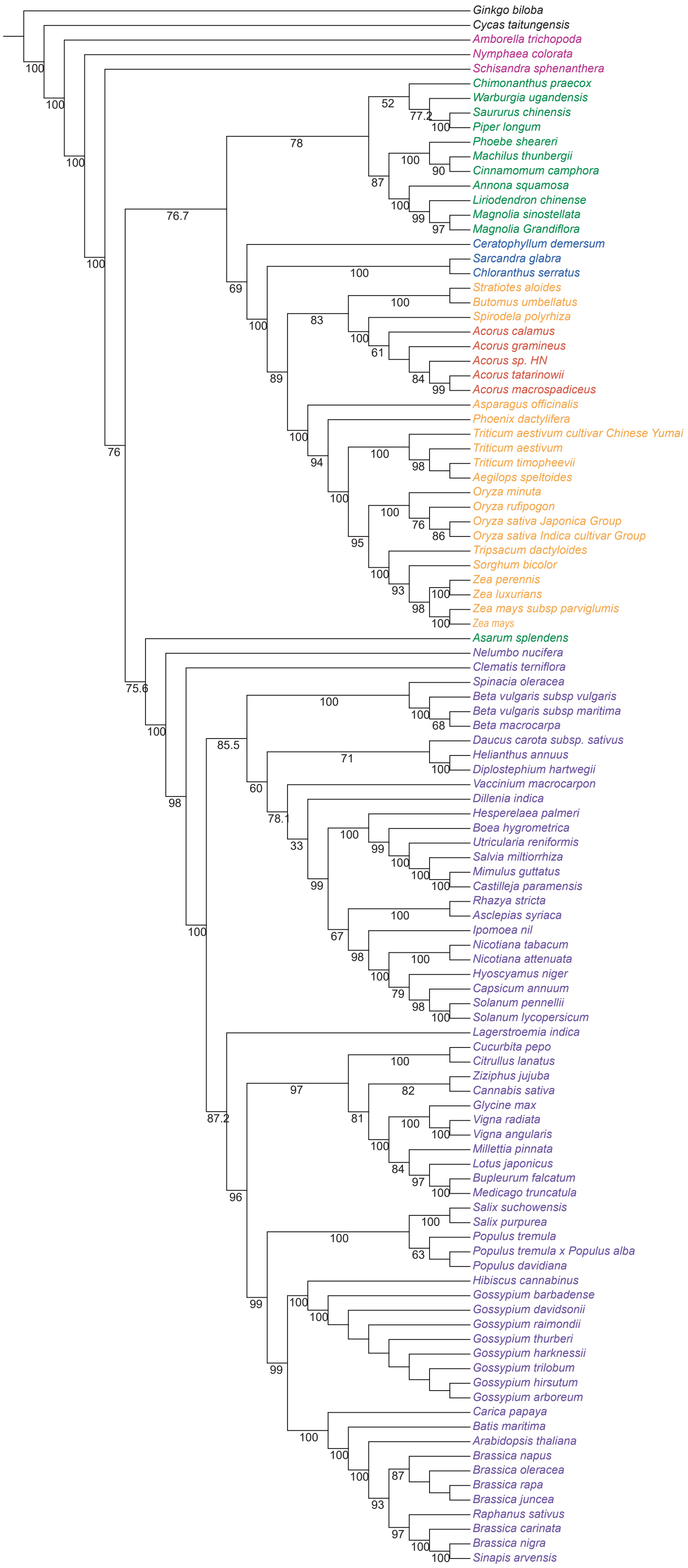

Supplementary Figure 38. The tree of mitochondrial gene *rps3* in *Acorus gramineus* and land plants. ANA groups are marked with pink, Magnoliids are marked with green, Mononcots are marked with yellow, Eudicots are marked with purple, Chloranthales and Ceratophyllales are marked with blue, and *Acorus* are marked with red. Source data underlying Supplementary Figure 38 are provided as a Source Data file.

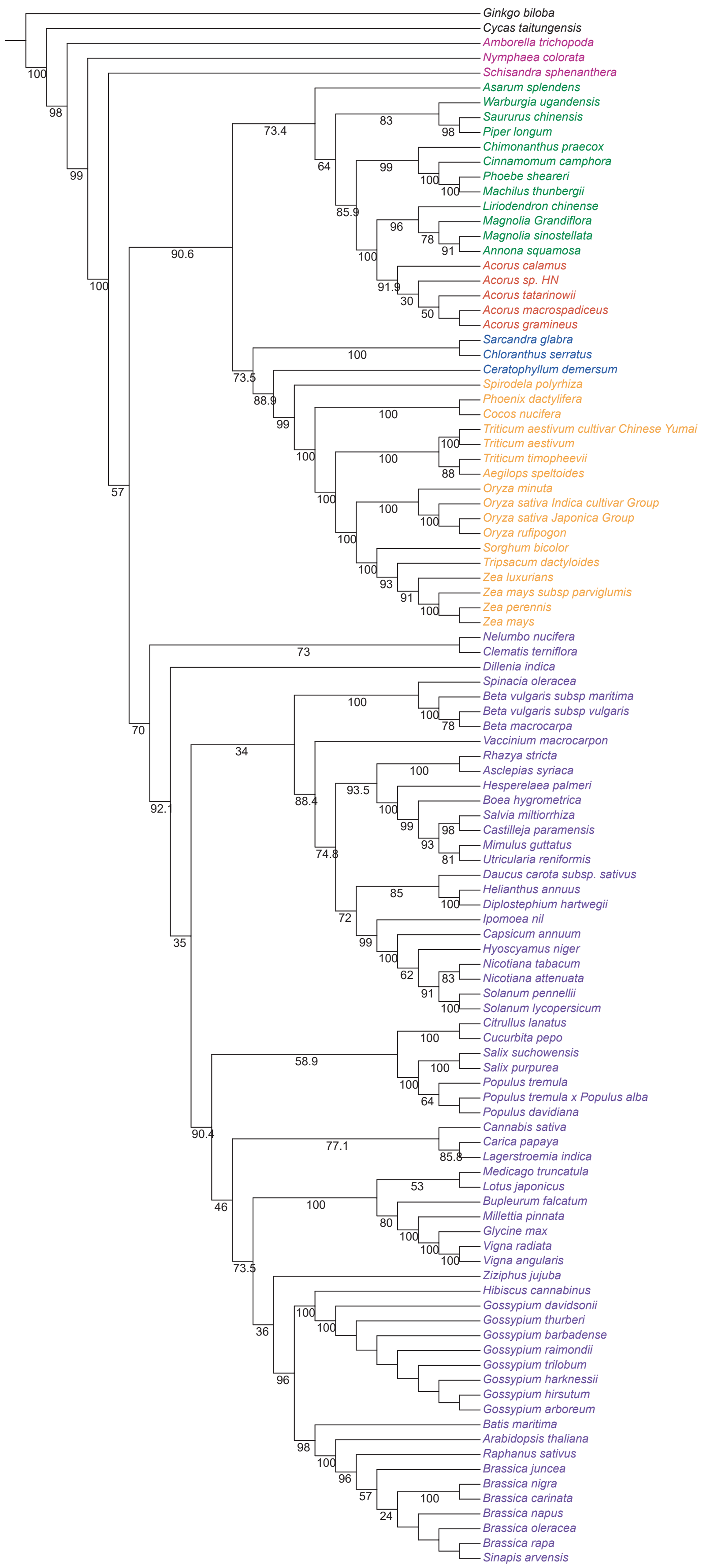

Supplementary Figure 39. The tree of mitochondrial gene *rps4* in *Acorus gramineus* and land plants. ANA groups are marked with pink, Magnoliids are marked with green, Mononcots are marked with yellow, Eudicots are marked with purple, Chloranthales and Ceratophyllales are marked with blue, and *Acorus* are marked with red. Source data underlying Supplementary Figure 39 are provided as a Source Data file.

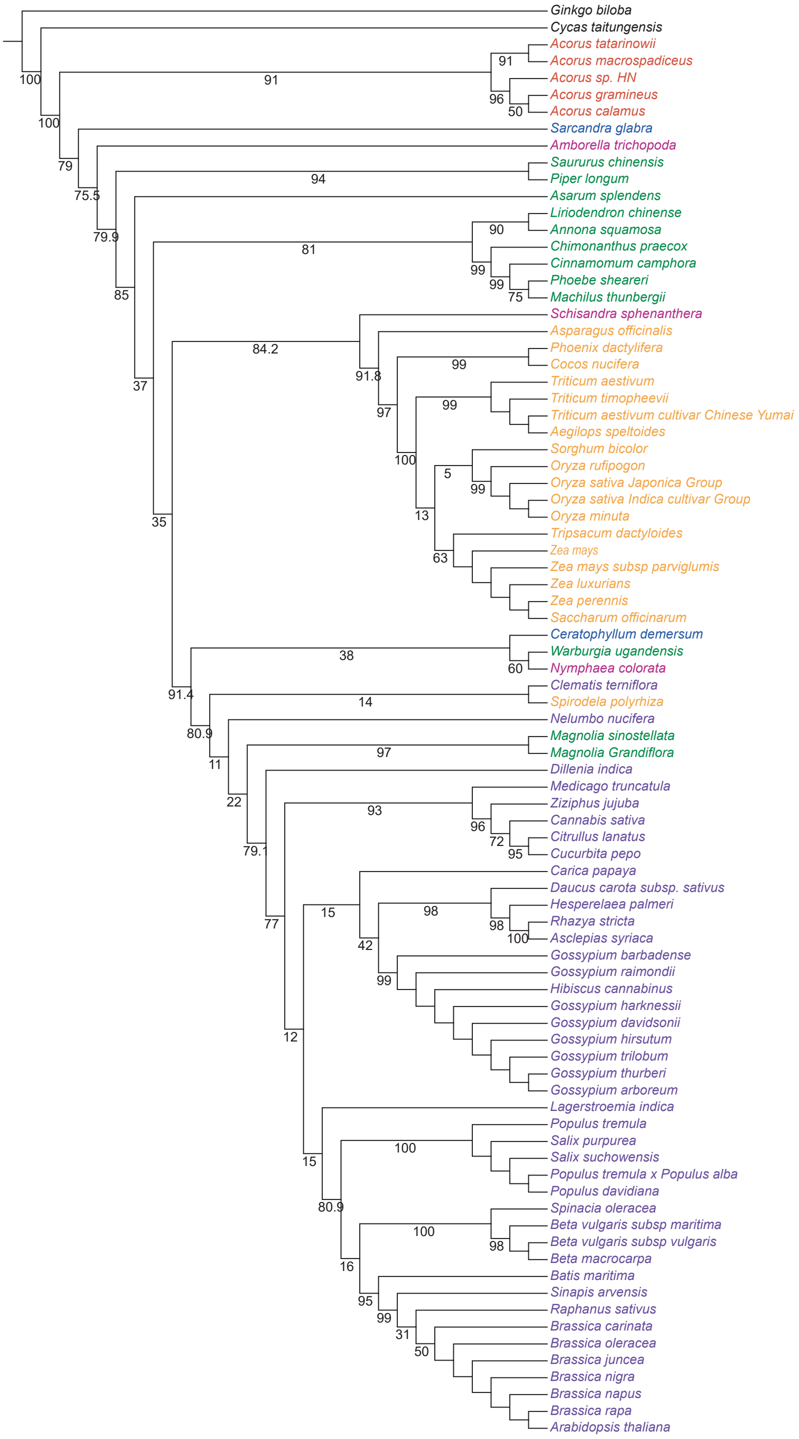

Supplementary Figure 40. The tree of mitochondrial gene *rps7* in *Acorus gramineus* and land plants. ANA groups are marked with pink, Magnoliids are marked with green, Mononcots are marked with yellow, Eudicots are marked with purple, Chloranthales and Ceratophyllales are marked with blue, and *Acorus* are marked with red. Source data underlying Supplementary Figure 40 are provided as a Source Data file.

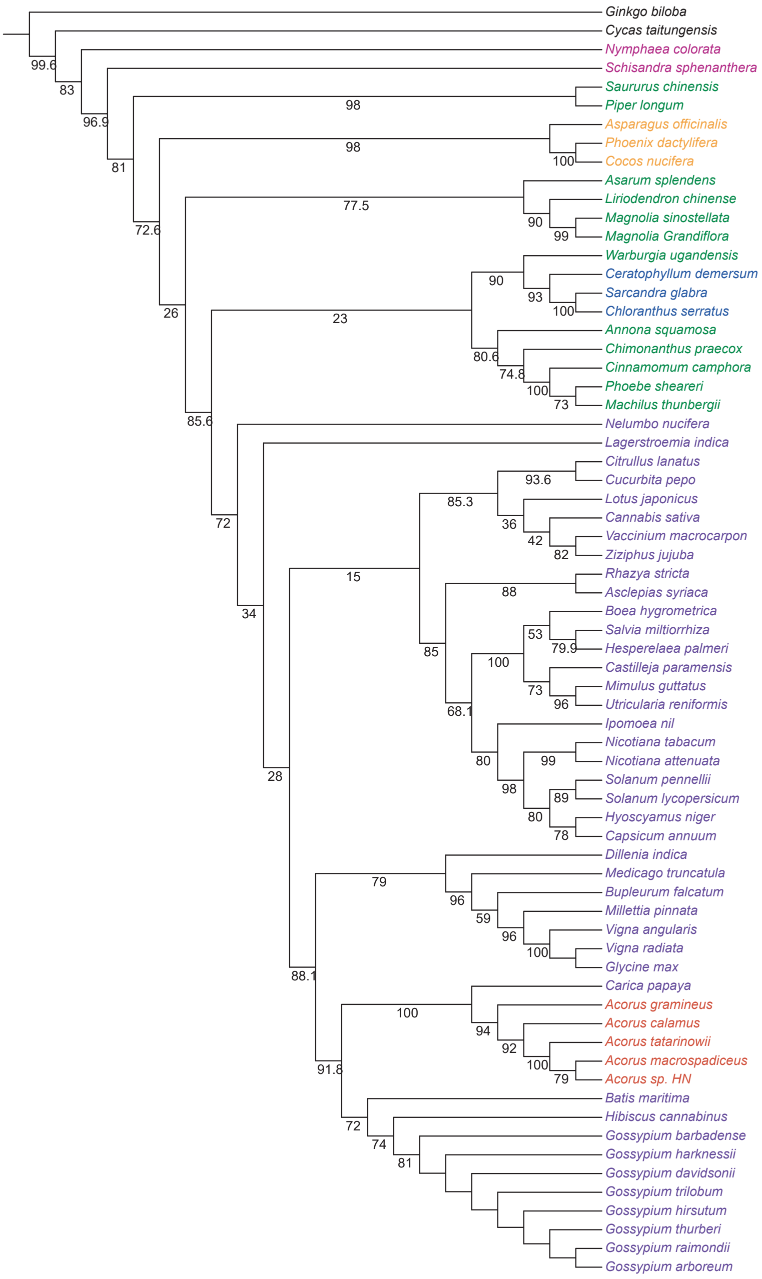

Supplementary Figure 41. The tree of mitochondrial gene *rps10* in *Acorus gramineus* and land plants. ANA groups are marked with pink, Magnoliids are marked with green, Mononcots are marked with yellow, Eudicots are marked with purple, Chloranthales and Ceratophyllales are marked with blue, and *Acorus* are marked with red. Source data underlying Supplementary Figure 41 are provided as a Source Data file.

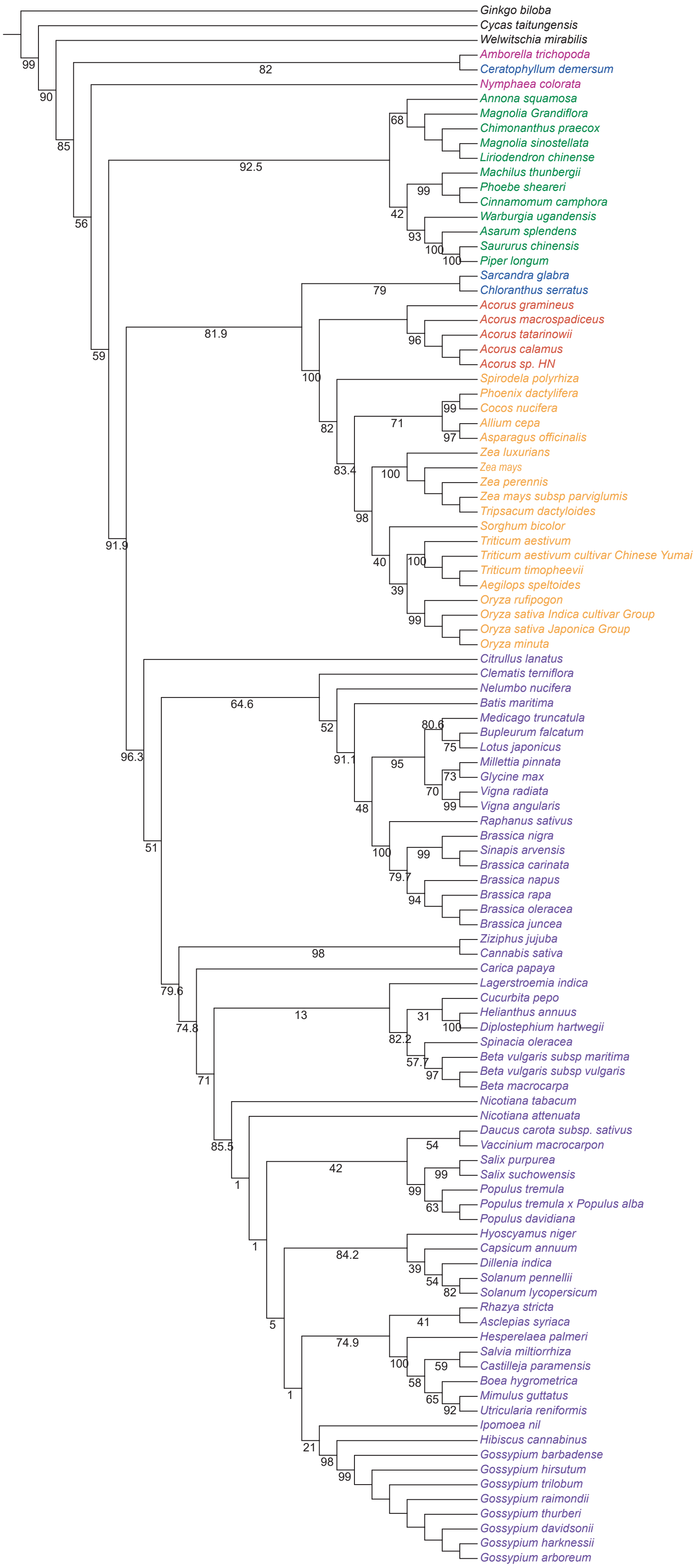

Supplementary Figure 42. The tree of mitochondrial gene *rps12* in *Acorus gramineus* and land plants. ANA groups are marked with pink, Magnoliids are marked with green, Mononcots are marked with yellow, Eudicots are marked with purple, Chloranthales and Ceratophyllales are marked with blue, and *Acorus* are marked with red. Source data underlying Supplementary Figure 42 are provided as a Source Data file.

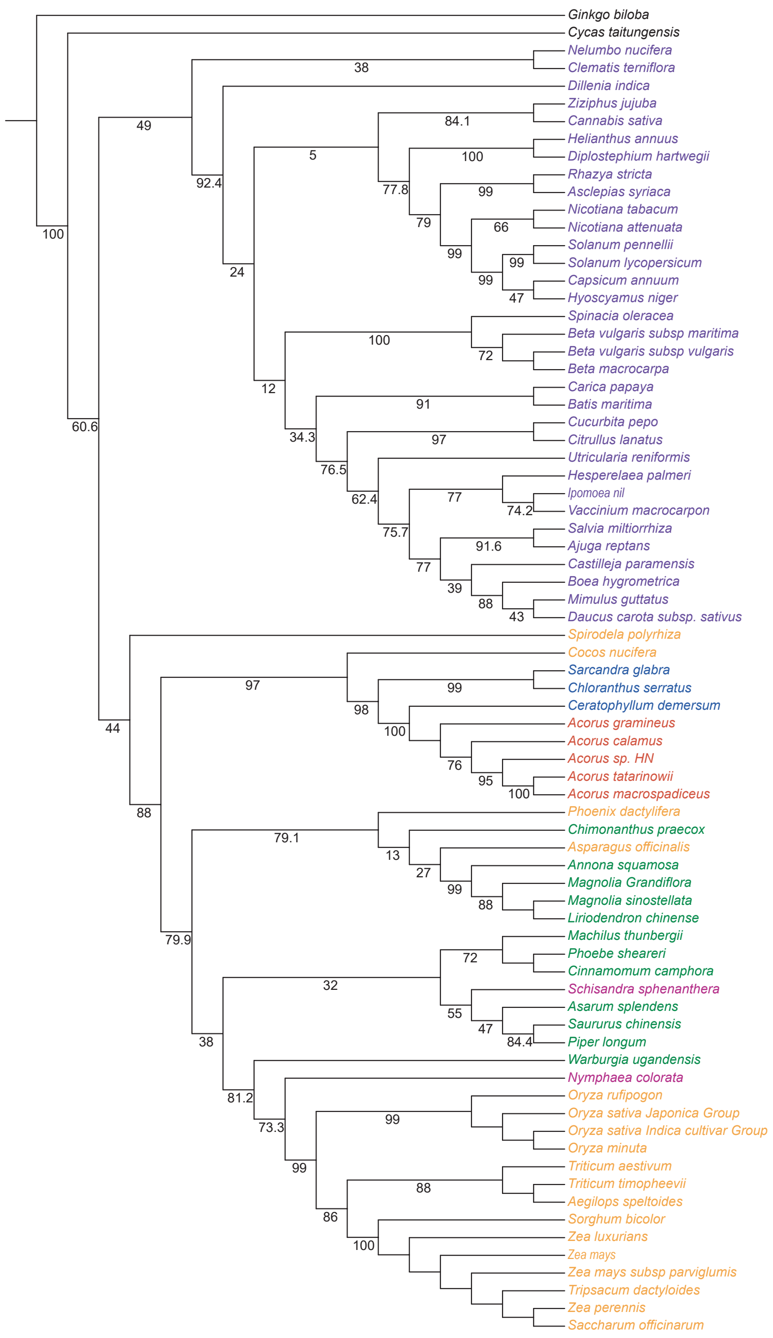

Supplementary Figure 43. The tree of mitochondrial gene *rps13* in *Acorus gramineus* and land plants. ANA groups are marked with pink, Magnoliids are marked with green, Mononcots are marked with yellow, Eudicots are marked with purple, Chloranthales and Ceratophyllales are marked with blue, and *Acorus* are marked with red. Source data underlying Supplementary Figure 43 are provided as a Source Data file.

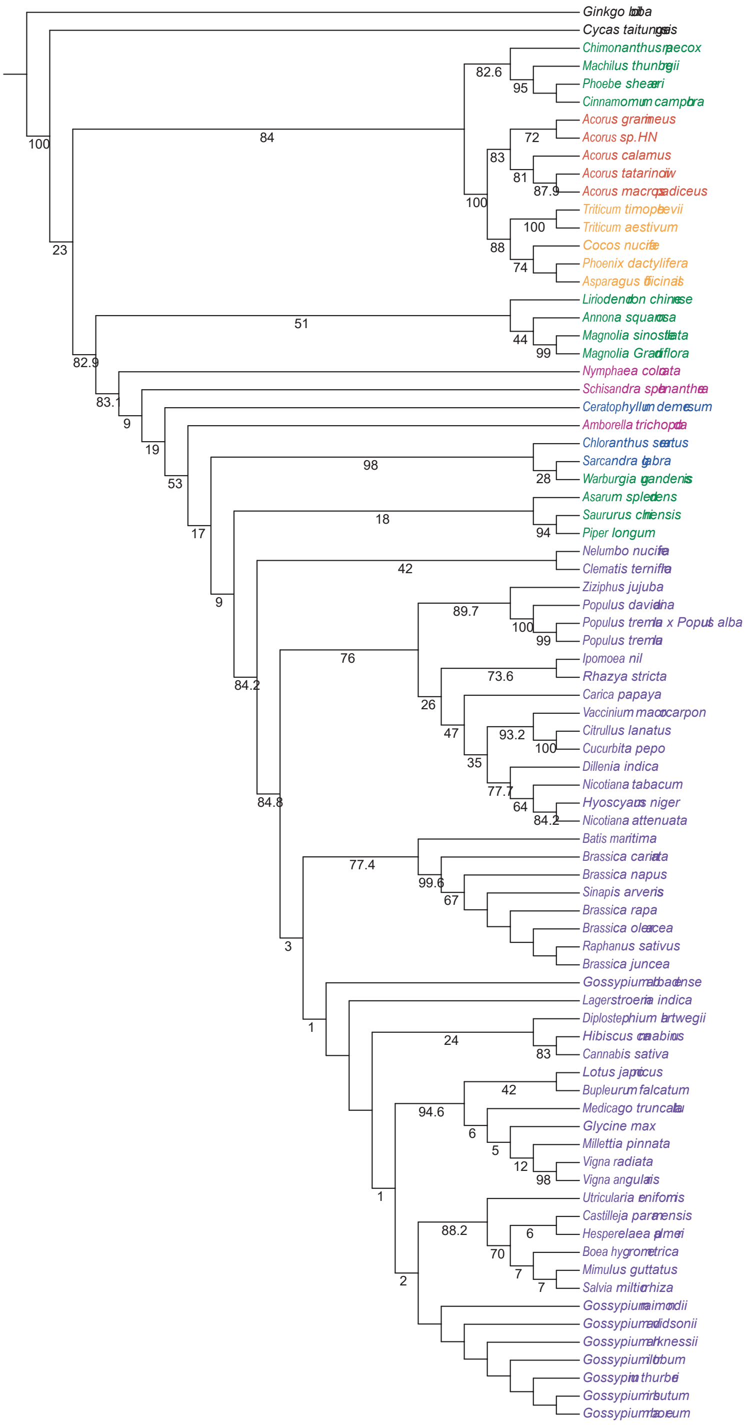

Supplementary Figure 44. The tree of mitochondrial gene *rps14* in *Acorus gramineus* and land plants. ANA groups are marked with pink, Magnoliids are marked with green, Mononcots are marked with yellow, Eudicots are marked with purple, Chloranthales and Ceratophyllales are marked with blue, and *Acorus* are marked with red. Source data underlying Supplementary Figure 44 are provided as a Source Data file.

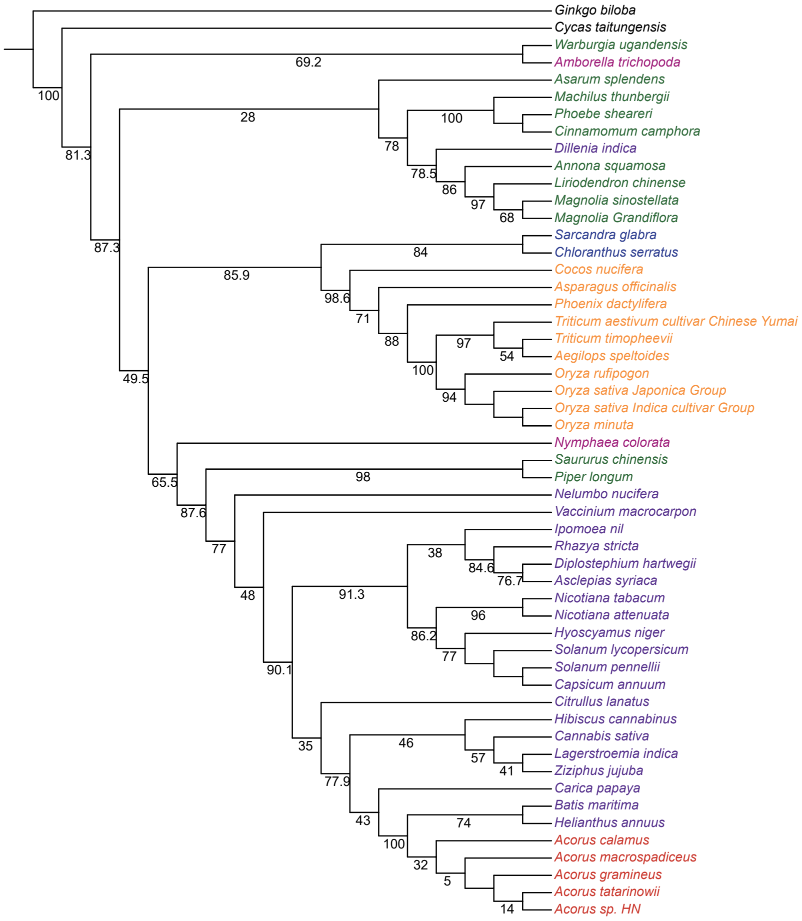

Supplementary Figure 45. The tree of mitochondrial gene *rps19* in *Acorus gramineus* and land plants. ANA groups are marked with pink, Magnoliids are marked with green, Mononcots are marked with yellow, Eudicots are marked with purple, Chloranthales and Ceratophyllales are marked with blue, and *Acorus* are marked with red. Source data underlying Supplementary Figure 45 are provided as a Source Data file.

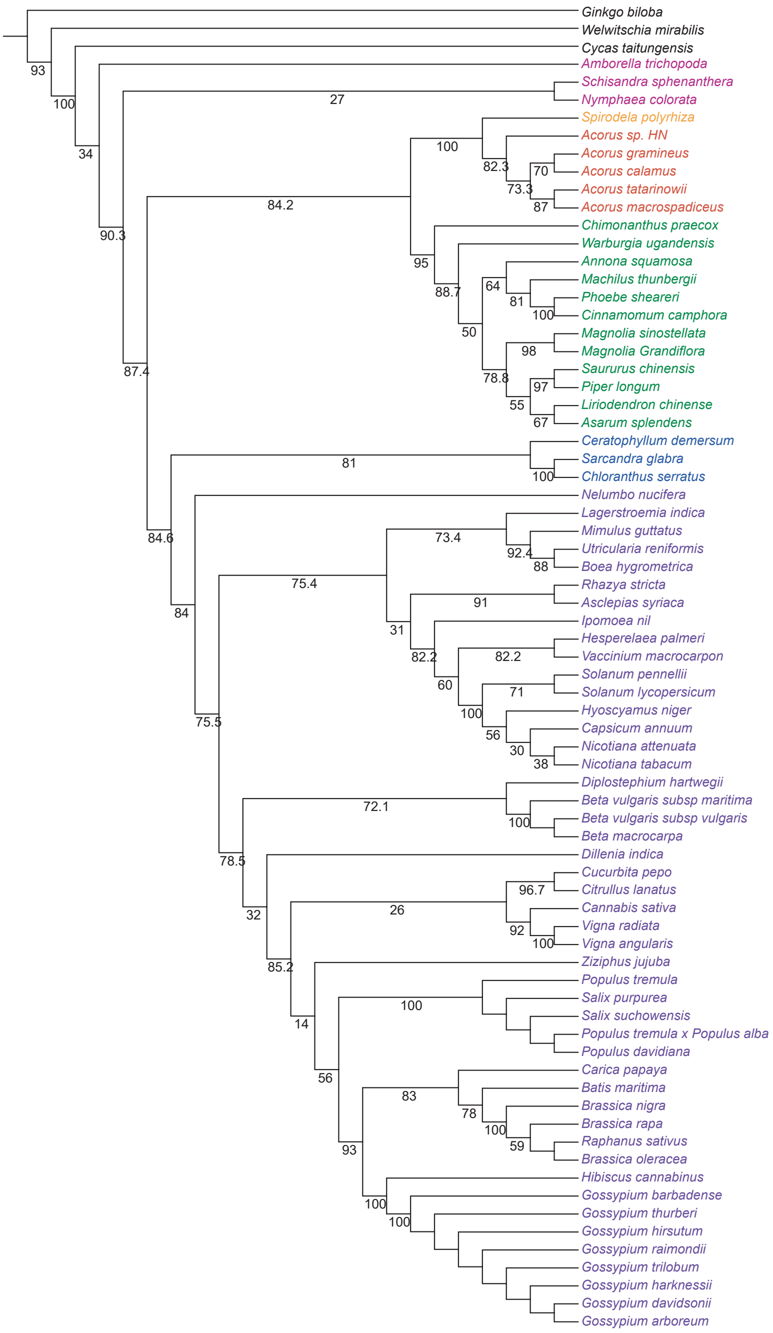

Supplementary Figure 46. The tree of mitochondrial gene *sdh4* in *Acorus gramineus* and land plants. ANA groups are marked with pink, Magnoliids are marked with green, Mononcots are marked with yellow, Eudicots are marked with purple, Chloranthales and Ceratophyllales are marked with blue, and *Acorus* are marked with red. Source data underlying Supplementary Figure 46 are provided as a Source Data file.

cob

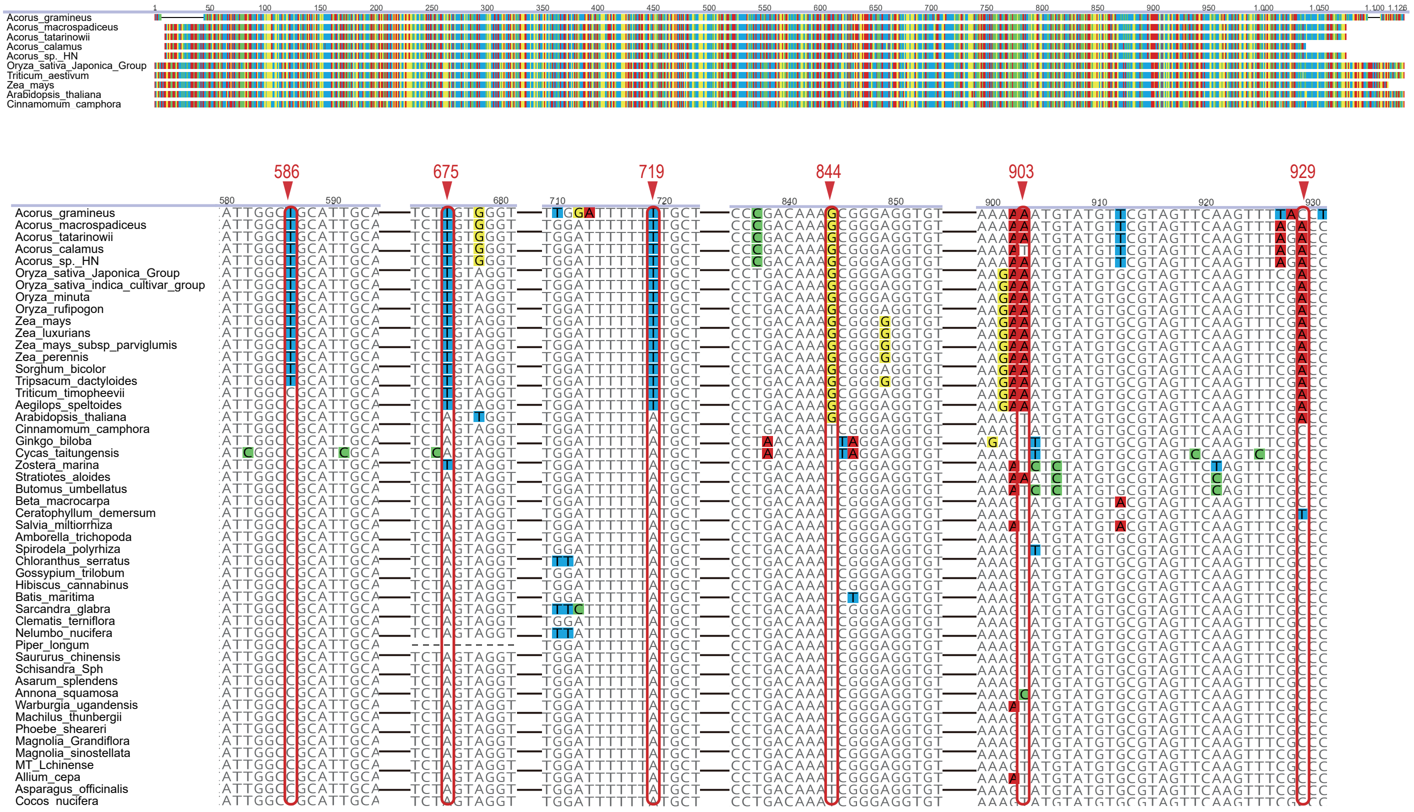

rpl2

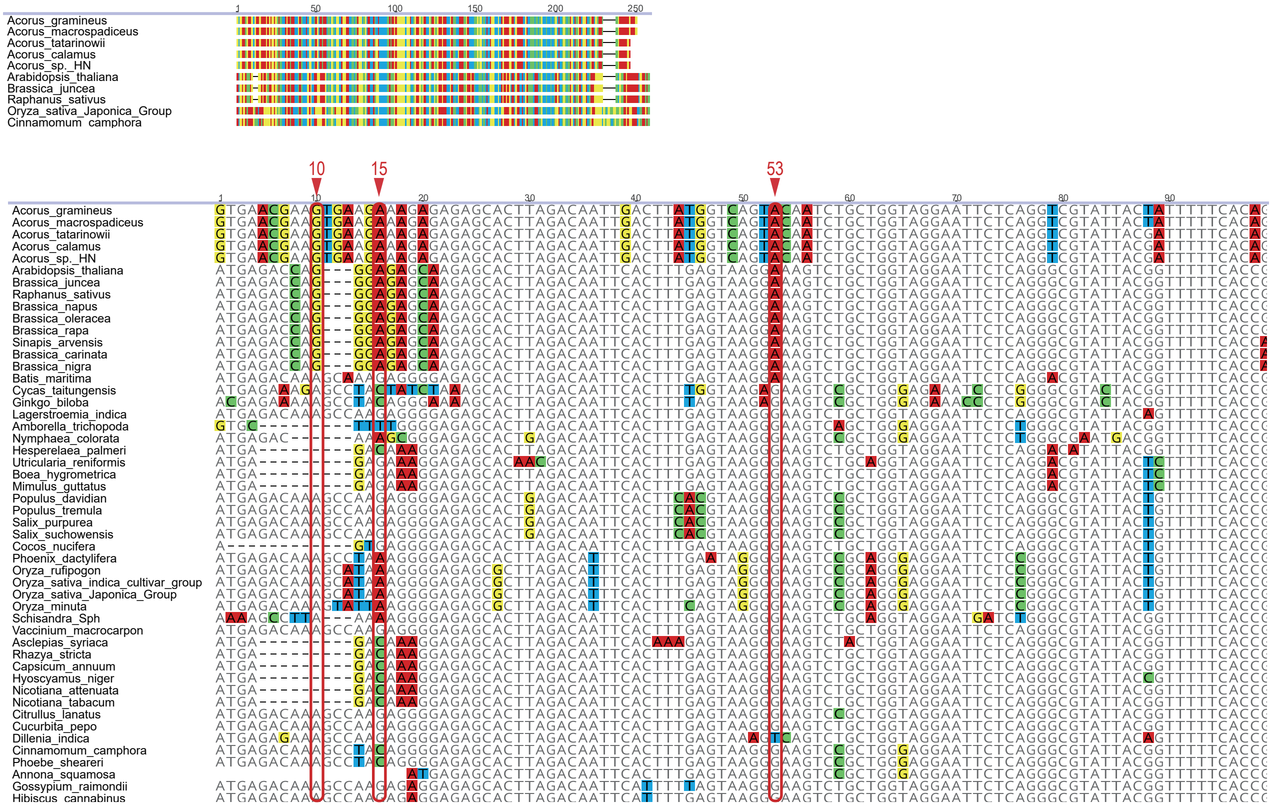

Supplementary Figure 47. The schematic sequence comparison shows *cob* and *rpl2* gene identities relative to *Acorus gramineus* and orther land plants mitochondrial genes. The sites pointed to by the arrow show the sequence discrepancies in the species. Source data underlying Supplementary Figure 47 are provided as a Source Data file.

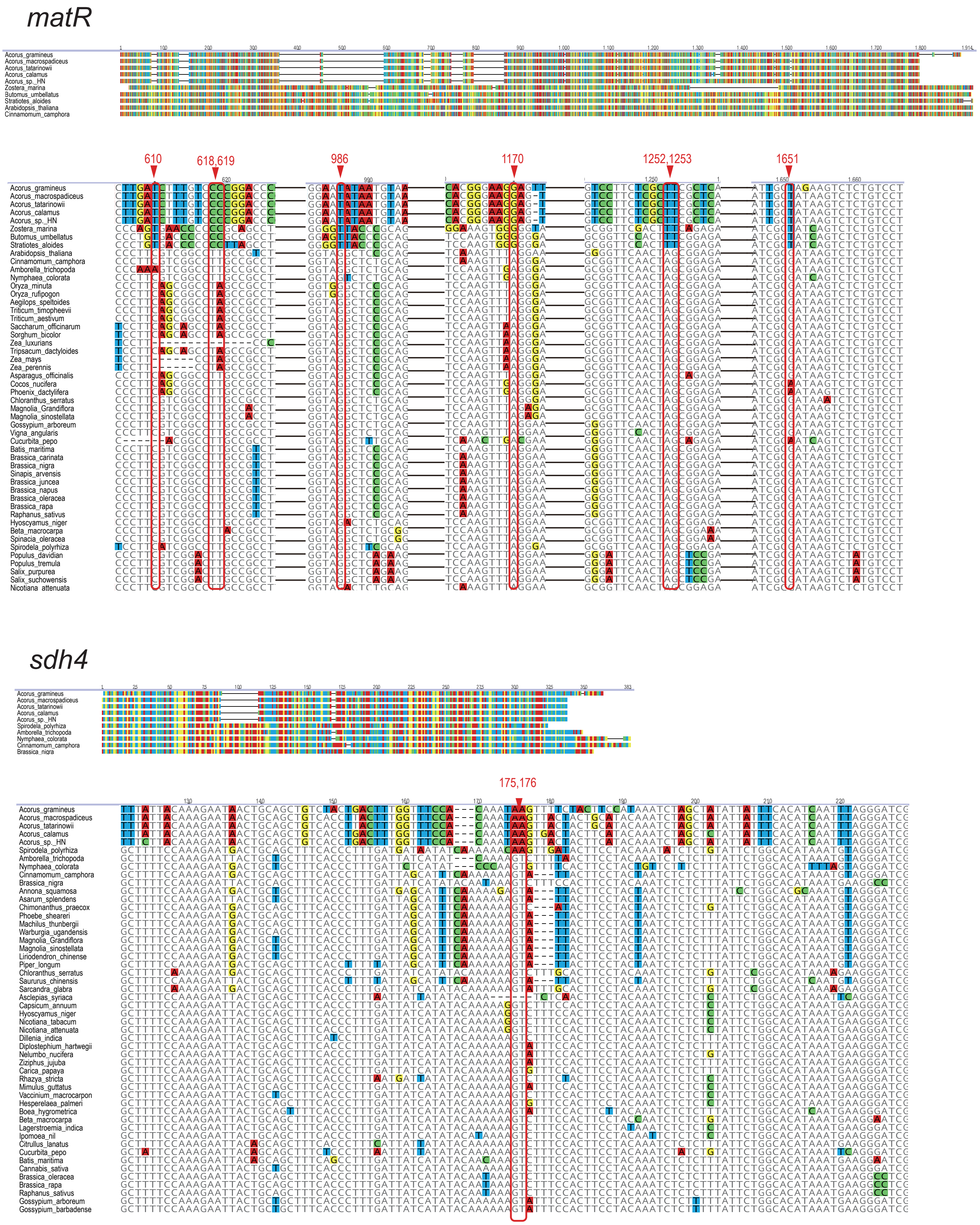

Supplementary Figure 48. The schematic sequence comparison shows *matR* and *sdh4* gene identities relative to *Acorus gramineus* and orther land plants mitochondrial genes. The sites pointed to by the arrow show the sequence discrepancies in the species. Source data underlying Supplementary Figure 48 are provided as a Source Data file.

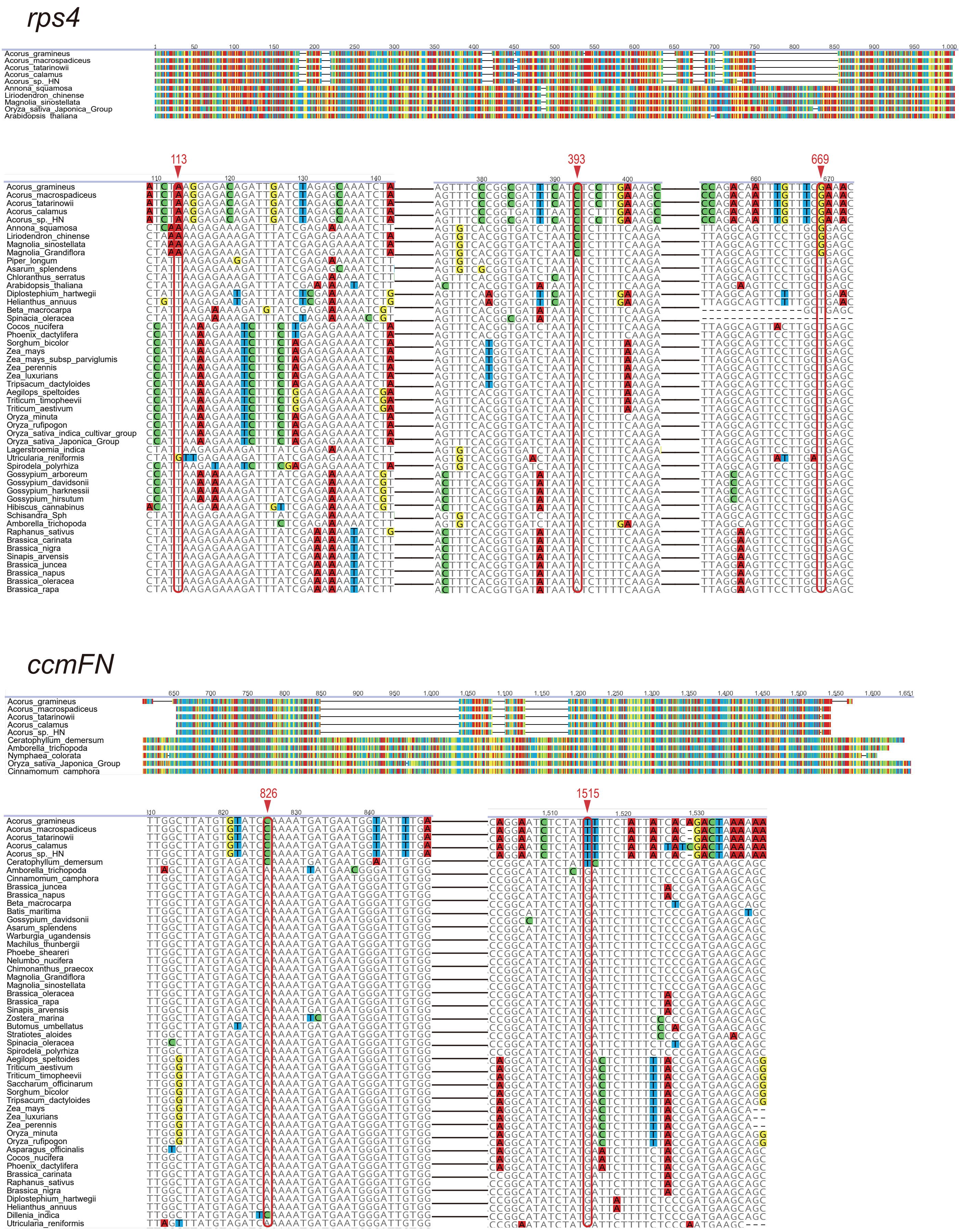

Supplementary Figure 49. The schematic sequence comparison shows *rps4* and *ccmFN* gene identities relative to *Acorus gramineus* and orther land plants mitochondrial genes. The sites pointed to by the arrow show the sequence discrepancies in the species. Source data underlying Supplementary Figure 49 are provided as a Source Data file.

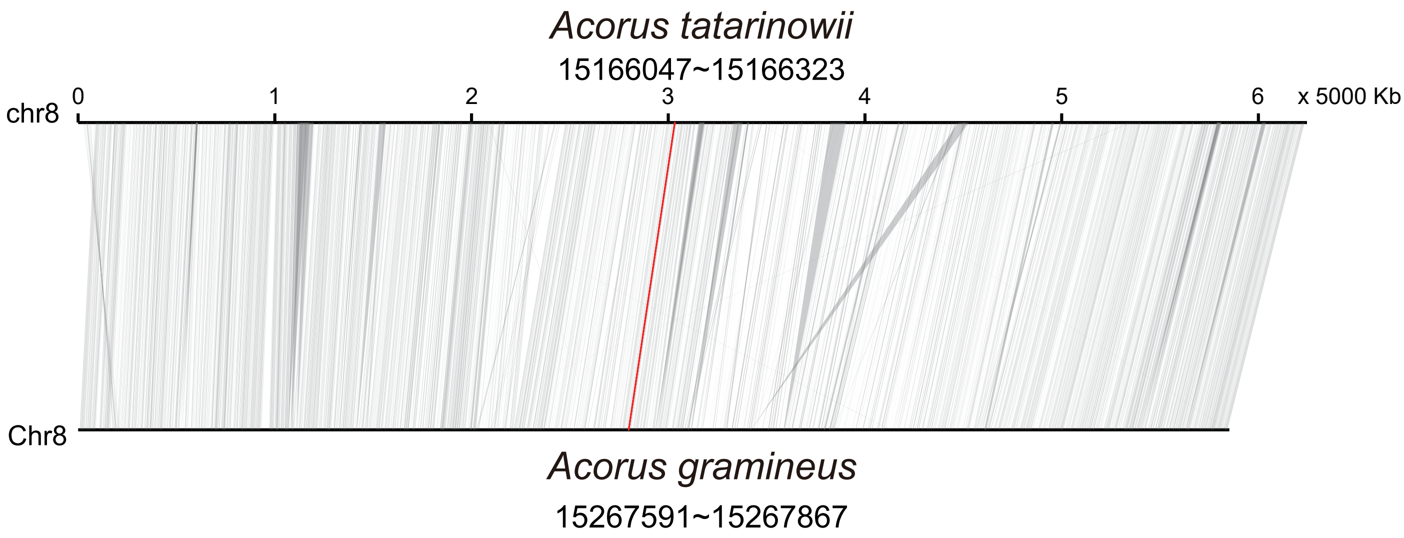

Supplementary Figure 50. Anchoring nuclear genes and mitochondrial *rps19* gene on the pseudo-chromosome 8 of the *Acorus gramineus* to the *Acorus tatarinowi*. With the genes on the chromosome 8 (29.3 Mb) of the *Acorus gramineus*, 2,181 of 2,185 nuclear genes (grey line) were anchored to *Acorus tatarinowii* chromosome8 (31.2 Mb), including the mitochondrial *rps19* gene (red line. *Acorus gramineus*,15267591,15267867, +; *Acorus tatarinowi,*15166047, 15166323,-),which was in the high consistent collinear block.

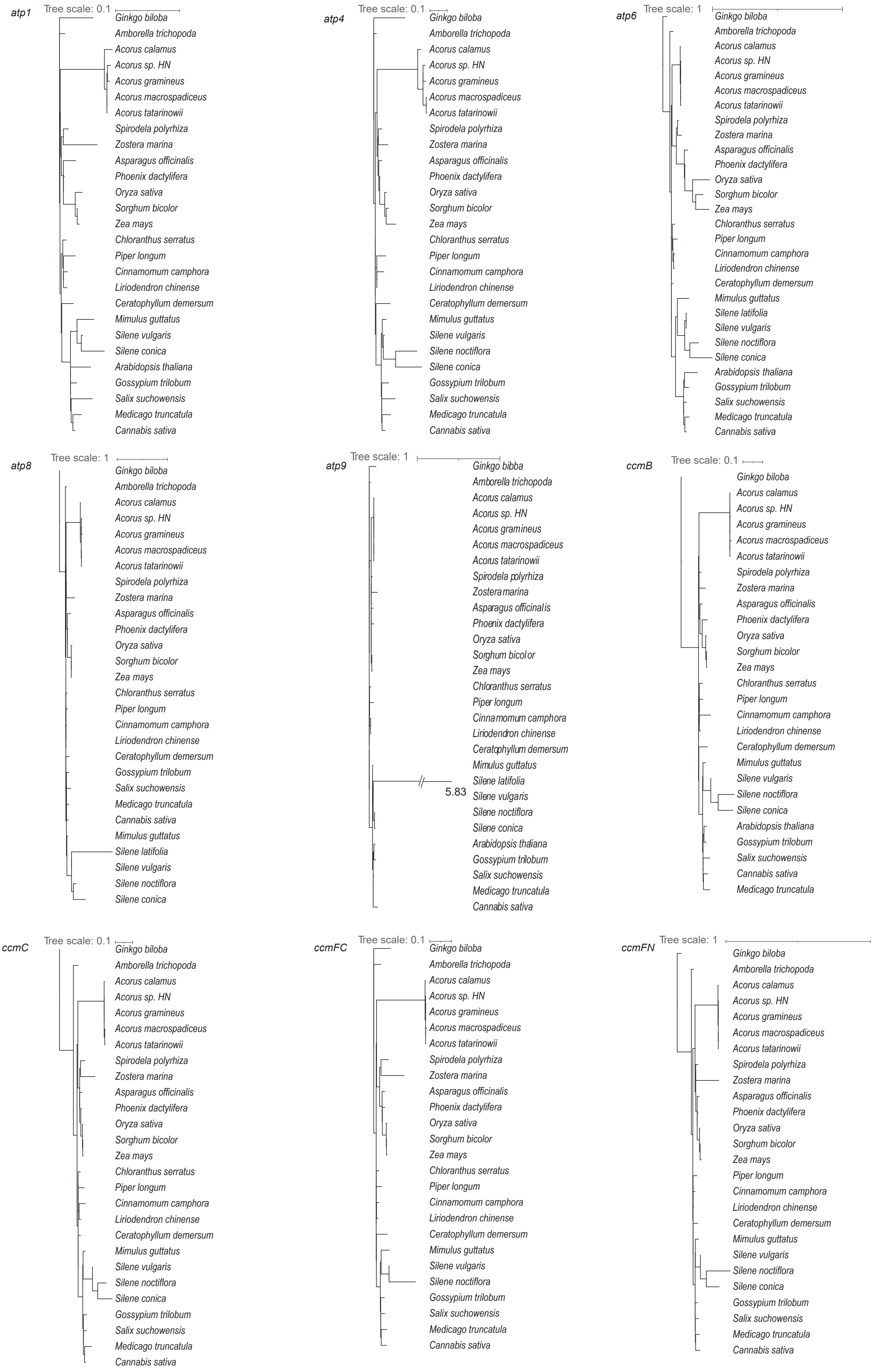

Supplementary Figure 51. Phylograms of non-synonymous nucleotide substitution per site (d_N) sequence divergence of *atp1*, *atp4*, *atp6*, *atp8*, *atp9*, *ccmB*, *ccmC*, *ccmFC* and *ccmFN* genes. Branch lengths are scaled to the value of d_N on the basis of an analysis of single gene.

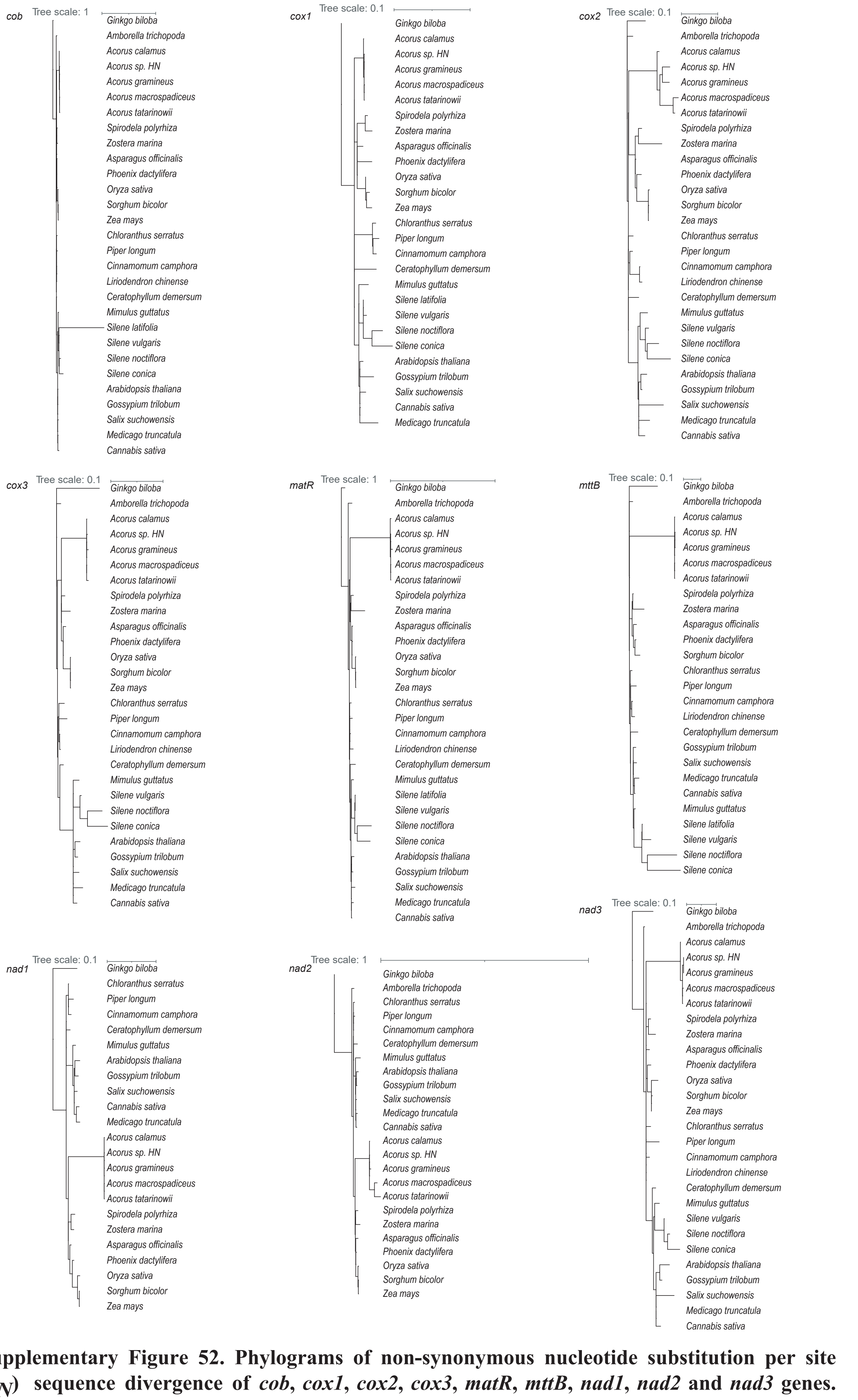

Supplementary Figure 52. Phylograms of non-synonymous nucleotide substitution per site (d_N) sequence divergence of *cob*, *cox1*, *cox2*, *cox3*, *matR*, *mttB*, *nad1*, *nad2* and *nad3* genes. Branch lengths are scaled to the value of d_N on the basis of an analysis of single gene.

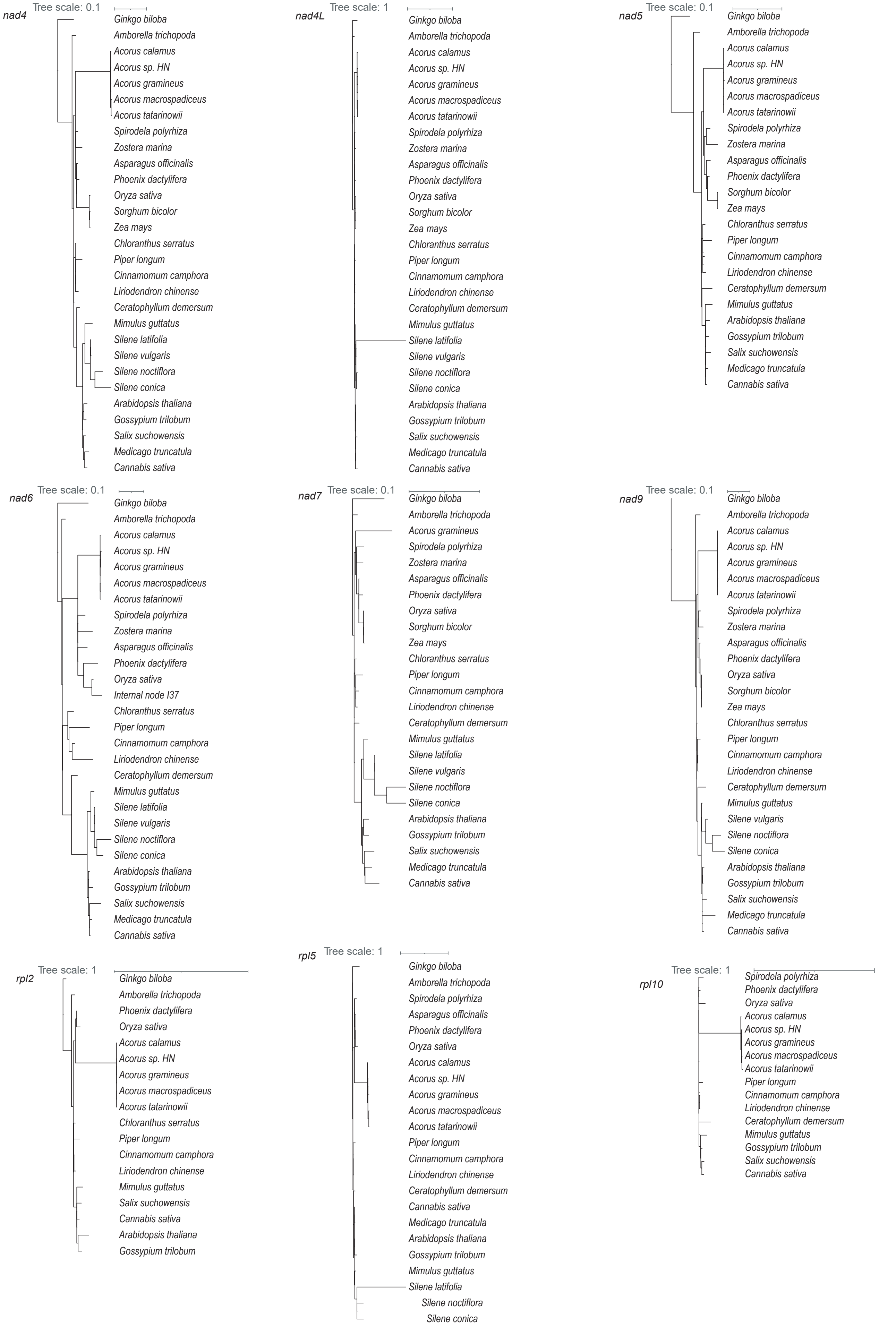

Supplementary Figure 53. Phylograms of non-synonymous nucleotide substitution per site (d_N) sequence divergence of *nad4*, *nad4L*, *nad5*, *nad6*, *nad7*, *nad9*, *rpl2*, *rpl5* and *rpl10* genes. Branch lengths are scaled to the value of d_N on the basis of an analysis of single gene.

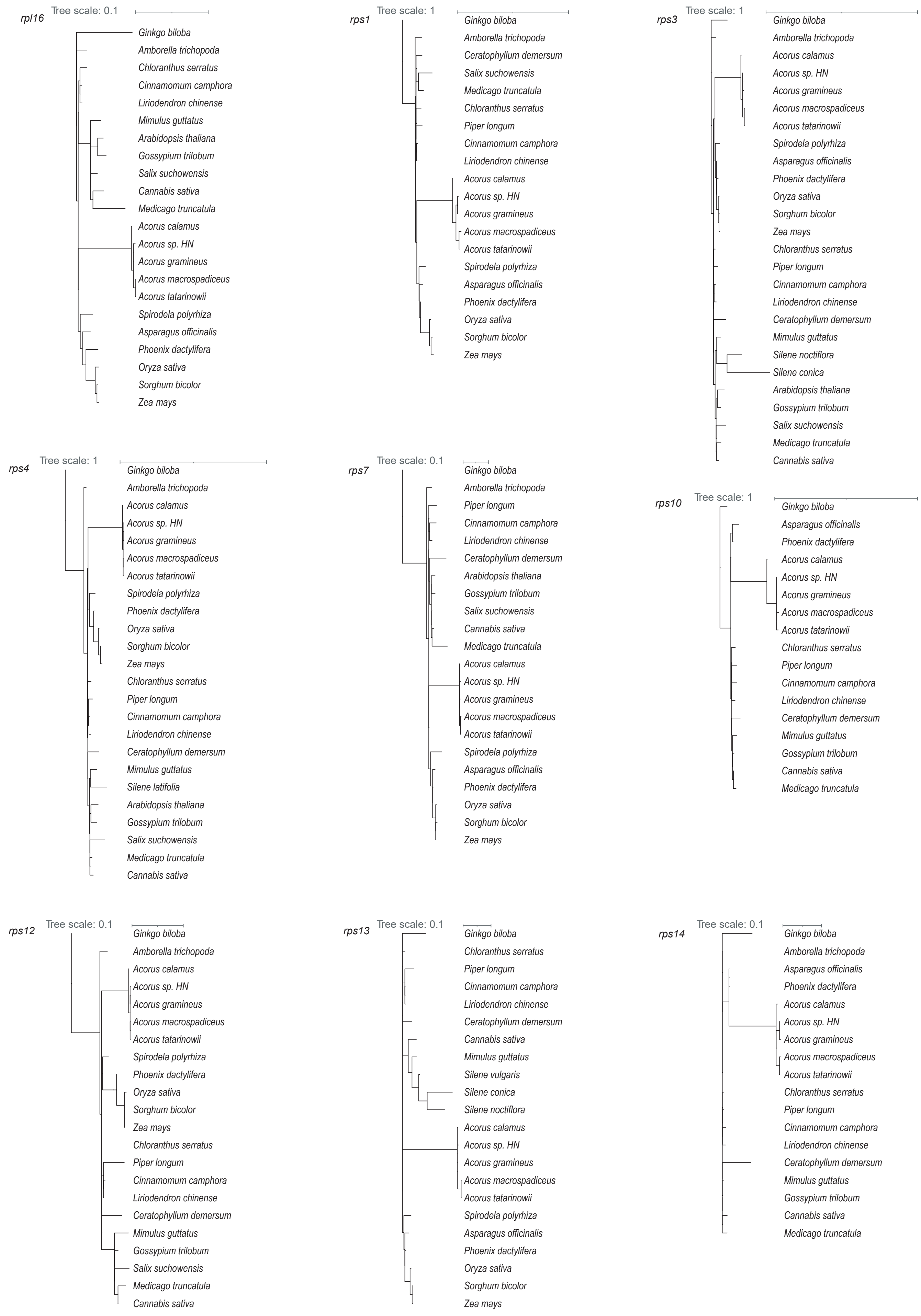

Supplementary Figure 54. Phylograms of non-synonymous nucleotide substitution per site (d_N) sequence divergence of *rpl16*, *rps1*, *rps3*, *rps7*, *rps10*, *rps12*, *rps13* and *rps14* genes. Branch lengths are scaled to the value of d_N on the basis of an analysis of single gene.

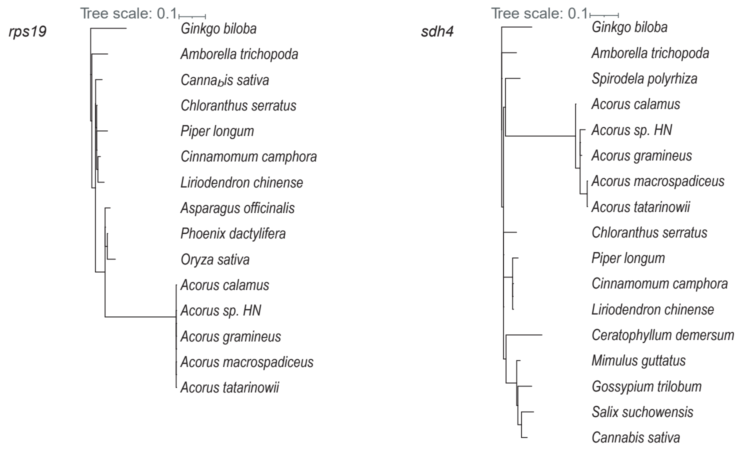

Supplementary Figure 55. Phylograms of non-synonymous nucleotide substitution per site (d_N) sequence divergence of *rps19* and *sdh4* genes. Branch lengths are scaled to the value of d_N on the basis of an analysis of single gene.

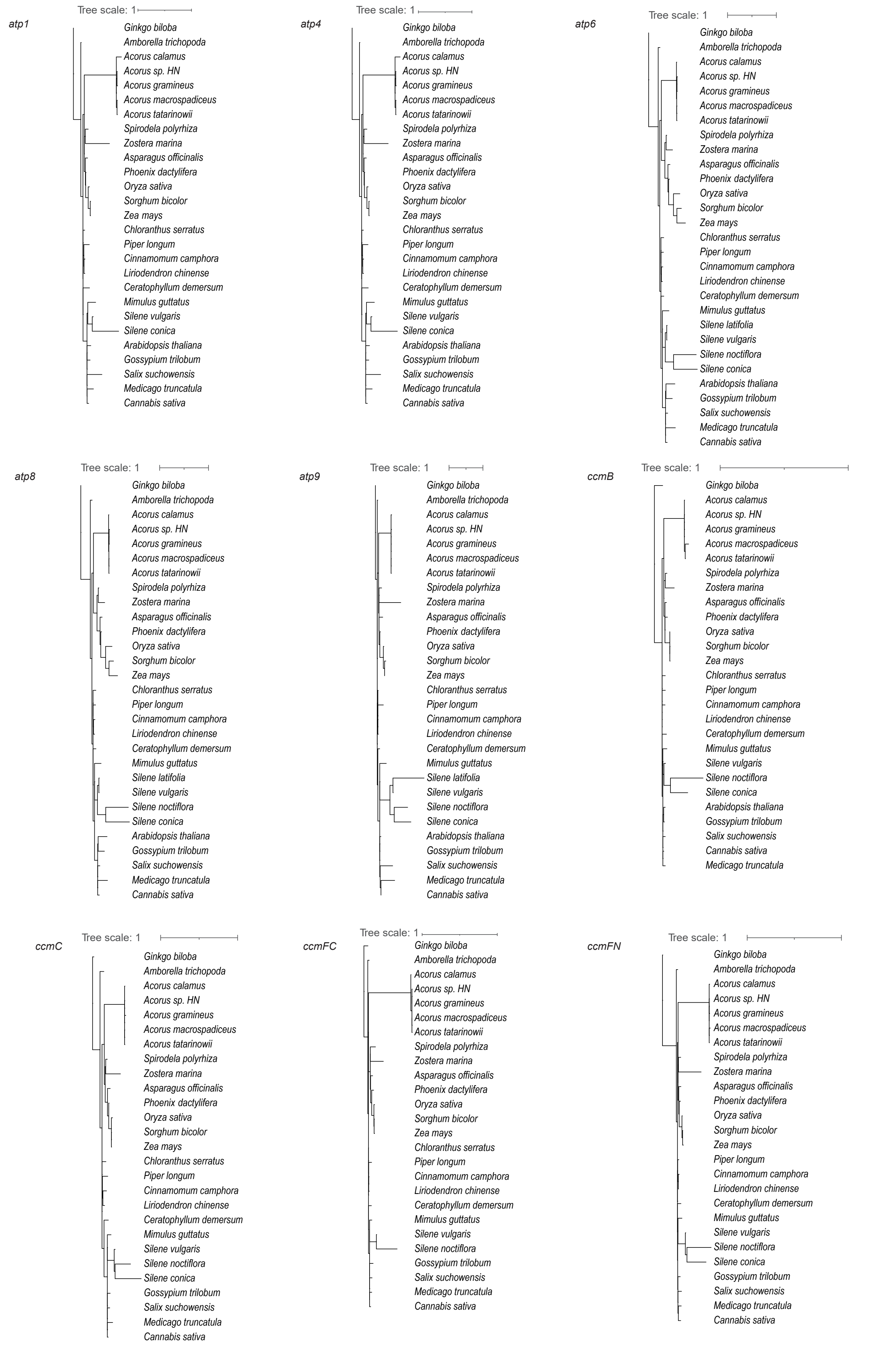

Supplementary Figure 56. Phylograms of synonymous nucleotide substitution per site (d_S) sequence divergence of *atp1*, *atp4*, *atp6*, *atp8*, *atp9*, *ccmB*, *ccmC*, *ccmFC* and *ccmFN* genes. Branch lengths are scaled to the value of d_S on the basis of an analysis of single gene.

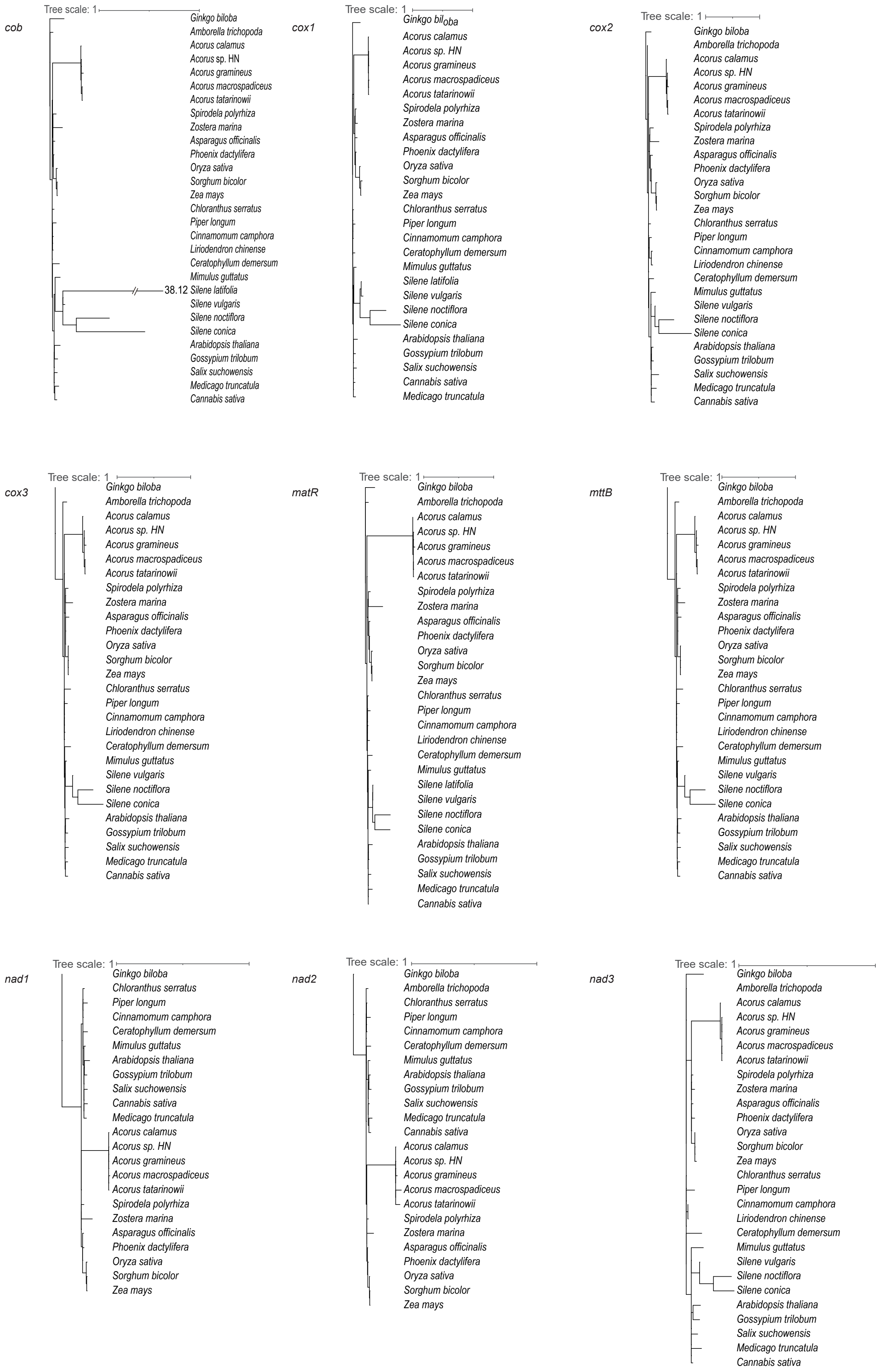

Supplementary Figure 57. Phylograms of synonymous nucleotide substitution per site (d_S) sequence divergence of *cob*, *cox1*, *cox2*, *cox3*, *matR*, *mttB*, *nad1*, *nad2* and *nad3* genes. Branch lengths are scaled to the value of d_S on the basis of an analysis of single gene.

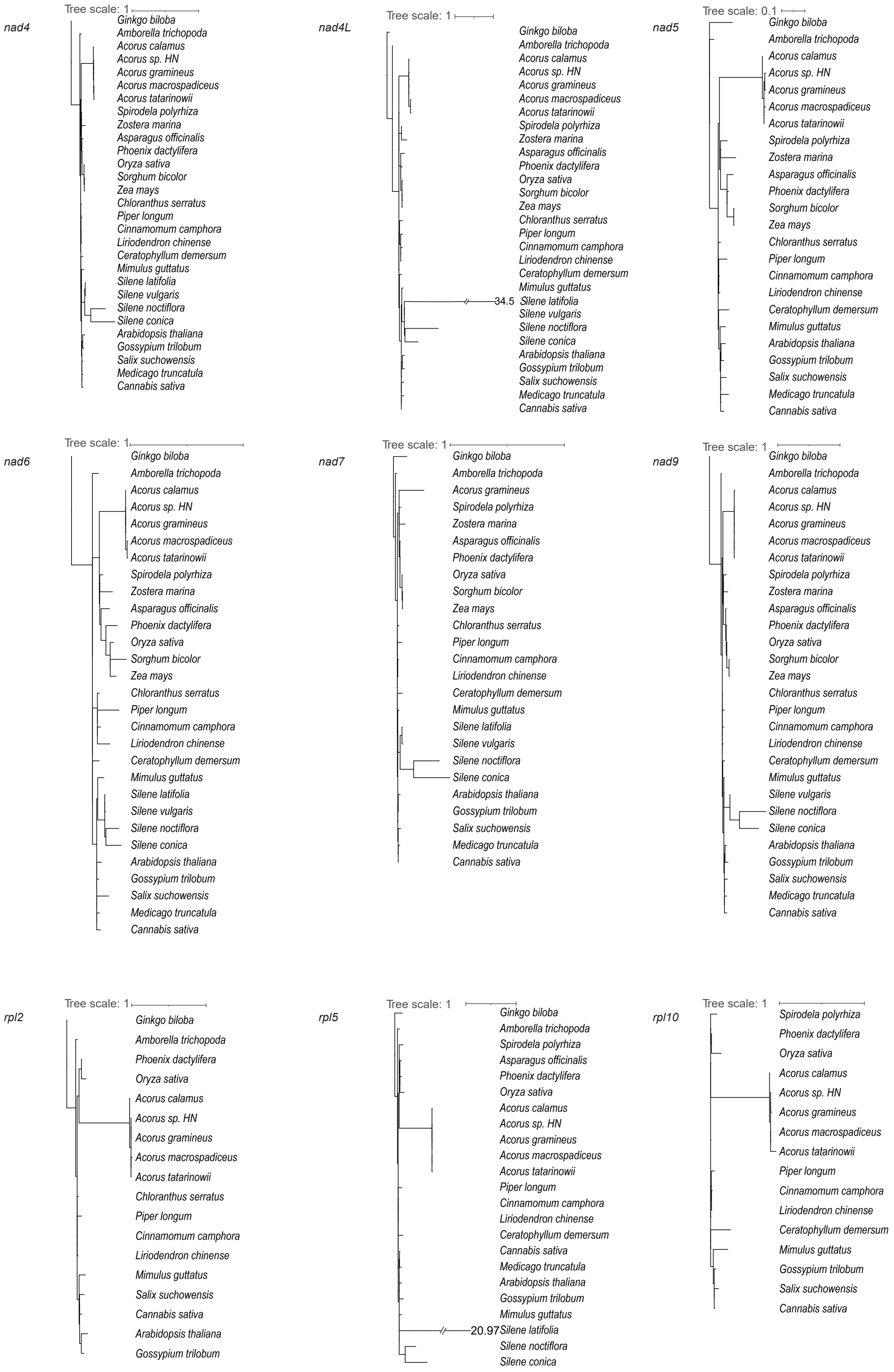

Supplementary Figure 58. Phylograms of synonymous nucleotide substitution per site (d_S) sequence divergence of *nad4*, *nad4L*, *nad5*, *nad6*, *nad7*, *nad9*, *rpl2*, *rpl5* and *rpl10* genes. Branch lengths are scaled to the value of d_S on the basis of an analysis of single gene.

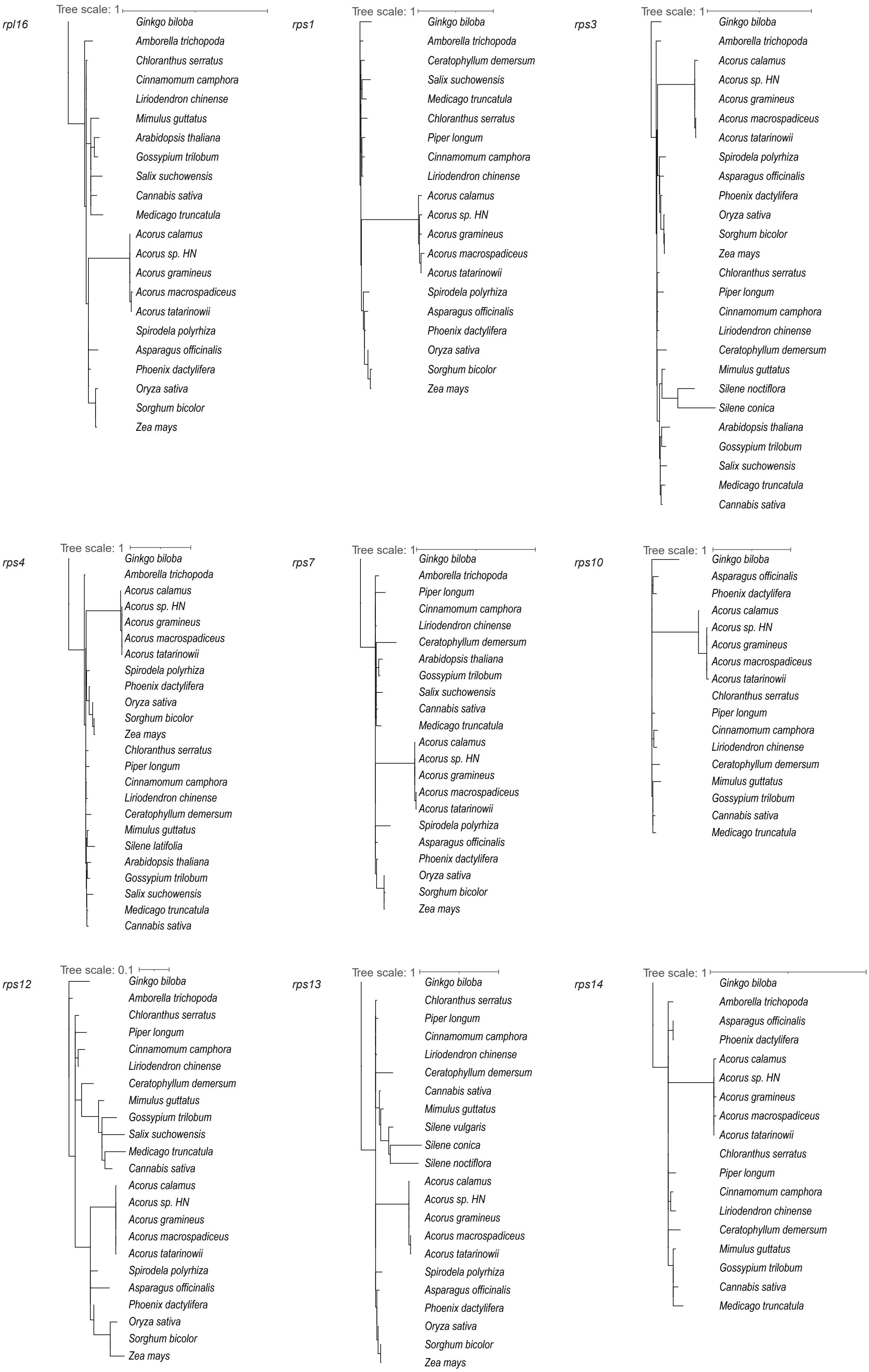

Supplementary Figure 59. Phylograms of synonymous nucleotide substitution per site (d_S) sequence divergence of *rpl16*, *rps1*, *rps3*, *rps7*, *rps10*, *rps12*, *rps13* and *rps14* genes. Branch lengths are scaled to the value of d_S on the basis of an analysis of single gene.

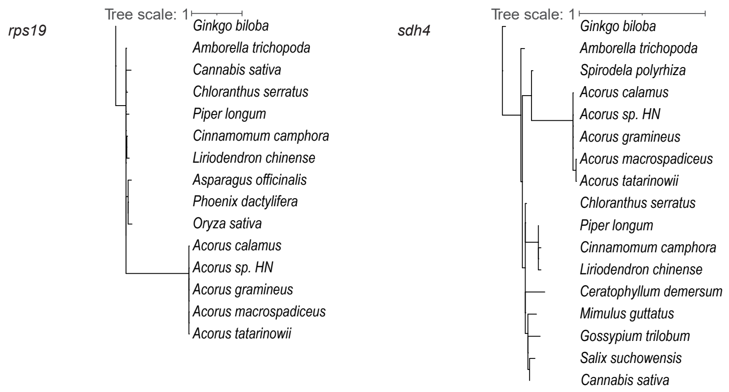

Supplementary Figure 60. Phylograms of synonymous nucleotide substitution per site (d_S) sequence divergence of *rps19* and *sdh4* genes. Branch lengths are scaled to the value of d_S on the basis of an analysis of single gene.

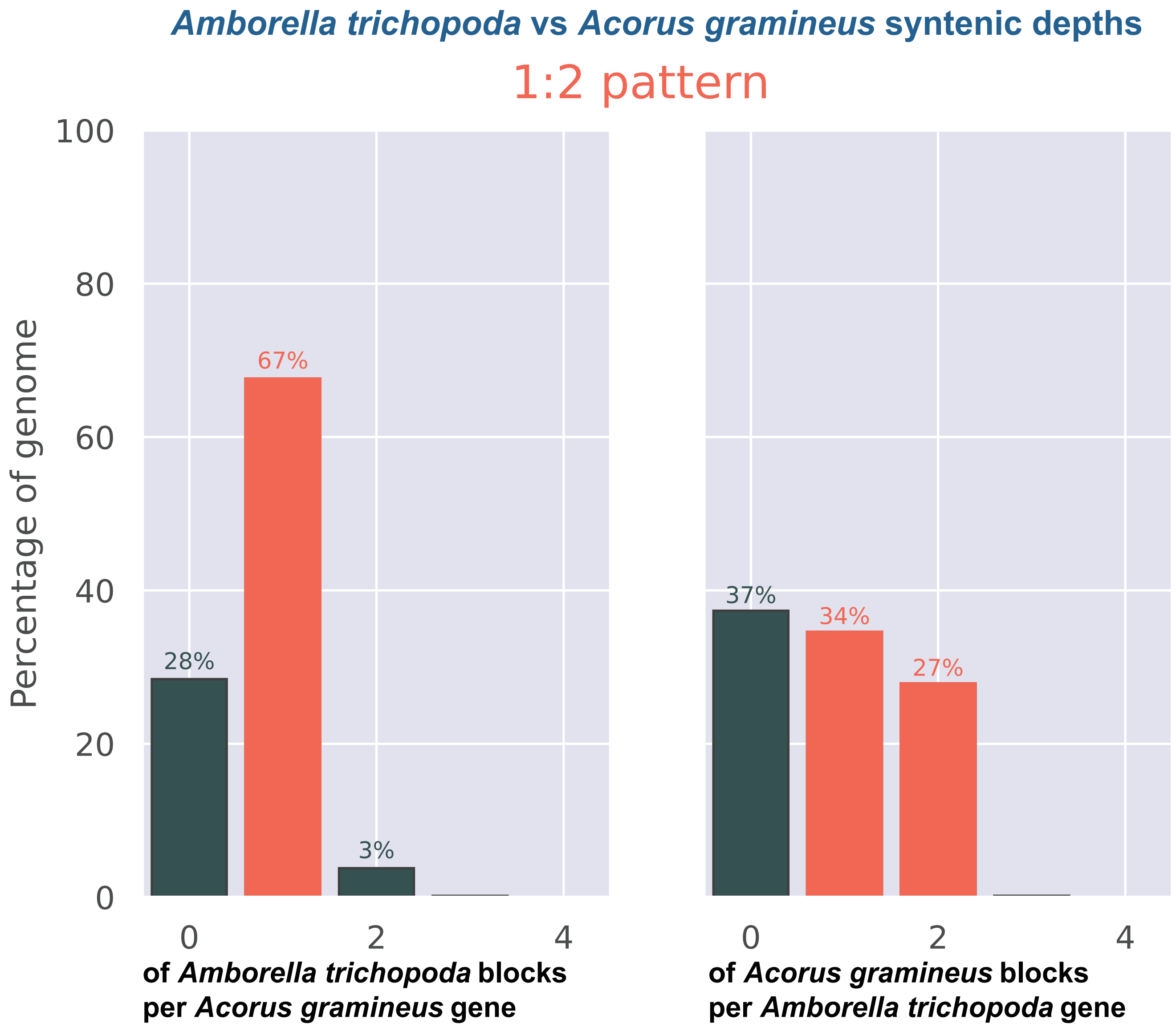

Supplementary Figure 61. The distribution of syntenic depths between *Amborella trichopoda* and *Acorus gramineus*.

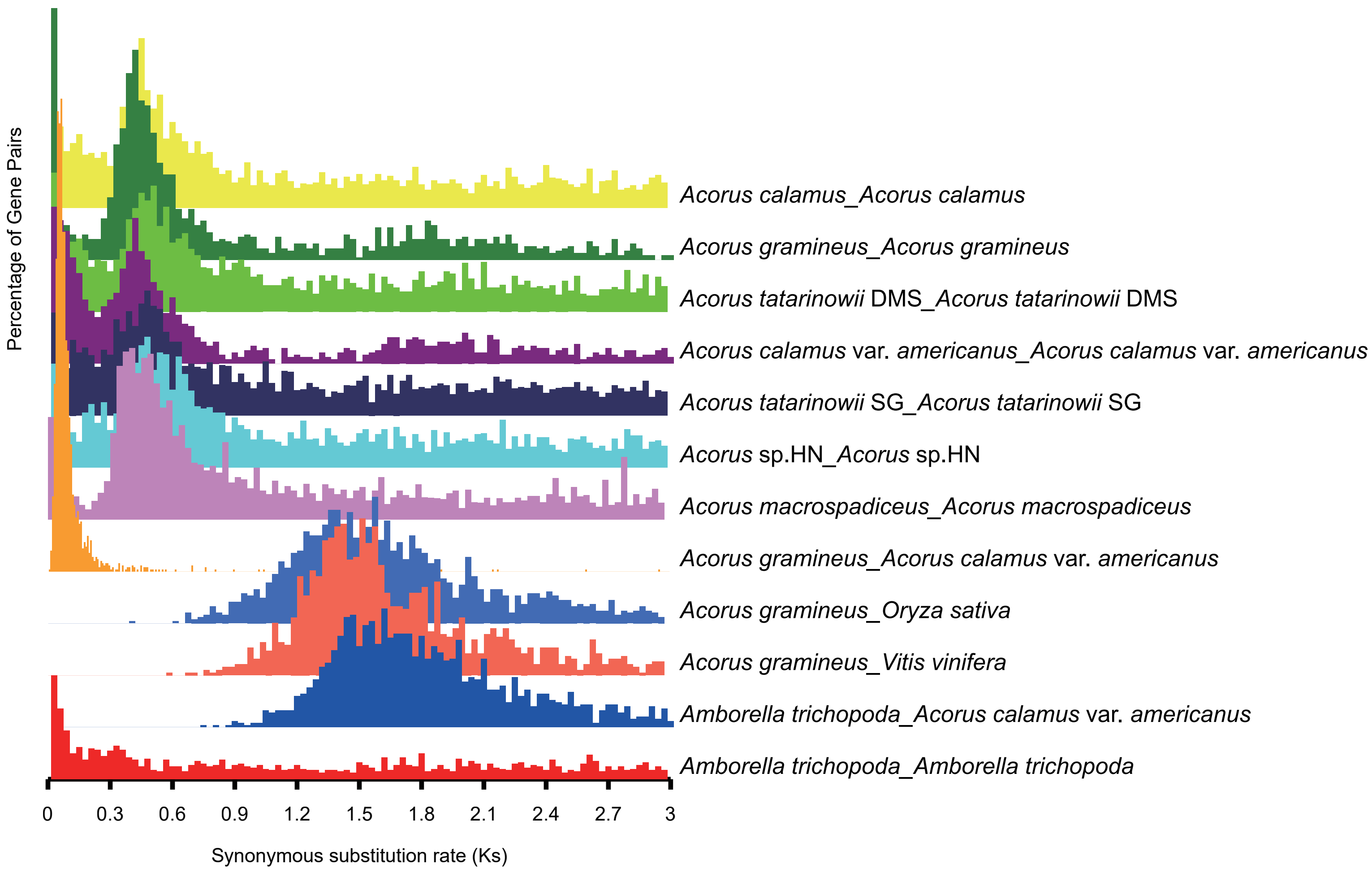

Supplementary Figure 62. Distribution of synonymous substitution rates (*Ks*) of the species of *Acorus* spp.

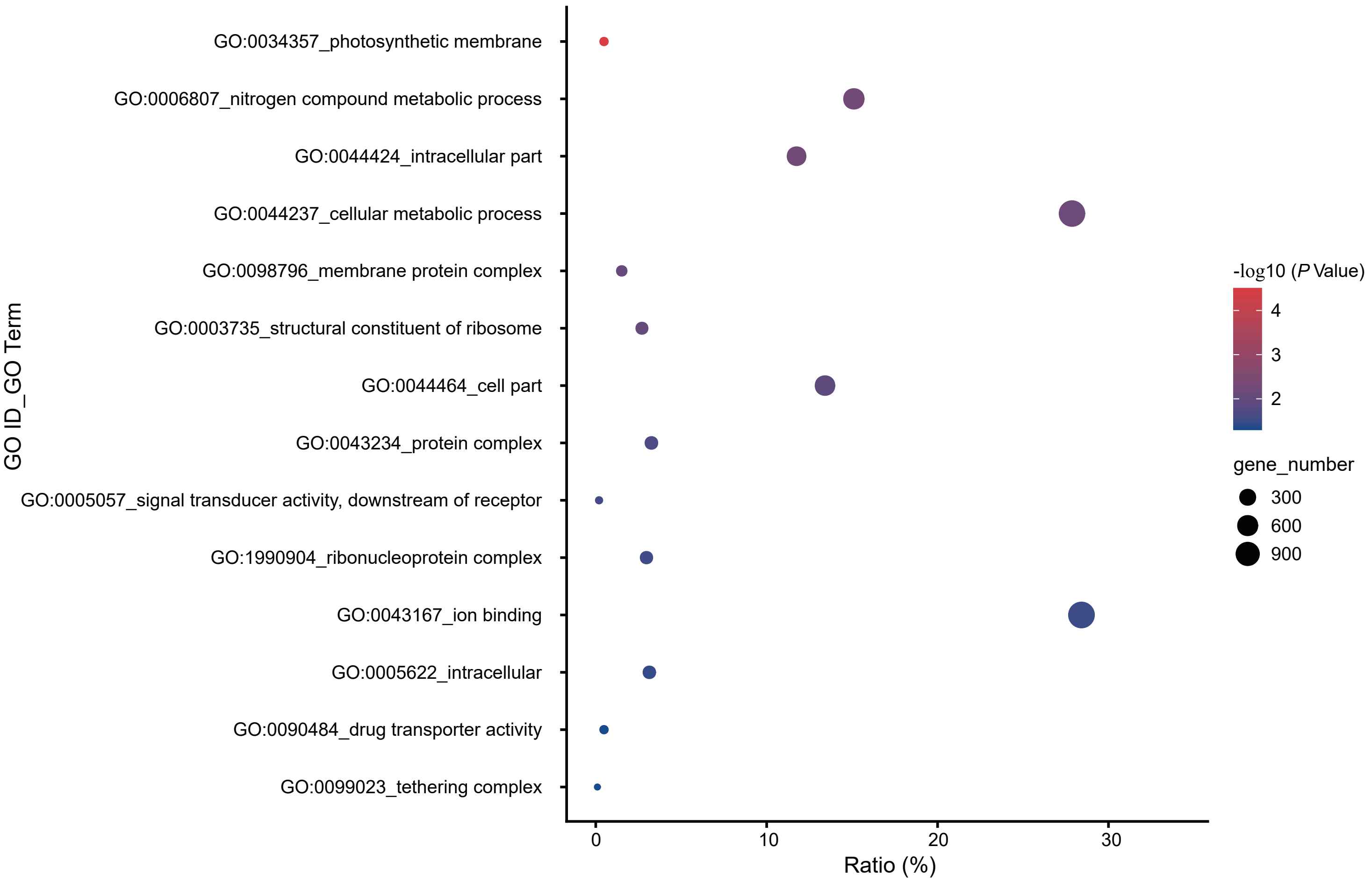

Supplementary Figure 63. Bubble plot show GO enrichment analysis of WGD retained genes. The statistical method is $\chi 2$ test. The *p*-values obtained from significance tests are generally considered to have significant statistical differences when $p<0.05$. Ratio is equal total number of corresponding enriched genes divided by the total gene number .

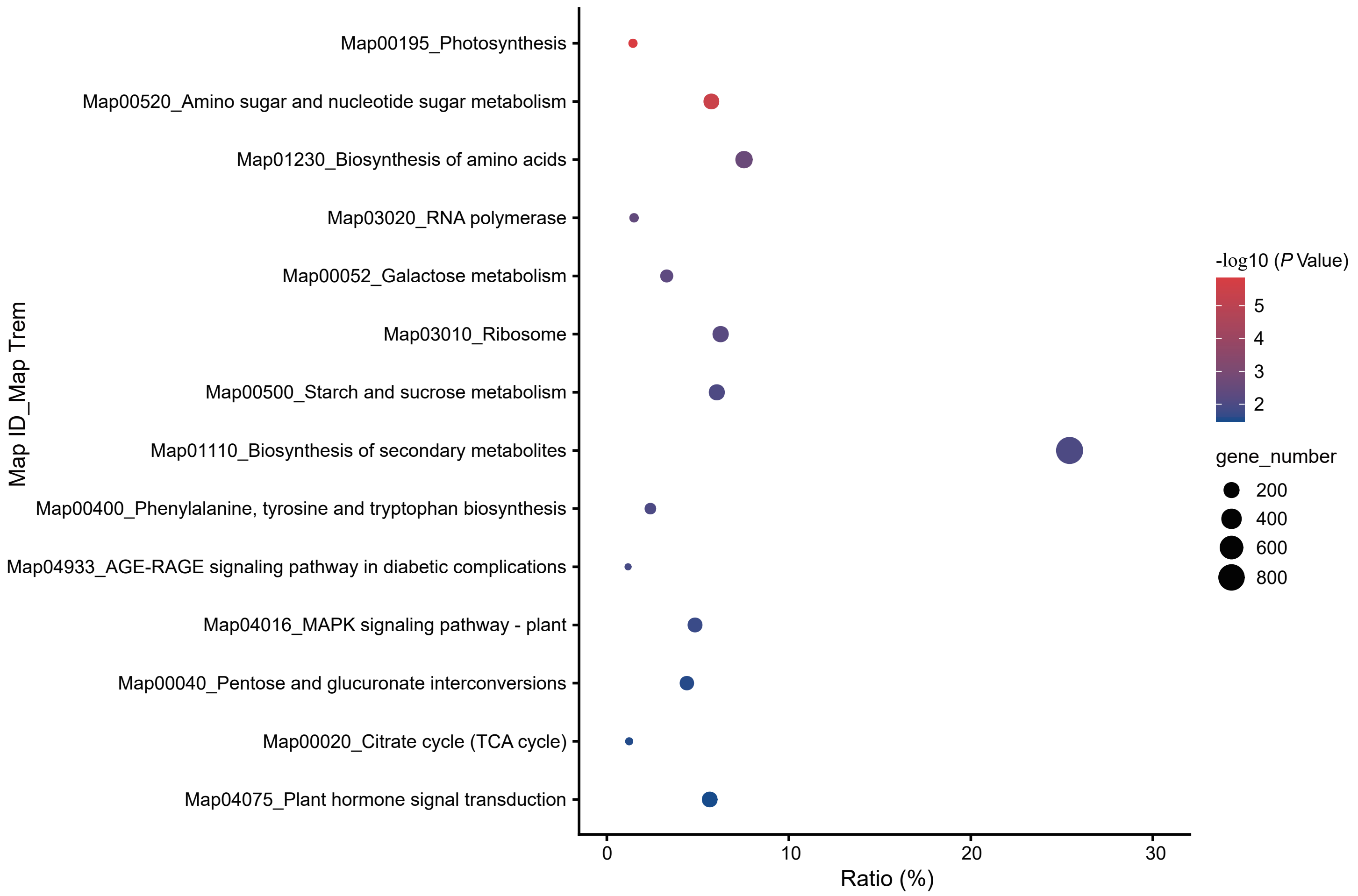

Supplementary Figure 64. Bubble plot show KEGG enrichment analysis of WGD retained genes. The statistical method is χ^2 test. The *p*-values obtained from significance tests are generally considered to have significant statistical differences when $p<0.05$. Ratio is equal total number of corresponding enriched genes divided by the total gene number .

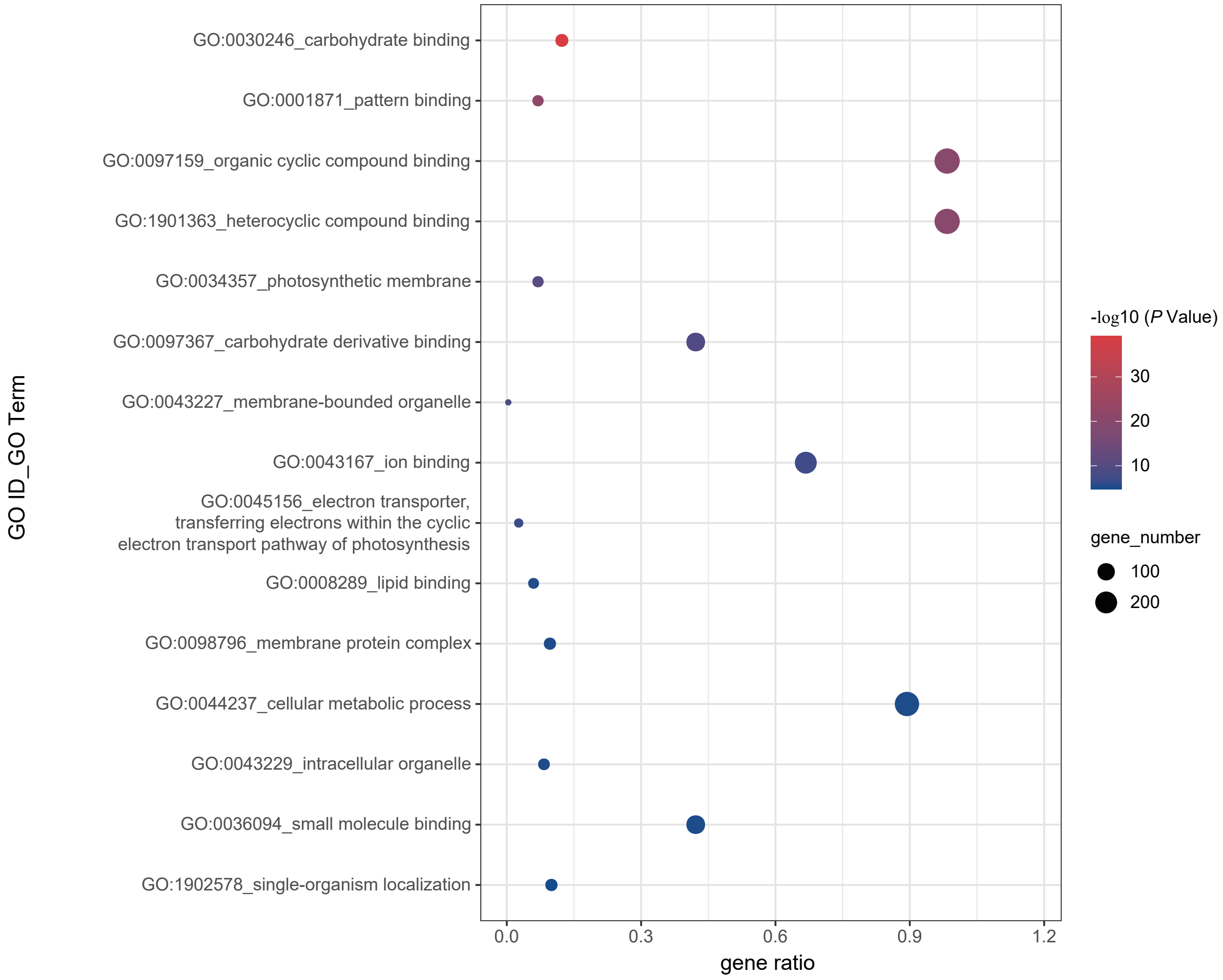

Supplementary Figure 65. Bubble plot show Gene Ontology (GO) studies based on the 84 expanded gene families.

The statistical methods are $\chi 2$ test. AdjustedPv is a corrected *p*-value that is obtained by performing false discovery rate (FDR) testing on *p*-values. AdjustedPv obtained from significance tests are generally considered to have significant statistical differences when AdjustedPv<0.05; Gene ratio is equal to the number of corresponding enriched genes divided by the total gene number.

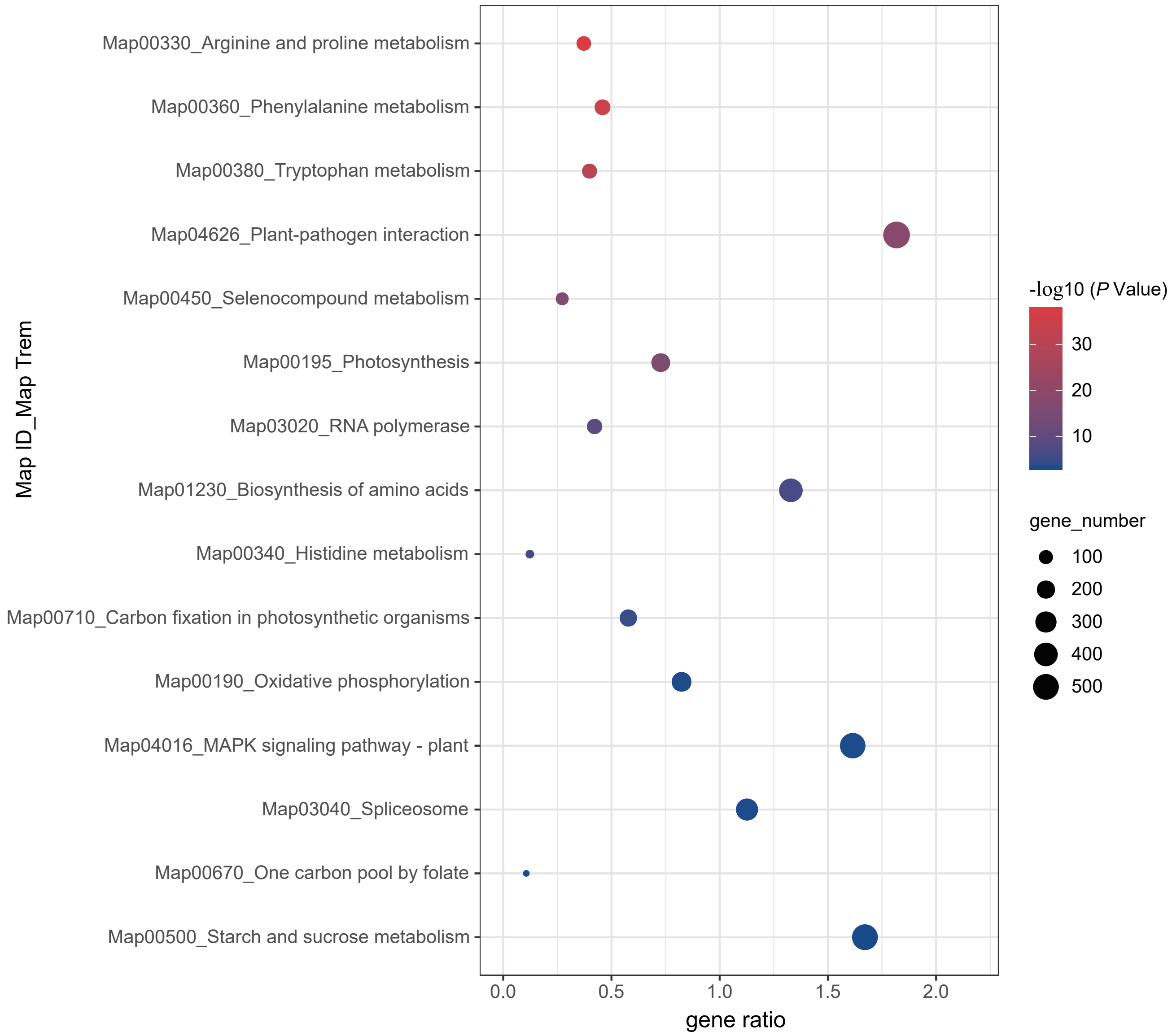

Supplementary Figure 66. Bubble plot show KEGG enrichment analysis based on the 84 expanded gene families.

The statistical methods are χ2 test. AdjustedPv is a corrected *p*-value that is obtained by performing false discovery rate (FDR) testing on *p*-values. AdjustedPv obtained from significance tests are generally considered to have significant statistical differences when AdjustedPv<0.05; Gene ratio is equal to the number of corresponding enriched genes divided by the total gene number.

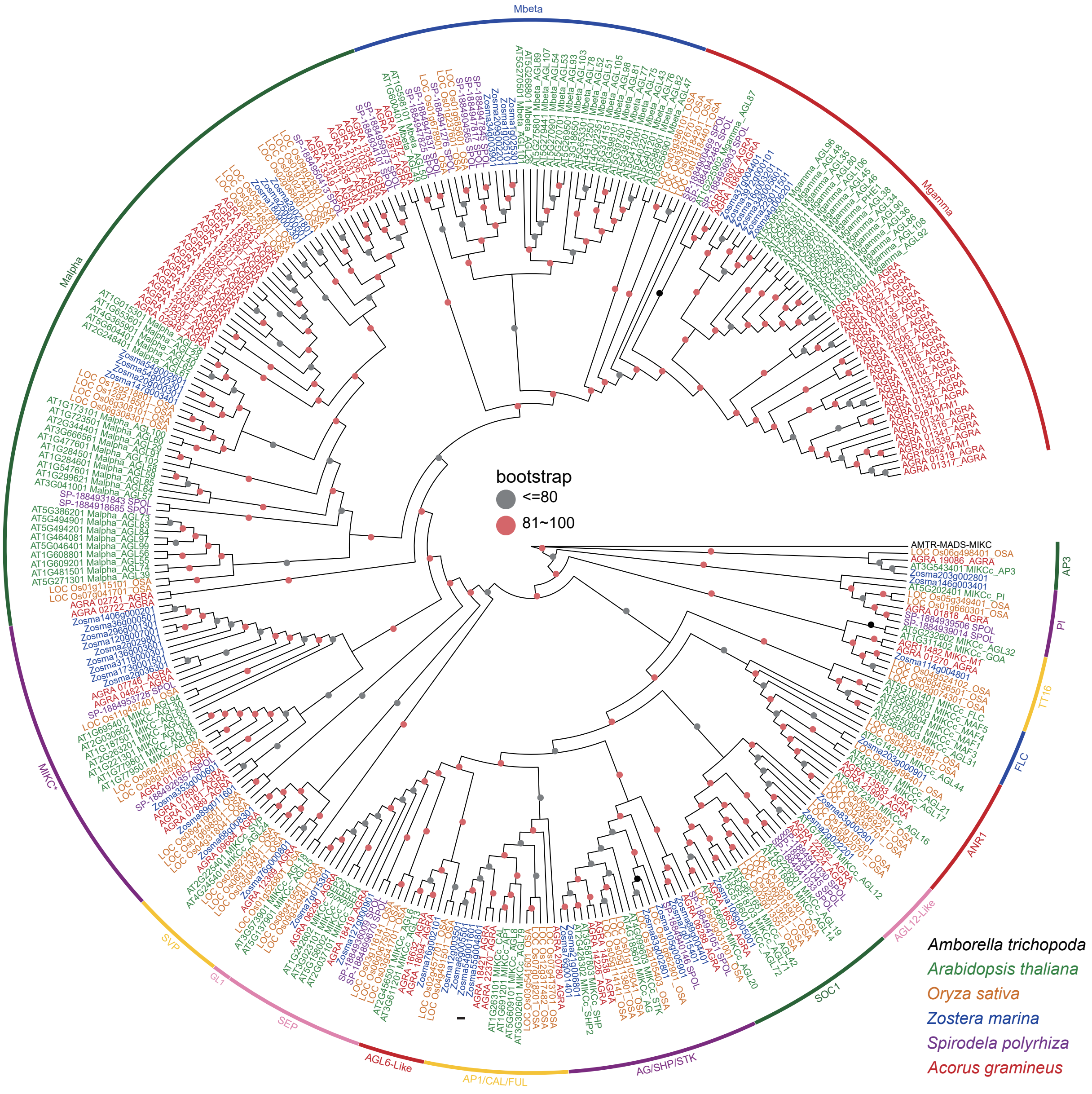

Supplementary Figure 67. The tree of MADS box superfamily in *Acorus gramineus* comparison with *Arabidopsis thaliana*, *Zostera marina*, *Spirodela polyrhiza* and *Oryza sativa*.

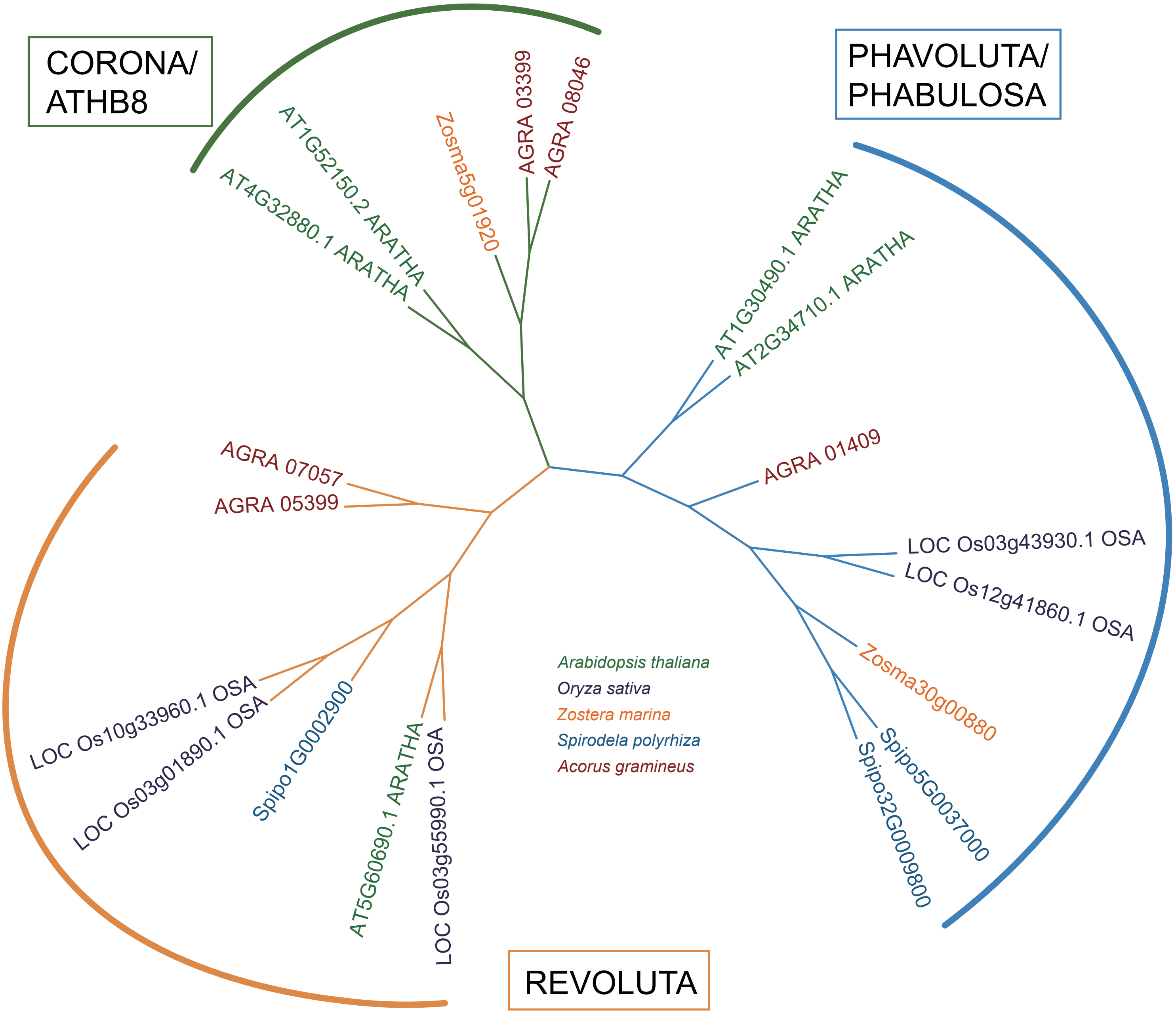

Supplementary Figure 68. The tree of HD-ZIP-III TF in *Acorus gramineus* comparison with *Arabidopsis thaliana*, *Zostera marina*, *Spirodela polyrhiza* and *Oryza sativa*.

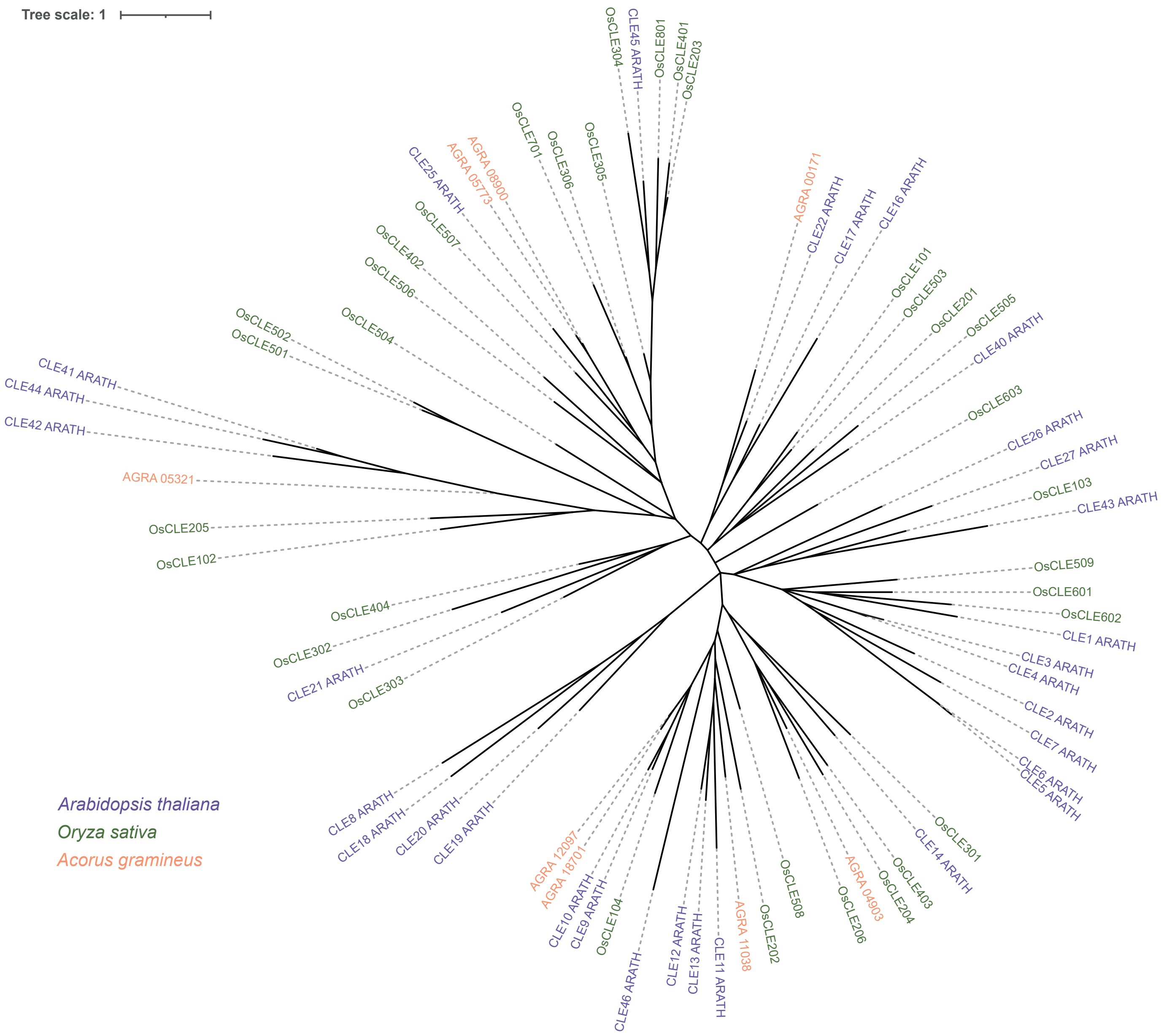

Supplementary Figure 69. Phylogenetic tree of the CLE in *Acorus gramineus*, *Arabidopsis thaliana* and *Oryza sativa*.

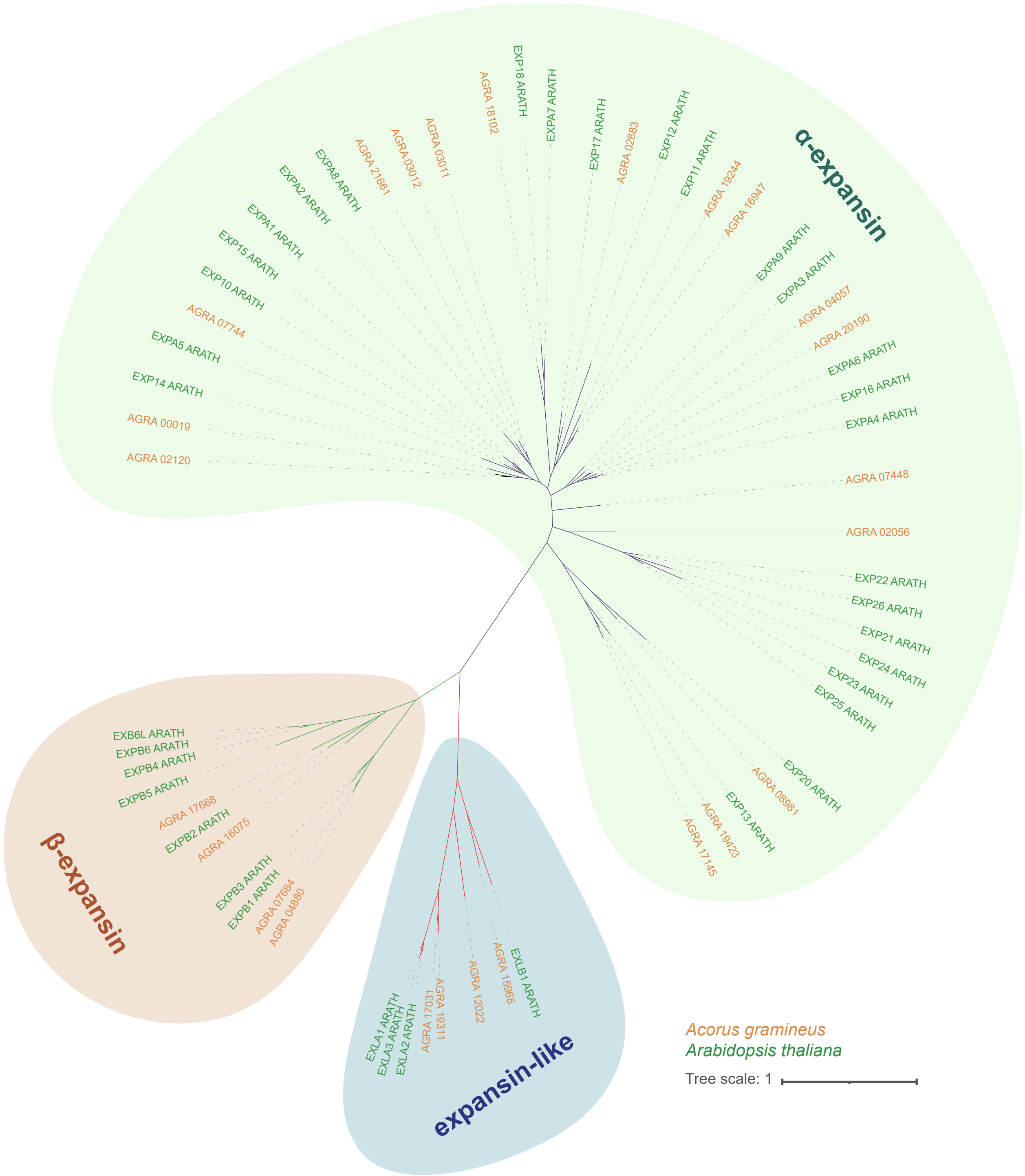

Supplementary Figure 70. Phylogenetic tree of the expansin family in *Acorus gramineus* and *Arabidopsis thaliana*.

Supplementary Table 1. K-mer analysis of *Acorus gramineus* with GenomeScope2.0 (K=17).

Property	Min	Max
Homozygous (aa) %	98.9705	99.0719
Heterozygous (ab) %	0.928097	1.02954
Genome Haploid Length (bp)	400,255,273	402,052,135
Genome Repeat Length (bp)	241,415,645	242,499,430
Genome Unique Length (bp)	158,839,628	159,552,705
Model Fit %	48.5379	97.6919
Read Error Rate %	0.179711	0.179711
Genome Unique Length (bp)	158,839,628	159,552,705
Model Fit %	48.5379	97.6919
Read Error Rate %	0.179711	0.179711

Supplementary Table 2. Statistic of *Acorus gramineus* different version genome assembly.

	Gapfree Psudo_Chromosome genome (version 2.0)		Psudo_Chromosome genome (version 1.0)		Contig	
	Length (bp)	Number	Length (bp)	Number	Length (bp)	Number
max length:	47,461,062		45,034,702		34,001,685	
N90	22,389,215	11	20,746,030	11	2,351,775	36
N80	24,702,493	9	24,196,211	9	4,184,027	24
N70	29,262,057	8	28,252,412	8	6,541,777	17
N60	35,341,745	6	34,221,588	6	10,031,507	12
N50	36,523,515	5	36,056,323	5	11,953,216	9
N40	39,396,831	4	36,304,364	4	16,000,951	6
N30	39,863,050	3	36,898,856	3	22,456,753	4
N20	43,804,742	2	38,687,477	2	28,150,784	3
N10	47,461,062	1	45,034,702	1	33,970,948	2
Total length	399,844,358		375,335,914		375,269,914	
Total_N	0		66,041		41	
GC (%)	39.88		39.5		39.5	

Supplementary Table 3. Validation of *Acorus gramineus* different version and *Acorus tatarinowii* genome assembly using BUSCO method.

Type	*Acorus gramineus* Gapfree Psudo_Chromosome genome	*Acorus gramineus* Psudo_Chromosome genome (version 1.0)	*Acorus_tatarinowii* genome
Complete BUSCOs (C)	96.7%	94.8%	95.6%
Complete and single-copy BUSCOs (S)	92.4%	92.0%	87.9%
Complete and duplicated BUSCOs (D)	4.3%	2.8%	7.7%
Fragmented BUSCOs (F)	1.1%	0.7%	1.4%
Missing BUSCOs (M)	2.2%	4.5%	3.0%

Note:
BUSCO version is 5.3.2
The lineage dataset is: embryophyta_odb10 (Creation date: 2020-09-10, number of genomes: 50, number of BUSCOs: 1614)

Supplementary Table 4. Functional annotation of *Acorus gramineus* protein-coding genes.

Values	Number	Percentage
Total	23,207	100.00%
Nr-Annotated	21,543	92.83%
Swissprot-Annotated	18,178	78.33%
KEGG-Annotated	17,447	75.18%
COG-Annotated	16,985	73.19%
TrEMBL-Annotated	21,804	93.95%
Interpro-Annotated	22,231	95.79%
Overall	22,526	97.07%

Supplementary Table 5. Summery of repeat elements of *Acorus gramineus* genome.

Class	Repeat Size (bp)	Percent (%)
Repeat	200,221,221	50.07
Tandem Repeats	16,919,281	4.23
Transposable Elements (TE)	192,889,139	48.24
Class I Transposable Elements (TE)	19,529,255	4.88
Class II Transposable Elements (TE)	163,310,584	40.88
LTR	153,498,798	38.39
LTR/*Copia*	28,961,571	7.24
LTR/*Gypsy*	112,125,388	28.04
SINEs	272,911	0.07
LINEs	6,366,928	1.59

Supplementary Table 6. Transposable elements of *Acorus gramineus* genome.

Class	Type	SubType	Number of Elements	Length Occupied (bp)	Percentage of Sequence (%)
Class I Transposable Elements	TIR	Total	52,990	9,944,367	2.49
		CACTA	14,501	1,336,071	0.33
		Mutator	3,021	428,847	0.11
		Harbinger	2,520	383,035	0.10
		hAT	32,595	7,767,054	1.94
		Mariner	353	29,360	0.01
	unknown	Total	2,576	427,980	0.11
		unknown	2,576	427,980	0.11
	Helitron	Total	3,435	993,531	0.25
		Helitron	3,435	993,531	0.25
Class II Transposable Elements	LTR	Total	325,613	163,310,584	40.84
		Gypsy	171,201	112,125,388	28.04
		Copia	98,426	28,961,571	7.24
		unknown	55,986	22,223,625	5.56
	non-LTR	Total	1,591	159,534	0.04
		non-LTR	1,591	159,534	0.04

Supplementary Table 7. The list of 9 samples used for the *Acorus* interspecific analysis.

Smaple list	Short Name	Source	Voucher Number
Acorus calamus	ACOCAL	this study transcriptome	CP20200501GX,SCBG
Acorus calamus var. *americanus*	MTII	1KP	
Acorus gramineus	AGRAM	this study genome	JQP20200401GX,SCBG
Acorus gramineus	JQP	this study transcriptome	JQP20200401GX,SCBG
Acorus tatarinowii DMS	DMS	this study transcriptome	DMS20200301GX,SCBG
Acorus tatarinowii SG	SG	this study transcriptome	SG20200401GX,SCBG
Acorus macrospadiceus	SN	this study transcriptome	SN20210108WF,SCBG
Acorus sp.HN	HN	this study transcriptome	HN20200301GX,SCBG

Supplementary Table 8. Topologies of 38 mitochondrial single genes.

Classify	Trees	Place	Bootstrap Values		
			100%	≥80%	≥75%
Monocot	10	Alismatales	*atp1,matR,nad4,rps3,sdh4*	*ccmB*	\
		Poales	*cob*		\
		Acorales	*atp4*	*nad1,rps12*	\
Eudicots	3	Brassicales	*rpl2*	\	\
		Eudicots basal	*ccmFC*	\	*cox2*
Magnoliids	1	Magnoliales	*rps4*	\	\
Ceratophyllales	1	*Ceratophyllum demersum*	*ccmFN*	\	\
Unclassified	23	\	\	\	\
Total	38	\	\	\	\

Supplementary Table 9. The syntenic blocks between genomic regions in *Acorus gramineus* vs *Amborella trichopoda* and *Acorus gramineus vs Acorus tatarinowii.*

A vs B	*Acorus gramineus* vs *Amborella trichopoda*	*Acorus gramineus* vs *Acorus tatarinowii*
of Syntenic Blocks (Scaffold)	271	35
Average Syntenic Gene Pairs per Block	13.4207	334.4286
of Syntenic Gene Pairs	3,637	11,705
Mean Block Length	722,634.8930/1,527,309.4465	11,403,763.1714/10,717,701.8000

Supplementary Table 10. GO enrichment analysis of WGD retained genes.

GO ID GO Term	*p* -value	Gene Number	Gene Ratio
GO:0034357_photosynthetic membrane	3.15E-05	20	0.00482509
GO:0006807_nitrogen compound metabolic process	5.20E-03	626	0.151025
GO:0044424_intracellular part	0.005294692	487	0.117491
GO:0044237_cellular metabolic process	0.006613819	1,155	0.278649
GO:0098796_membrane protein complex	0.008132776	63	0.015199
GO:0003735_structural constituent of ribosome	0.008982998	112	0.0270205
GO:0044464_cell part	0.011228928	556	0.134138
GO:0043234_protein complex	0.019037666	135	0.0325694
GO:0005057_signal transducer activity, downstream of receptor	0.023706787	8	0.00193004
GO:1990904_ribonucleoprotein complex	0.027972465	123	0.0296743
GO:0043167_ion binding	0.030522325	1,178	0.284198
GO:0005622_intracellular	0.037737106	130	0.0313631
GO:0090484_drug transporter activity	0.046663275	20	0.00482509
GO:0099023_tethering complex	0.049454791	4	0.000965018

Note: The statistical methods are χ^2 test. The *p* -values obtained from significance tests are generally considered to have significant statistical differences when p <0.05; Gene ratio is equal to the number of corresponding enriched genes divided by the total gene number .

Supplementary Table 11. KEGG enrichment analysis of WGD retained genes.

KEGG map	*p* -value	Gene Number	Gene Ratio
map00195_Photosynthesis	1.46E-06	48	0.0143584
map00520_Amino sugar and nucleotide sugar metabolism	3.45E-06	192	0.0574334
map01230_Biosynthesis of amino acids	0.002330379	252	0.0753814
map03020_RNA polymerase	0.003652949	50	0.0149566
map00052_Galactose metabolism	0.004762776	110	0.0329046
map03010_Ribosome	0.00611127	209	0.0625187
map00500_Starch and sucrose metabolism	0.0090188	202	0.0604248
map01110_Biosynthesis of secondary metabolites	0.009564747	850	0.254263
map00400_Phenylalanine, tyrosine and tryptophan biosynthesis	0.009765688	80	0.0239306
map04933_AGE-RAGE signaling pathway in diabetic complications	0.011960749	39	0.0116662
map04016_MAPK signaling pathway - plant	0.01847963	162	0.0484595
map00040_Pentose and glucuronate interconversions	0.024550887	147	0.0439725
map00020_Citrate cycle (TCA cycle)	0.024989106	41	0.0122644
map04075_Plant hormone signal transduction	0.033060716	189	0.056536

Note： The statistical methods are χ^2 test. The *p* -values obtained from significance tests are generally considered to have significant statistical differences when $p < 0.05$; Gene ratio is equal to the number of corresponding enriched genes divided by the total gene number.

Supplementary Table 12. The list of 14 species used for comparative genomic analysis in this study.

Species	Short Name	DataBase	Version
Amborella trichopoda	ATR	genome.jgi.doe.gov/PhytozomeV13	Atrichopoda_291_v1.0
Vitis vinifera	VVIN	genome.jgi.doe.gov/PhytozomeV12	Vvinifera_145_Genoscope.12X
Arabidopsis thaliana	ATHA	genome.jgi.doe.gov/PhytozomeV12	Athaliana_167_TAIR10
Acorus gramineus	AGRAM	this study	
Spirodela polyrhiza	SPOL	genome.jgi.doe.gov/PhytozomeV13	Spirodela polyrhiza v2
Zostera marina	ZMARI	genome.jgi.doe.gov/PhytozomeV13	Zostera marina v3.1
Asparagus officinalis	AOFF	genome.jgi.doe.gov/PhytozomeV13	Asparagus officinalis V1.1
Phalaenopsis equestris	PEQU	https://www.ncbi.nlm.nih.gov/	GCF_001263595.1_ASM126359v1
Ananas comosus	ACOM	genome.jgi.doe.gov/PhytozomeV13	Ananas comosus v3
Sorghum bicolor	SBI	genome.jgi.doe.gov/PhytozomeV12	Sorghum bicolor v3.1.1
Oryza sativa	OSA	genome.jgi.doe.gov/PhytozomeV13	Osativa_323_v7.0
Phoenix dactylifera	PDAC	https://www.ncbi.nlm.nih.gov/	GCF_000413155.1
Elaeis guineensis	EGU	https://www.ncbi.nlm.nih.gov/	EG5
Musa acuminata	MAC	genome.jgi.doe.gov/PhytozomeV12	Macuminata_304_v1

Supplementary Table 13. WRKY number of different species.

Class	I	IIa+b	IIc	IId+e	III	Total
Acorus gramineus	10	13	16	10	11	60
Spirodela polyrhiza	10	9	8	12	5	44
Zostera marina	9	9	8	12	6	44
Oryza sativa	12	12	15	17	39	95
Arabidopsis thaliana	13	11	17	14	15	70

Supplementary Table 14. Numbers of single domain R-genes in different plant species as compared with *Acorus gramineus.*

Species	NB	LRR	TIR	CC	RPW8	BED
Amborella trichopoda	109	351	17	1382	1	2
Vitis vinifera	314	770	27	1581	11	7
Arabidopsis thaliana	168	592	132	2211	11	8
Acorus gramineus	99	385	2	1749	1	45
Spirodela polyrhiza	22	253	0	1138	0	6
Zostera marina	44	279	0	1356	0	8
Asparagus officinalis	42	334	0	1809	0	6
Phalaenopsis equestris	48	258	1	1357	1	7
Ananas comosus	170	471	1	1883	3	4
Sorghum bicolor	340	660	2	2176	0	8
Oryza sativa	513	824	1	2949	0	22
Phoenix dactylifera	169	529	2	2120	1	15
Elaeis guineensis	156	227	1	2000	0	10
Musa acuminata	109	636	1	2381	1	15

Supplementary Table 15. Numbers of multiple domain R-genes in different plant species.

Species	NB-LRR	TIR-NB-LRR	CC-NB-LRR	RPW8-NB-LRR	BED-NB-LRR	TIR-NB
Amborella trichopoda	40	9	5	1	0	10
Vitis vinifera	215	15	37	5	0	19
Arabidopsis thaliana	125	83	20	5	0	97
Acorus gramineus	71	0	20	1	0	0
Spirodela polyrhiza	14	0	3	0	0	0
Zostera marina	15	0	0	0	0	0
Asparagus officinalis	19	0	4	0	0	0
Phalaenopsis equestris	12	0	4	1	0	0
Ananas comosus	116	0	37	1	0	0
Sorghum bicolor	198	0	29	0	0	0
Oryza sativa	258	0	48	0	2	0
Phoenix dactylifera	110	0	19	1	0	0
Elaeis guineensis	85	0	25	0	0	0
Musa acuminata	81	0	23	1	0	0

Supplementary Table 16. Number of LHCA/B proteins genes of *Acorus gramineus* and *Arabidopsis thaliana* .

LHC		*Acorus gramineus*	*Arabidopsis thaliana*
LHCA	LHCA1	1	1
	LHCA2	1	1
	LHCA3	2	1
	LHCA4	1	1
	LHCA5	1	1
	LHCA6	1	1
	Total	7	6
LHCB	LHCB1	9	5
	LHCB2	2	3
	LHCB3	1	1
	LHCB4	2	3
	LHCB5	1	1
	LHCB6	2	1
	LHCB7	1	1
	Total	18	15
Total		25	21

Supplementary Table 17. The number of TPS subfamily genes in the 5 species.

Species	a	b	c	e/f	g	Total Number
Arabidopsis thaliana	23	7	1	2	1	34
Oryza sativa	19	0	3	9	1	32
Zostera marina	0	0	1	1	0	2
Spirodela polyrhiza	0	0	0	2	2	4
Acorus gramineus	6	6	5	2	2	21

Supplementary references

1. Chikhi R, Medvedev P. Informed and automated k-mer size selection for genome assembly. *Bioinformatics* **30**, 31-37 (2014).
2. Doležel J*, et al.* Plant Genome size estimation by flow cytometry: Inter-laboratory Comparison. *Annals of Botany* **82**, 17-26 (1998).
3. Bharathan G, Lambert G, Galbraith D. Nuclear DNA content of monocotyledons and related taxa. *Am J Bot* **81**, 381-386 (1994).
4. van Berkum NL*, et al.* Hi-C: a method to study the three-dimensional architecture of genomes. *J Vis Exp* **39**, e1869 (2010).
5. Simao FA, Waterhouse RM, Ioannidis P, Kriventseva EV, Zdobnov EM. BUSCO: assessing genome assembly and annotation completeness with single-copy orthologs. *Bioinformatics* **31**, 3210-3212 (2015).
6. Cantarel BL*, et al.* MAKER: an easy-to-use annotation pipeline designed for emerging model organism genomes. *Genome Res* **18**, 188-196 (2008).
7. Boeckmann B*, et al.* The SWISS-PROT protein knowledgebase and its supplement TrEMBL in 2003. *Nucleic Acids Res* **31**, 365-370 (2003).
8. Kanehisa M*, et al.* KEGG for linking genomes to life and the environment. *Nucleic Acids Research* **36**, D480-D484 (2007).
9. Hunter S*, et al.* InterPro: the integrative protein signature database. *Nucleic Acids Res* **37**, D211-D215 (2009).
10. Hu J, Fan J, Sun Z, Liu S. NextPolish: a fast and efficient genome polishing tool for long-read assembly. *Bioinformatics* **36**, 2253-2255 (2020).
11. Li H. Minimap and miniasm: Fast mapping and de novo assembly for noisy long sequences. *Bioinformatics* **32**, 2103–2110 (2015).
12. Camacho C*, et al.* BLAST plus : architecture and applications. *Bmc Bioinformatics* **10**, 1-9 (2009).
13. Heng, Li, Durbin, Richard. Fast and accurate long-read alignment with Burrows–Wheeler transform. *Bioinformatics* **26**, 589–595 (2010).
14. Kim D, Langmead B, Salzberg SL. HISAT: a fast spliced aligner with low memory requirements. *Nat Methods* **12**, 357-360 (2015).
15. Bolger AM, Lohse M, Usadel B. Trimmomatic: a flexible trimmer for Illumina sequence data. *Bioinformatics* **30**, 2114-2120 (2014).
16. Neva C.*, et al.* Juicer Provides a One-click system for analyzing loop-resolution Hi-C Experiments. *Cell Systems* **3**, 95-98, (2016).
17. Dudchenko O*, et al.* De novo assembly of the *Aedes aegypti* genome using Hi-C yields chromosome-length scaffolds. *Science* **356**, 92-95 (2017).
18. Durand NC*, et al.* Juicebox Provides a visualization system for Hi-C contact maps with unlimited zoom. *Cell Syst* **3**, 99-101 (2016).
