# Supplementary material for: The genome of Acorus deciphers insights into early monocot evolution
Source: Nat Commun. 2023 Jun 20;14:3662. doi: 10.1038/s41467-023-38836-4 (PMC10281966; doi:10.1038/s41467-023-38836-4)
Supplement: Supplementary file 3 — Description of Additional Supplementary Files [file 41467_2023_38836_MOESM3_ESM.pdf]

Description of Additional Supplementary Files

Supplementary Data 1. Summary of sequencing data of *Acorus gramineus* genome.

Supplementary Data 2. Comparison of genome and gene characteristic of the *Acorus gramineus* genomes with other genomes.

Supplementary Data 3. The list of 15 species used for the evolutionary analysis.

Supplementary Data 4. The list of 223 species used for nuclear phylogenetic analyses in this study.

Supplementary Data 5. List of 135 taxa sampled for the chloroplast genomic dataset in this study.

Supplementary Data 6. List of 112 taxa sampled for the mitochondrial genomic dataset in this study.

Supplementary Data 7. Statistics of plant mitochondrial genomes downloaded from NCBI.

Supplementary Data 8. The d_N, d_S and d_N/d_S values of 38 mitochondrial single genes.

Supplementary Data 9. Ks distribution related to duplication events within representative genome and between genomes.

Supplementary Data 10. The fossil calibrations used in this study.

Supplementary Data 11. Gene Ontology (GO) studies based on the 84 expanded gene families.

Supplementary Data 12. KEGG enrichment analysis based on the 84 expanded gene families.

Supplementary Data 13. TFs number of different species.

Supplementary Data 14. The software used in this study.
