# Supplementary material for: The genome of Acorus deciphers insights into early monocot evolution
Source: Nat Commun. 2023 Jun 20;14:3662. doi: 10.1038/s41467-023-38836-4 (PMC10281966; doi:10.1038/s41467-023-38836-4)
Supplement: Supplementary file 18 — Reporting Summary [file 41467_2023_38836_MOESM18_ESM.pdf]

nature portfolio

Corresponding author(s): Huan Liu

Last updated by author(s): Apr 19, 2023

Reporting Summary

Nature Portfolio wishes to improve the reproducibility of the work that we publish. This form provides structure for consistency and transparency in reporting. For further information on Nature Portfolio policies, see our Editorial Policies and the Editorial Policy Checklist.

Statistics

For all statistical analyses, confirm that the following items are present in the figure legend, table legend, main text, or Methods section.

n/a	Confirmed	
☐	☒	The exact sample size (n) for each experimental group/condition, given as a discrete number and unit of measurement
☒	☐	A statement on whether measurements were taken from distinct samples or whether the same sample was measured repeatedly
☐	☒	The statistical test(s) used AND whether they are one- or two-sided<br>*Only common tests should be described solely by name; describe more complex techniques in the Methods section.*
☒	☐	A description of all covariates tested
☒	☐	A description of any assumptions or corrections, such as tests of normality and adjustment for multiple comparisons
☒	☐	A full description of the statistical parameters including central tendency (e.g. means) or other basic estimates (e.g. regression coefficient) AND variation (e.g. standard deviation) or associated estimates of uncertainty (e.g. confidence intervals)
☐	☒	For null hypothesis testing, the test statistic (e.g. F, t, r) with confidence intervals, effect sizes, degrees of freedom and P value noted<br>*Give P values as exact values whenever suitable.*
☐	☒	For Bayesian analysis, information on the choice of priors and Markov chain Monte Carlo settings
☒	☐	For hierarchical and complex designs, identification of the appropriate level for tests and full reporting of outcomes
☒	☐	Estimates of effect sizes (e.g. Cohen's d, Pearson's r), indicating how they were calculated

Our web collection on statistics for biologists contains articles on many of the points above.

Software and code

Policy information about availability of computer code

Data collection: No software was used for data collection

Data analysis: The list of Software used in this study are as follows:
Trimmomatic(v0.40),
Guppy(v4.0.11),
MinKNOW(v19.10.1),
SOAPfilter(v2.2),
GenomeScope(v2.0),
Canu(v2.0),
NextDenovo(v2.5.0),
Hifiasm(0.18.0-r465),
Nextpolish(v1.5.0),
Juicer(v1.7.6),
3d-dna(v180922),
Juicebox(v1.11.08),
Quickmerge(v0.3),
TGS-Gapcloser(1.1.1),
Repeats(Finder(v4.10.0),
cd-hit(v(4.8.1),
BUSCO(v5.3.2),
BLAST(v2.2.29),

Hisat2(v2.2.1),
Minimap2(v2.21-r1071),
BWA-MEM2(v2.2.1),
TRF(v4.10.0),
RepeatMasker(v4.0.5),
RepeatProteinMask(v4-0-7),
LTR_retriever(v2.8),
LTR_FINDER((v1.0.7),
RepeatModeler2(v2),
MITE-hunter(v2.2),
DANTE-Protein Domain Finder(v1.0),
RepeatExplorer((v0.9.7.8),
Augustus(v3.0.3),
GlimmerHMM(v3.0.1),
SNAP(version(11/29/2013),
TBLASTN (v2.2.18),
GeneWise(v2.2.0),
StringTie(v2.2.1),
PASA(v2.3.3),
Trinity(v2.6.6),
BLAT(v.34x12),
MAKER(v2),
InterProScan(v5.28-67.0),
iTAK(v1.6),
Transdecoder(v5.3.0),
OrthoMCL(v1.4),
MAFFT(v.7.471),
PAL2NAL(v14.1),
trimAL(v1.4.1),
IQTREE(v2.0.5),
ModelFinder(v2.0.5),
ASTRAL((v5.6.1),
OrthoFinder(v2.3.7),
NOVOPlasty(v4.3.1),
CpGAVAS2(v2),
Unicycler(v0.4.9),
PAML package (CODEMLprogram),
MCMCtree(v4.9),
MCScanX(v0.8),
MUSCLE((v3.8.31),
PhyML(v3.0),
Café(v4.1),
HMMER(v3.2.1),

For manuscripts utilizing custom algorithms or software that are central to the research but not yet described in published literature, software must be made available to editors and reviewers. We strongly encourage code deposition in a community repository (e.g. GitHub). See the Nature Portfolio guidelines for submitting code & software for further information.

Data

Policy information about availability of data

All manuscripts must include a data availability statement. This statement should provide the following information, where applicable:

- Accession codes, unique identifiers, or web links for publicly available datasets
- A description of any restrictions on data availability
- For clinical datasets or third party data, please ensure that the statement adheres to our policy

The whole genome sequence data and transcriptome sequence reported in this paper have been deposited in the Genome Warehouse in National Genomics Data Center, Beijing Institute of Genomics, Chinese Academy of Sciences/China National Center for Bioinformation, under accession number GWHCBFW00000000 that is publicly accessible at [https://ngdc.cncb.ac.cn/gsa/browse/CRA010626]. CNSA of CNGBdb with accession code CNP0002281[https://db.cngb.org/search/?q=CNP0002281]. The assembled genome and annotation have been deposited in the Genome Sequence Archive database under accession code GWHCBFW00000000 [https://ngdc.cncb.ac.cn/gwh/Assembly/37749/show].

Research involving human participants, their data, or biological material

Policy information about studies with human participants or human data. See also policy information about sex, gender (identity/presentation), and sexual orientation and race, ethnicity and racism.

Reporting on sex and gender	Not applicable
Reporting on race, ethnicity, or other socially relevant groupings	Not applicable

Population characteristics	Not applicable
Recruitment	Not applicable
Ethics oversight	Not applicable

Note that full information on the approval of the study protocol must also be provided in the manuscript.

Field-specific reporting

Please select the one below that is the best fit for your research. If you are not sure, read the appropriate sections before making your selection.

☒ Life sciences ☐ Behavioural & social sciences ☐ Ecological, evolutionary & environmental sciences

For a reference copy of the document with all sections, see nature.com/documents/nr-reporting-summary-flat.pdf

Life sciences study design

All studies must disclose on these points even when the disclosure is negative.

Sample size	One plant of Acorus gramineus genome was selected for genome sequencing and the same plant was used for RNA-seq of different organs for genome annotation. The young leaf, stem, and root tissues of A. gramineus were used for transcriptome sequencing in 2-4 biological replicates. For phylogenetic tree construction different numbers of species were selected as described in method section "Phylogenetic analyses"
Data exclusions	1. For the genome assembly, we searched against bacterial database to rule out any potential bacterial contamination. Contigs with an identity greater than 90% and an alignment of at least 80% were excluded<br><br>2. Before performing the transcriptome assembly, we retrieved high-quality reads by eliminating adaptor sequences and filtering low-quality reads using TRIMMOMATIC (v0.40).<br><br>3. To estimate the timing of whole-genome duplication events, low-copy families were exclusded based on pairwise comparison of paralog sequences.
Replication	The young leaf, stem, and root tissues of A. gramineus were used for transcriptome sequencing in 2-4 biological replicates. All attempts at replication were successful. One plant was used for DNA sequencing, and was successful.
Randomization	As we sequenced only one genome. Data randomization was not required.
Blinding	No experimental validation works were carried out. Hence, blinding was not applicable.

Reporting for specific materials, systems and methods

We require information from authors about some types of materials, experimental systems and methods used in many studies. Here, indicate whether each material, system or method listed is relevant to your study. If you are not sure if a list item applies to your research, read the appropriate section before selecting a response.

Materials & experimental systems

n/a	Involved in the study	
☒	☐	Antibodies
☒	☐	Eukaryotic cell lines
☒	☐	Palaeontology and archaeology
☒	☐	Animals and other organisms
☒	☐	Clinical data
☒	☐	Dual use research of concern
☐	☒	Plants

Methods

n/a	Involved in the study	
☒	☐	ChIP-seq
☒	☐	Flow cytometry
☒	☐	MRI-based neuroimaging

Dual use research of concern

Policy information about dual use research of concern

Hazards

Could the accidental, deliberate or reckless misuse of agents or technologies generated in the work, or the application of information presented in the manuscript, pose a threat to:

No	Yes	
☒	☐	Public health
☒	☐	National security
☒	☐	Crops and/or livestock
☒	☐	Ecosystems
☒	☐	Any other significant area

Experiments of concern

Does the work involve any of these experiments of concern:

No	Yes	
☒	☐	Demonstrate how to render a vaccine ineffective
☒	☐	Confer resistance to therapeutically useful antibiotics or antiviral agents
☒	☐	Enhance the virulence of a pathogen or render a nonpathogen virulent
☒	☐	Increase transmissibility of a pathogen
☒	☐	Alter the host range of a pathogen
☒	☐	Enable evasion of diagnostic/detection modalities
☒	☐	Enable the weaponization of a biological agent or toxin
☒	☐	Any other potentially harmful combination of experiments and agents
